# Development of Mitochondria-Targeted PARP Inhibitors

**DOI:** 10.3390/biom16010165

**Published:** 2026-01-19

**Authors:** Pavels Dimitrijevs, Marina Makrecka-Kuka, Pavel Arsenyan

**Affiliations:** Latvian Institute of Organic Synthesis, Aizkraukles 21, LV1006 Riga, Latvia

**Keywords:** PARP inhibitors, olaparib, rucaparib, cardiolipin binding, mitochondrial targeting, phosphonium conjugates

## Abstract

PARP inhibitors are a clinically validated class of anticancer therapeutics that exploit synthetic lethality to target homologous recombination-deficient tumors, such as those carrying BRCA1/2 mutations. Nevertheless, the rational design of mitochondria-targeted PARP inhibitors capable of selective mitochondrial accumulation and organelle-specific PARP modulation remains an unresolved objective. To enable organelle-specific modulation of PARP activity, we synthesized a series of trialkyl(aryl)phosphonium conjugates of olaparib and rucaparib designed to target mitochondria by cardiolipin binding. Their activity was evaluated by PARP1 inhibition, cardiolipin affinity, and cytotoxicity in BRCA1-deficient HCC1937 breast cancer cells and non-malignant H9C2 cardiomyocytes. All conjugates retained potent PARP1 inhibition (IC_50_ = 3.4–17 nM), comparable to the parent drugs. Several derivatives, particularly compounds **2d** and **6c**, exhibited strong cardiolipin binding (EC_50_ = 12.99 µM and 6.77 µM, respectively) and significantly enhanced cytotoxicity in HCC1937 cells (IC_50_ = 0.93 and 2.01 µM), outperforming olaparib and rucaparib. Notably, cytotoxicity toward H9C2 cells was lower, indicating a favorable selectivity profile. Phosphonium conjugation preserves PARP1 inhibitory activity while conferring mitochondrial targeting and enhanced anticancer potency. These findings support the development of mitochondria-targeted PARP inhibitors as a next-generation therapeutic strategy with the potential to improve efficacy and overcome resistance in HR-deficient tumors.

## 1. Introduction

Poly(ADP-ribose) polymerases, or PARPs, are a fascinating group of enzymes that rely on NAD^+^ to attach ADP-ribose units to proteins, playing a critical role in how cells respond to DNA damage. Among them, PARP1 stands out, driving about 90% of the cell’s PARylation activity and acting as a key player in spotting DNA single-strand breaks (SSBs) to start base excision repair (BER) in the nucleus [1]. PARP-1 exhibits high affinity for multiple DNA structural motifs, such as single- and double-strand breaks, crossover junctions, cruciform structures, supercoiled DNA, and selected double-stranded sequence elements. The discovery of small-molecule PARP inhibitors (PARPi), like olaparib and rucaparib, has represented a major advance in precision oncology. These drugs exploit a concept called synthetic lethality to target tumors with defects in homologous recombination, such as those with BRCA1/2 mutations, and have earned approval for treating ovarian, breast, pancreatic, and prostate cancers due to their impressive clinical results [2,3,4,5]. Yet, for all their success, current PARP inhibitors primarily focus on nuclear PARP1 and PARP2, leaving other cellular compartments, e.g., mitochondria, largely unexplored. Emerging research shows that PARP1 and PARP2 are not just confined to the nucleus; they are also active in mitochondria, where they influence critical processes like mtDNA repair, oxidative phosphorylation (OXPHOS), and even the initiation of apoptosis through pathways involving mitochondrial membrane potential and apoptosis inducing factor (AIF) [6,7,8]. When oxidative stress triggers excessive mitochondrial PARP activity, it can deplete NAD^+^ and disrupt energy production, triggering a pathogenic mechanism implicated in neurodegeneration, ischemia–reperfusion injury, and diabetes [9]. While clinically used agents such as olaparib and rucaparib can indirectly influence mitochondrial PARP activity, emerging evidence indicates that proteins on the mitochondrial surface, such as hexokinase [10], and mitochondrial inner membrane, such as mitofilin and respiratory complexes III and IV [11], are subject to PARylation. Consequently, the lack of dedicated mitochondrial targeting may limit the precision of organelle-specific action and the ability to directly modulate mitochondrial PARP activity. These observations have spurred the development of mitochondria-targeted PARP inhibitors (mtPARPi) designed to selectively accumulate within the mitochondrial matrix, thereby enabling organelle-specific modulation of PARP activity. By achieving subcellular precision, mtPARPi may offer therapeutic advantages over conventional PARPi, including enhanced efficacy against mitochondrial-dependent tumors, reduced nuclear off-target effects, and new applications beyond DNA repair-deficient cancers, particularly in conditions involving oxidative stress and mitochondrial damage. We have chosen to introduce positively charged phosphonium moiety to olaparib and rucaparib core and herein, we report the synthesis of trialkyl(aryl)phosphonium conjugates and demonstrate their specific binding to cardiolipin, a signature phospholipid of the mitochondrial inner membrane. We further assess their PARP1 inhibitory activity and their capacity to suppress the proliferation of HCC1937 cells in vitro.

## 2. Materials and Methods

### 2.1. General Information

Unless otherwise stated, all reagents were purchased from commercial suppliers and used without further purification. Ultrapure water was obtained from a Milli-Q system (18.2 MΩ cm). Thin layer chromatography (TLC) was performed using MERCK Silica gel 60 F254 plates and visualized by UV (254 nm) fluorescence (MERCK Co., Rahway, NJ, USA). ZEOCHEM silica gel (ZEOprep 60/35–70 μm—SI23501, Zeochem AG, Rüti, Switzerland) was used for column chromatography. ^1^H spectra were recorded on Bruker 300 or Bruker 400 spectrometer at 400 MHz (Bruker Corp., Billerica, MA, USA); ^13^C spectra were recorded on a Bruker 400 spectrometer at 101.3 MHz at 298 K in DMSO-*d6*, CDCl_3_, CD_3_OD, or the mixture of thereof. The ^1^H chemical shifts are given relatively to residual CHCl_3_ signal (7.26 ppm) CD_3_OD (3.31 ppm), or DMSO-*d6* (2.50 ppm); 13C—relatively to CDCl_3_ (77.16 ppm) or DMSO-*d6* (39.52 ppm) or CD_3_OD (49.0 ppm).

### 2.2. The Synthesis of ***2a***–***d*** and ***5a***

A vial charged with 4-(4-fluoro-3-(piperazine-1-carbonyl)benzyl)phthalazin-1(2H)-one (183 mg, 0.5 mmol) or rucaparib (0.5 mmol), an appropriate alkylating agent (0.5 mmol), diisopropylethylamine (65 mg, 0.6 mmol), 10 mL of THF, and 1 mL of NMP was heated at 50 °C for 48 h. Next, the volatiles were removed under reduced pressure and the resulting glue was purified by reverse-phase chromatography on C18 silica gel (eluent water (pH = 3, HCl)/acetonitrile in gradient from 5 to 80%).

(3-(4-(2-Fluoro-5-((4-oxo-3,4-dihydrophthalazin-1-yl)methyl)benzoyl)piperazin-1-yl)propyl)triphenylphosphonium chloride (**2a**). Obtained 180 mg, yield: 25%. ^1^H NMR (400 MHz, MeOD) δ 8.32 (dd, *J* = 7.9, 1.4 Hz, 1H), 7.98–7.74 (m, 18H), 7.51 (ddd, *J* = 8.5, 5.0, 2.2 Hz, 1H), 7.45 (dd, *J* = 6.4, 2.3 Hz, 1H), 7.16 (dd, *J* = 9.5, 8.6 Hz, 1H), 4.80–4.65 (m, 1H), 4.37 (s, 2H), 3.78–3.58 (m, 5H), 3.49 (t, *J* = 7.8 Hz, 2H), 3.27–3.02 (m, 2H), and 2.31–2.17 (m, 2H). ^13^C NMR (101 MHz, MeOD) δ 166.91, 162.17, 159.69, 157.23, 147.56, 136.54 (d, *J* = 3.0 Hz), 135.06, 135.00 (d, *J* = 10.1 Hz), 133.95 (d, *J* = 9.1 Hz), 132.9, 131.70 (d, *J* = 12.1 Hz), 130.83, 130.51 (d, *J* = 3.0 Hz) 129.15, 127.44, 126.76, 123.59 (d, *J* = 18.2 Hz), 119.57, 118.70, 117.25 (d, *J* = 22.2 Hz), 57.56 (d, *J* = 22.2 Hz), 53.01, 52.68, 44.93, 39.81, 38.10, 21.02, 20.48, and 18.83. ^19^F NMR (376 MHz, MeOD) δ −120.18–−120.37 (m). ^31^P NMR (162 MHz, MeOD) δ 23.67. HRMS: calcd. for C_41_H_39_FN_4_O_2_P^+^ [M+H]: 669.2795, found: 669.2812.

(6-(4-(2-fluoro-5-((4-oxo-3,4-dihydrophthalazin-1-yl)methyl)benzoyl)piperazin-1-yl)hexyl)triphenylphosphonium chloride (**2b**). Obtained 192 mg, yield: 24%. ^1^H NMR (400 MHz, MeOD) δ 8.41–8.29 (m, 1H), 8.00–7.94 (m, 1H), 7.93–7.73 (m, 17H), 7.52 (ddd, *J* = 8.5, 5.0, 2.3 Hz, 1H), 7.45 (dd, *J* = 6.3, 2.3 Hz, 1H), 7.19 (dd, *J* = 9.5, 8.6 Hz, 1H), 4.81–4.71 (m, 1H), 4.39 (s, 2H), 3.78–3.34 (m, 7H), 3.24–3.10 (m, 3H), 3.09–2.94 (m, 1H), 1.83–1.58 (m, 6H), and 1.45 (q, *J* = 7.6 Hz, 2H). ^13^C NMR (101 MHz, MeOD) δ 167.08, 162.31, 159.67, 157.22, 147.80, 136.49 (d, *J* = 4.0 Hz, minor conformer), 136.32 (d, *J* = 4.0 Hz, major conformer), 135.21, 134.77 (d, *J* = 10.1 Hz), 134.0 (d, *J* = 8.1 Hz), 133.96, 133.03, 131.57 (d, *J* = 13.1 Hz), 131.51, 130.79, 130.40 (d, *J* = 4.0 Hz), 129.05, 127.46, 126.80, 123.45 (d, *J* = 33.8 Hz), 123.34, 120.24, 119.38, 117.33 (d, *J* = 20.8 Hz), 117.21, 57.92, 52.67, 52.41, 45.03, 39.92, 38.08, 30.82 (d, *J* = 17.2 Hz), 30.74, 26.69, 24.58, 23.29 (d, *J* = 4.0 Hz), 22.89, and 22.38. ^19^F NMR (376 MHz, MeOD) δ −120.16 (q, *J* = 7.0 Hz). ^31^P NMR (162 MHz, MeOD) δ 23.69. HRMS: calcd. for C_44_H_45_FN_4_O_2_P^+^ [M+H]: 711.3264, found: 711.3269.

(10-(4-(2-fluoro-5-((4-oxo-3,4-dihydrophthalazin-1-yl)methyl)benzoyl)piperazin-1-yl)decyl)triphenylphosphonium chloride (**2c**). Obtained 206 mg, yield: 27%. ^1^H NMR (400 MHz, CDCl_3_) δ 8.34 (d, *J* = 7.7 Hz, 1H), 7.83–7.59 (m, 18H), 7.44–7.33 (m, 1H), 7.31–7.26 (m, 1H), 6.98 (t, *J* = 8.9 Hz, 1H), 4.72 (d, *J* = 14.2 Hz, 1H), 4.24 (s, 2H), 3.88 (t, *J* = 12.8 Hz, 1H), 3.65–3.49 (m, 3H), 3.48–3.22 (m, 3H), 3.15–2.89 (m, 7H), 1.90–1.71 (m, 2H), 1.65–1.42 (m, 4H), and 1.34–1.09 (m, 10H). ^13^C NMR (101 MHz, MeOD) δ 166.93, 162.19, 159.70, 157.25, 147.57, 136.53 (d, *J* = 4.0 Hz, minor conformer), 136.23 (d, *J* = 4.0 Hz, major conformer), 135.07, 134.80 (d, *J* = 10.1 Hz), 133.92 (d, *J* = 8.0 Hz), 132.87, 131.50 (d, *J* = 12.4 Hz), 130.84, 130.46 (d, *J* = 4.0 Hz), 129.16, 127.43, 126.78, 123.58 (d, *J* = 18.0 Hz), 120.41, 119.56, 117.22 (d, *J* = 21.0 Hz), 58.30, 58.14, 52.66, 52.40, 45.03, 39.90, 38.12, 31.55 (d, *J* = 17.2 Hz), 30.21, 30.01, 29.83, 27.50, 24.89, 23.54 (d, *J* = 4.0 Hz), 22.91, 22.40, and 18.38. ^19^F NMR (376 MHz, MeOD) δ −120.30 (q, *J* = 8.5 Hz). ^31^P NMR (162 MHz, MeOD) δ 23.81. HRMS: calcd. for C_48_H_53_FN_4_O_2_P^+^ [M+H]: 767.3890, found: 767.3892.

(12-(4-(2-fluoro-5-((4-oxo-3,4-dihydrophthalazin-1-yl)methyl)benzoyl)piperazin-1-yl)dodecyl)triphenylphosphonium chloride (**2d**). Obtained 159 mg, yield: 19%. ^1^H NMR (400 MHz, MeOD) δ 8.34 (dd, *J* = 7.8, 1.3 Hz, 1H), 8.00–7.93 (m, 1H), 7.93–7.85 (m, 4H), 7.85–7.71 (m, 13H), 7.52 (ddd, *J* = 8.6, 5.0, 1.9 Hz, 1H), 7.48–7.42 (m, 1H), 7.22–7.13 (m, 1H), 4.81–4.71 (m, 1H), 4.39 (s, 2H), 3.78–3.34 (m, 7H), 3.21–2.99 (m, 4H), 1.85–1.73 (m, 2H), 1.71–1.49 (m, 4H), and 1.44–1.22 (m, 14H). ^13^C NMR (101 MHz, MeOD) δ 166.94, 162.20, 159.71, 157.25, 147.57, 136.54 (d, *J* = 4.0 Hz, minor conformer), 136.24 (d, *J* = 4.0 Hz, major conformer), 135.07, 134.80 (d, *J* = 8.1 Hz), 133.94 (d, *J* = 8.0 Hz), 132.87, 131.51 (d, *J* = 12.4 Hz), 130.85, 130.48 (d, *J* = 4.0 Hz), 129.17, 127.45, 126.79, 123.56 (d, *J* = 7.0 Hz), 120.42, 119.56, 117.24 (d, *J* = 22.4 Hz), 58.18, 52.68, 52.41, 45.04, 39.91, 38.13, 31.69, 31.53, 30.53, 30.50, 30.43, 30.39, 30.13, 29.92, 27.57, 24.95, 23.57, 23.53, 22.92, and 22.41. ^19^F NMR (376 MHz, MeOD) δ −120.63 (q, *J* = 8.5 Hz). ^31^P NMR (162 MHz, MeOD) δ 23.81. HRMS: calcd. for C_50_H_57_FN_4_O_2_P^+^ [M+H]: 795.4203, found: 795.4199.

(6-((4-(8-Fluoro-1-oxo-2,3,4,6-tetrahydro-1H-azepino[5,4,3-cd]indol-5-yl)benzyl)(methyl)amino)hexyl)triphenylphosphonium chloride (**5a**). Obtained 188 mg, yield: 53%. ^1^H NMR (400 MHz, MeOD) δ 7.92–7.66 (m, 17H), 7.51 (dd, *J* = 10.8, 2.4 Hz, 1H), 7.33 (dd, *J* = 9.0, 2.4 Hz, 1H), 4.51 (d, *J* = 13.0 Hz, 1H), 4.32 (d, *J* = 13.0 Hz, 1H), 3.55–3.38 (m, 4H), 3.30–3.23 (m, 1H), 3.20–3.09 (m, 3H), 2.79 (s, 3H), 1.89–1.57 (m, 6H), and 1.50–1.38 (m, 2H). ^13^C NMR (101 MHz, MeOD) δ 172.27, 161.94, 159.59, 138.71 (d, *J* = 11.9 Hz), 136.90–135.77 (m), 135.01, 134.84 (d, *J* = 10.0 Hz), 132.84, 131.54 (d, *J* = 12.6 Hz), 130.10, 129.66, 126.26 (d, *J* = 8.8 Hz), 124.79, 120.33, 119.47, 114.04, 111.59 (d, *J* = 26.5 Hz), 102.44 (d, *J* = 26.2 Hz), 60.51, 56.94, 43.67, 40.15, 30.89 (d, *J* = 16.8 Hz), 30.00, 26.79, 24.98, 23.33 (d, *J* = 4.3 Hz), 22.93, and 22.42. ^31^P NMR (162 MHz, MeOD) δ 23.79. HRMS: calcd. for C_43_H_44_FN_3_OP^+^ [M+H]: 668.3206, found: 668.3221.

### 2.3. Procedure for the Synthesis of ***3a***–***g*** and ***6a***–***c***

To a solution of 4-(4-fluoro-3-(piperazine-1-carbonyl)benzyl)phthalazin-1(2H)-one (**1**, 0.3 mmol) or rucaparib (**4**, 0.3 mmol), appropriate phosphonium alkylcarboxylic acid (0.37 mmol), EDC hydrochloride (237 mg, 1.24 mmol), and HOBt (95 mg, 0.62 mmol) in 15 mL of DCM N-methylmorpholine (0.5 mL) was added. The resulting mixture was stirred for 2 h and then it was poured in 100 mL of ethyl acetate. The organic layer was washed with saturated NaCl solution and a few drops of HCl (2 × 50 mL) followed by saturated NaCl solution with 0.5 mL of sodium hydrogen carbonate solution (2 × 50 mL), then with water (50 mL). After drying over sodium sulphate organic layer was filtered through a silica gel pad and evaporated yielding the desired derivative.

(6-(4-(2-fluoro-5-((4-oxo-3,4-dihydrophthalazin-1-yl)methyl)benzoyl)piperazin-1-yl)-6-oxohexyl)trimethylphosphonium chloride (**3a**). Obtained 139 mg, yield: 78%. ^1^H NMR (400 MHz, MeOD) δ 8.33 (dd, *J* = 7.8, 1.4 Hz, 1H), 7.98–7.75 (m, 3H), 7.49 (dtd, *J* = 9.4, 4.7, 2.4 Hz, 1H), 7.43–7.33 (m, 1H), 7.15 (t, *J* = 9.0 Hz, 1H), 4.36 (s, 2H), 3.85–3.60 (m, 4H), 3.51 (q, *J* = 5.7 Hz, 2H), 3.39–3.25 (m, 2H), 2.45 (dt, *J* = 24.2, 7.3 Hz, 2H), 2.30–2.17 (m, 2H), and 1.73–1.43 (m, 6H). ^13^C NMR (101 MHz, MeOD) δ 173.94, 167.15 (d, *J* = 7.6 Hz), 162.11, 147.56, 136.36 (d, *J* = 3.4 Hz), 135.02, 133.42 (d, *J* = 8.0 Hz), 132.85, 130.78, 130.12 (d, *J* = 4.7 Hz), 127.08 (d, *J* = 62.6 Hz), 124.60 (d, *J* = 20.2 Hz), 117.16 (d, *J* = 21.7 Hz), 48.05 (d, *J* = 42.0 Hz), 46.44 (d, *J* = 55.0 Hz), 43.61–41.93 (m), 38.12, 33.46, 31.16 (d, *J* = 16.4 Hz), 25.44, 23.90 (d, *J* = 52.6 Hz), 22.12 (d, *J* = 4.2 Hz), and 7.86 (d, *J* = 55.0 Hz). ^19^F NMR (376 MHz, MeOD) δ −120.60 (q, *J* = 8.5 Hz). ^31^P NMR (162 MHz, MeOD) δ 27.29. HRMS: calcd. for C_29_H_37_FN_4_O_3_P^+^ [M+H]: 539.2587, found: 539.2603.

(10-(4-(2-Fluoro-5-((4-oxo-3,4-dihydrophthalazin-1-yl)methyl)benzoyl)piperazin-1-yl)-10-oxodecyl)trimethylphosphonium chloride (**3b**). Obtained 59 mg, yield: 9%. ^1^H NMR (400 MHz, MeOD) δ 8.41–8.30 (m, 1H), 7.99–7.92 (m, 1H), 7.92–7.78 (m, 2H), 7.54–7.45 (m, 1H), 7.41–7.33 (m, 1H), 7.16 (t, *J* = 9.0 Hz, 1H), 4.38 (s, 2H), 3.85–3.60 (m, 4H), 3.56–3.44 (m, 2H), 3.37–3.23 (m, 2H), 2.40 (dt, *J* = 25.5, 7.5 Hz, 2H), 2.25–2.15 (m, 2H), 1.86 (d, *J* = 14.5 Hz, 9H), and 1.68–1.28 (m, 15H). ^13^C NMR (101 MHz, MeOD) δ 174.42, 167.27 (d, *J* = 9.1 Hz), 162.23, 157.17, 147.61, 136.43 (d, *J* = 3.4 Hz), 135.01, 133.43 (d, *J* = 8.3 Hz), 132.85, 130.87, 130.10, 129.21, 127.13 (d, *J* = 68.8 Hz), 117.18 (d, *J* = 21.8 Hz), 47.90, 46.50 (d, *J* = 53.3 Hz), 43.72–41.97 (m), 38.17, 33.94, 31.67 (d, *J* = 16.2 Hz), 30.65–30.19 (m), 29.91, 26.36, 24.02 (d, *J* = 52.4 Hz), 22.33 (d, *J* = 4.4 Hz), and 7.77 (d, *J* = 55.1 Hz). ^19^F NMR (376 MHz, MeOD) δ −120.71 (q, *J* = 8.5 Hz). ^31^P NMR (162 MHz, MeOD) δ 27.19. HRMS: calcd. for C_33_H_45_FN_4_O_3_P^+^ [M+H]: 595.3213, found: 595.3207.

Tributyl(6-(4-(2-fluoro-5-((4-oxo-3,4-dihydrophthalazin-1-yl)methyl)benzoyl)piperazin-1-yl)-6-oxohexyl)phosphonium chloride (**3c**) Obtained 150 mg, yield: 71%. ^1^H NMR (400 MHz, MeOD) δ 8.34 (dd, *J* = 7.9, 1.4 Hz, 1H), 7.95 (dd, *J* = 8.1, 3.9 Hz, 1H), 7.91–7.77 (m, 2H), 7.54–7.45 (m, 1H), 7.41–7.34 (m, 1H), 7.15 (t, *J* = 9.0 Hz, 1H), 4.37 (s, 2H), 3.83–3.76 (m, 1H), 3.72 (dd, *J* = 7.0, 3.7 Hz, 1H), 3.66 (dt, *J* = 7.7, 3.8 Hz, 2H), 3.51 (p, *J* = 4.5 Hz, 2H), 3.39–3.33 (m, 1H), 3.30–3.24 (m, 1H), 2.46 (dt, *J* = 24.2, 7.2 Hz, 2H), 2.30–2.16 (m, 8H), 1.72–1.44 (m, 19H), and 0.99 (td, *J* = 7.1, 2.3 Hz, 9H). ^13^C NMR (101 MHz, MeOD) δ 173.94, 167.17 (d, *J* = 8.7 Hz), 162.13, 147.56, 136.38 (d, *J* = 3.3 Hz), 135.03, 133.42 (d, *J* = 8.0 Hz), 132.84, 130.81, 130.07 (d, *J* = 5.4 Hz), 129.15, 127.41, 126.79, 124.63 (d, *J* = 18.2 Hz), 117.15 (d, *J* = 21.8 Hz), 48.05 (d, *J* = 42.1 Hz), 46.44 (d, *J* = 55.8 Hz), 43.66–41.74 (m), 38.14, 33.47, 31.33 (d, *J* = 15.3 Hz), 25.41, 24.94 (d, *J* = 15.6 Hz), 24.35 (d, *J* = 4.5 Hz), 22.19 (d, *J* = 4.4 Hz), 19.16 (dd, *J* = 47.9, 17.6 Hz), and 13.71. ^19^F NMR (376 MHz, MeOD) δ −120.57 (q, *J* = 8.5 Hz). ^31^P NMR (162 MHz, MeOD) δ 33.45. HRMS: calcd. for C_38_H_55_FN_4_O_3_P^+^ [M+H]: 665.3996, found: 665.3996.

Tributyl(10-(4-(2-fluoro-5-((4-oxo-3,4-dihydrophthalazin-1-yl)methyl)benzoyl)piperazin-1-yl)-10-oxodecyl)phosphonium chloride (**3d**) Obtained 172 mg, yield: 79%. ^1^H NMR (400 MHz, MeOD) δ 8.35 (dd, *J* = 7.8, 1.5 Hz, 1H), 7.95 (dd, *J* = 8.4, 2.6 Hz, 1H), 7.85 (dqd, *J* = 22.0, 7.2, 1.9 Hz, 2H), 7.49 (tt, *J* = 5.1, 2.5 Hz, 1H), 7.38 (dt, *J* = 5.8, 2.6 Hz, 1H), 7.16 (t, *J* = 9.0 Hz, 1H), 4.38 (s, 2H), 3.83–3.60 (m, 4H), 3.50 (dt, *J* = 10.4, 4.9 Hz, 2H), 3.37–3.32 (m, 1H), 3.28 (d, *J* = 6.3 Hz, 1H), 2.41 (dt, *J* = 24.6, 7.5 Hz, 2H), 2.28–2.16 (m, 8H), 1.54 (dtd, *J* = 25.1, 8.1, 4.4 Hz, 19H), 1.43–1.29 (m, 8H), and 0.99 (t, *J* = 7.0 Hz, 9H). ^13^C NMR (101 MHz, MeOD) δ 174.40, 167.16, 162.17, 147.58, 136.38 (d, *J* = 3.5 Hz), 135.01, 133.41 (d, *J* = 8.0 Hz), 132.84, 130.83, 130.09, 129.16, 127.11 (d, *J* = 63.9 Hz), 124.53, 117.16 (d, *J* = 21.7 Hz), 48.06 (d, *J* = 40.7 Hz), 46.51 (d, *J* = 53.7 Hz), 43.54–41.87 (m), 38.14, 33.91, 31.77 (d, *J* = 15.1 Hz), 30.71–30.02 (m), 29.84, 26.34, 24.94 (d, *J* = 15.4 Hz), 24.36 (d, *J* = 4.6 Hz), 22.33 (d, *J* = 4.5 Hz), 19.18 (dd, *J* = 47.9, 22.9 Hz), and 13.70. ^19^F NMR (376 MHz, MeOD) δ −120.60 (q, *J* = 8.5 Hz). ^31^P NMR (162 MHz, MeOD) δ 33.41. HRMS: calcd. for C_42_H_63_FN_4_O_3_P^+^ [M+H]: 721.4622, found: 721.4639.

(4-(4-(2-Fluoro-5-((4-oxo-3,4-dihydrophthalazin-1-yl)methyl)benzoyl)piperazin-1-yl)-4-oxobutyl)triphenylphosphonium chloride (**3e**). Obtained 146 mg, yield: 69%. ^1^H NMR (400 MHz, MeOD) δ 8.40–8.30 (m, 1H), 8.00–7.69 (m, 18H), 7.54–7.45 (m, 1H), 7.42–7.33 (m, 1H), 7.16 (t, *J* = 9.0 Hz, 1H), 4.38 (s, 2H), 3.82–3.65 (m, 3H), 3.64–3.39 (m, 5H), 3.36–3.27 (m, 2H), 2.69 (dt, *J* = 25.2, 6.4 Hz, 2H), and 2.02–1.81 (m, 2H). ^13^C NMR (101 MHz, MeOD) δ 172.17, 167.28 (d, *J* = 9.8 Hz), 147.62, 136.46 (d, *J* = 3.4 Hz), 136.33 (d, *J* = 3.1 Hz), 135.01 (d, *J* = 3.8 Hz), 134.86 (d, *J* = 10.0 Hz), 133.47 (d, *J* = 8.3 Hz), 132.87, 131.55 (d, *J* = 12.6 Hz), 130.09, 127.48, 126.78, 120.28, 119.42, 117.18 (d, *J* = 21.7 Hz), 47.92 (d, *J* = 23.0 Hz), 46.11 (d, *J* = 56.0 Hz), 43.41–41.98 (m), 38.17, 33.53 (d, *J* = 17.8 Hz), 22.46 (d, *J* = 52.2 Hz), and 19.37. ^19^F NMR (376 MHz, MeOD) δ −120.70 (q, *J* = 8.5 Hz). ^31^P NMR (162 MHz, MeOD) δ 23.74. HRMS: calcd. for C_42_H_49_FN_4_O_3_P^+^ [M+H]: 697.2744, found: 697.2739.

(6-(4-(2-Fluoro-5-((4-oxo-3,4-dihydrophthalazin-1-yl)methyl)benzoyl)piperazin-1-yl)-6-oxohexyl)triphenylphosphonium chloride (**3f**) ^1^H NMR (400 MHz, MeOD) δ 8.36 (dd, *J* = 7.8, 2.1 Hz, 1H), 7.99–7.70 (m, 18H), 7.50 (s, 1H), 7.36 (t, *J* = 6.4 Hz, 1H), 7.17 (t, *J* = 9.0 Hz, 1H), 4.39 (s, 2H), 3.79–3.55 (m, 4H), 3.53–3.34 (m, 4H), 3.27 (s, 1H), 2.47–2.32 (m, 2H), and 1.65 (d, *J* = 35.3 Hz, 6H). ^13^C NMR (101 MHz, MeOD) δ 173.90, 167.35, 136.48, 136.29 (d, *J* = 3.0 Hz), 135.03, 134.84 (d, *J* = 10.1 Hz), 133.48 (d, *J* = 8.3 Hz), 132.88, 131.53 (d, *J* = 12.6 Hz), 130.06, 127.14 (d, *J* = 69.5 Hz), 119.96 (d, *J* = 86.3 Hz), 117.19 (d, *J* = 21.8 Hz), 47.86, 43.54–41.96 (m), 38.17, 33.35, 31.14 (d, *J* = 16.5 Hz), 25.31, 23.35 (d, *J* = 4.2 Hz), and 22.60 (d, *J* = 51.3 Hz). HRMS: calcd. for C_44_H_43_FN_4_O_3_P^+^ [M+H]: 725.3057, found: 725.3069.

(10-(4-(2-fluoro-5-((4-oxo-3,4-dihydrophthalazin-1-yl)methyl)benzoyl)piperazin-1-yl)-10-oxodecyl)triphenylphosphonium chloride (**3g**) Obtained 60 mg, yield: 26%. ^1^H NMR (400 MHz, MeOD) δ 8.35 (dd, *J* = 7.7, 1.4 Hz, 1H), 8.08–7.69 (m, 18H), 7.54–7.45 (m, 1H), 7.41–7.33 (m, 1H), 7.16 (dd, *J* = 9.4, 8.6 Hz, 1H), 4.38 (s, 2H), 3.81–3.59 (m, 4H), 3.54–3.34 (m, 2H), 3.30–3.25 (m, 1H), 2.39 (dt, *J* = 25.7, 7.5 Hz, 2H), 1.70–1.60 (m, 2H), 1.59–1.51 (m, 4H), and 1.34–1.26 (m, 9H). ^13^C NMR (101 MHz, MeOD) δ 136.27 (d, *J* = 3.0 Hz), 135.01, 134.81 (d, *J* = 9.9 Hz), 133.44 (d, *J* = 8.4 Hz), 132.85, 131.52 (d, *J* = 12.6 Hz), 130.88, 130.11, 129.22, 127.13 (d, *J* = 69.7 Hz), 124.60 (d, *J* = 22.9 Hz), 120.01 (d, *J* = 86.4 Hz), 117.18 (d, *J* = 21.7 Hz), 47.90, 46.50 (d, *J* = 52.4 Hz), 44.25–41.80 (m), 38.18, 33.91, 31.54 (d, *J* = 16.1 Hz), 30.20 (d, *J* = 10.0 Hz), 29.75, 26.31, 23.49 (d, *J* = 4.4 Hz), and 22.64 (d, *J* = 51.0 Hz). HRMS: calcd. for C_48_H_51_FN_4_O_3_P^+^ [M+H]: 781.3683, found: 781.3698.

(6-((4-(8-Fluoro-1-oxo-2,3,4,6-tetrahydro-1H-azepino[5,4,3-cd]indol-5-yl)benzyl)(methyl)amino)hexyl)trimethylphosphonium chloride (**6a**) Obtained 85 mg, yield: 73%. ^1^H NMR (400 MHz, MeOD) appears as mixture of two conformers δ 7.68–7.48 (m, 4H), 7.42–7.28 (m, 4H), 4.71 (s, 1H, minor), 4.66 (s, 2H major), 3.56–3.52 (m, 3H), 3.14 (m, 3H), 3.05 (s, 2.7H, major), 2.98 (s, 1.3H, minor), 2.52 (q, *J* = 7.8 Hz, 3H), 2.29–2.13 (m, 13H), 1.92–1.78 (m, 13H), and 1.78–1.42 (m, 9H). ^13^C NMR (101 MHz, MeOD) δ 175.72, 175.47, 172.53, 161.75, 159.43 (d, *J* = 5.1 Hz), 138.65, 138.50 (d, *J* = 5.5 Hz), 137.14 (d, *J* = 3.6 Hz), 136.89, 132.73, 132.48, 129.60, 129.31 (d, *J* = 7.0 Hz), 128.11, 125.87, 124.98, 113.17, 113.00, 111.47 (d, *J* = 7.7 Hz), 111.21 (d, *J* = 7.7 Hz), 102.34 (d, *J* = 26.0 Hz), 54.16, 51.66, 43.80, 35.75, 34.64, 33.82, 33.55, 31.35 (d, *J* = 5.3 Hz), 31.19 (d, *J* = 5.3 Hz), 30.00, 25.61, 25.37, 24.20 (d, *J* = 3.8 Hz), 23.67 (d, *J* = 3.9 Hz), 22.20 (t, *J* = 3.8 Hz), 8.04 (d, *J* = 2.8 Hz), and 7.49 (d, *J* = 2.9 Hz). ^19^F NMR (376 MHz, MeOD) δ −123.02–−123.08 (m, minor), −123.18–−123.23 (m, major). ^31^P NMR (162 MHz, MeOD) δ 27.29 (s, major), 27.25 (s, minor). HRMS: calcd. for C_28_H_36_FN_3_O_2_P^+^ [M+H]: 496.2529, found: 496.2538.

(6-((4-(8-fluoro-1-oxo-2,3,4,6-tetrahydro-1H-azepino [5,4,3-cd]indol-5-yl)benzyl)(methyl)amino)-6-oxohexyl)triphenylphosphonium chloride (**6b**) Obtained 179 mg, yield: 83%. ^1^H NMR (400 MHz, MeOD) appears as mixture of two conformers δ 7.93–7.66 (m, 14H), 7.65–7.47 (m, 3H), 7.41–7.35 (m, 1H), 7.35–7.27 (m, 1H), 4.68 (s, 0.6H, minor), 4.64 (s, 1H, major), 3.57–3.49 (m, 1H), 3.48–3.33 (m, 2H), 3.17–3.08 (m, 2H), 3.01 (s, 1.5H, major), 2.97 (s, 1H, minor), 2.50–2.39 (m, 2H), and 1.79–1.51 (m, 6H). ^13^C NMR (101 MHz, MeOD) δ 175.67, 175.37, 172.52, 161.74, 159.40, 138.64, 138.52, 138.48, 138.09, 137.13, 136.31, 136.28, 136.25, 134.87, 134.82, 134.77, 134.72, 132.69, 132.47, 131.60, 131.57, 131.47, 131.45, 129.58, 129.34, 129.30, 128.06, 124.99, 120.38, 120.36, 120.33, 119.53, 119.50, 119.47, 113.17, 113.00, 111.46, 111.29, 111.20, 102.52, 102.26, 54.17, 51.67, 43.81, 43.78, 35.74, 34.78, 33.69, 33.43, 31.27, 31.18, 31.11, 31.02, 30.00, 29.97, 25.42, 25.23, 23.40, 23.36, 23.31, 23.27, 22.89, 22.81, 22.38, and 22.29. ^19^F NMR (376 MHz, MeOD) δ −122.92–−122.97 (m, minor), −123.11–−123.16 (m, major). ^31^P NMR (162 MHz, MeOD) δ 23.80 (s, major), 23.75 (s, minor). HRMS: calcd. for C_43_H_42_FN_3_O_2_P^+^ [M+H]: 682.2999, found: 682.2995.

(10-((4-(8-Fluoro-1-oxo-2,3,4,6-tetrahydro-1H-azepino[5,4,3-cd]indol-5-yl)benzyl)(methyl)amino)-10-oxodecyl)triphenylphosphonium chloride (**6c**). Obtained 180 mg, yield: 77%. ^1^H NMR (400 MHz, MeOD) δ appears as mixture of two conformers 7.86 (tt, *J* = 6.9, 1.9 Hz, 3H), 7.82–7.68 (m, 12H), 7.59 (dd, *J* = 22.6, 7.9 Hz, 2H, major), 7.50 (dd, *J* = 10.7, 2.3 Hz, 1H, minor), 7.35 (dd, *J* = 13.7, 7.9 Hz, 2H, major), 7.30 (ddd, *J* = 9.0, 2.3, 1.2 Hz, 1H, minor), 4.69 (s, 0.7H, minor), 4.63 (s, 1.3H, major), 3.50 (d, *J* = 5.8 Hz, 2H), 3.41–3.32 (m, 2H), 3.30–3.23 (m, 1H), 3.12–3.08 (m, 1H), 3.03 (s, 1.8H, major), 2.98 (s, 1H, minor), 2.45 (t, *J* = 7.5 Hz, 1.2H, major), 2.40 (t, *J* = 7.5 Hz, 0.8H, minor), and 1.69–1.15 (m, 14H). ^13^C NMR (101 MHz, MeOD) δ 176.24, 175.89, 161.71, 159.36, 138.52, 138.24, 137.11 (d, *J* = 3.6 Hz), 136.26 (d, *J* = 3.1 Hz), 134.81 (d, *J* = 2.7 Hz), 132.51 (d, *J* = 21.3 Hz), 131.51 (d, *J* = 12.6 Hz), 129.55, 129.29, 128.05, 124.97, 120.37 (d, *J* = 4.4 Hz), 119.51 (d, *J* = 4.4 Hz), 113.06 (d, *J* = 14.9 Hz), 111.87–110.68 (m), 102.32 (d, *J* = 26.3 Hz), 54.28, 51.60, 43.76, 35.84, 34.86, 34.24, 33.91, 31.60 (d, *J* = 4.3 Hz), 31.44 (d, *J* = 4.5 Hz), 30.81–29.37 (m), 26.39, 26.25, 23.49 (d, *J* = 4.2 Hz), 22.90, and 22.39. ^19^F NMR (376 MHz, MeOD) δ −122.74–−122.93 (m, minor), −122.99–−123.10 (m, major). ^31^P NMR (162 MHz, MeOD) δ 23.79 (s, major), 23.75 (s, minor). HRMS: calcd. for C_47_H_50_FN_3_O_2_P^+^ [M+H]: 738.3625, found: 738.3632.

### 2.4. PARP1 Activity Assay

PARP1 activity was evaluated using 96-well plate chemiluminescent PARP1 assay kit (BPS Bioscience, San Diego, CA, USA, cat. # 80551) according to the manufacturer’s instructions. Stock solutions of tested compounds (10 mM in DMSO) were diluted to 30 μM and then to 3 μM in PARP assay buffer. For a titration, serial dilutions were prepared from 3 to 0.03 μM (10-fold higher concentration than the desired final concentration in the assay). Luminescence was measured using a Hidex Sense microplate reader (Hidex Finland, Turku, Finland).

### 2.5. Measurement of Affinity for Cardiolipin

A 10 mM stock solution of MitoCLue (3,6-di(azetidin-1-yl)-10-(3-(trimethylsilyl)propyl)acridin-10-ium iodide, CAS 2648425-06-1) was prepared in DMSO and diluted to 50 µM in 20 mM HEPES buffer (pH 6.8). Liposomes composed of cardiolipin (CL) and 1,2-dioleoyl-sn-glycero-3-phosphocholine (DOPC) at 25 µM and 75 µM, respectively, were prepared using the thin-film hydration method [12].

The competitive binding assay was performed in black 96-well plates (Sarstedt, Nümbrecht, Germany). CL-containing liposomes (10 µL; final CL concentration 2.5 µM) were added to each well in triplicate and titrated with the test ligand. Mixing was performed gently by pipetting up and down. Vehicle controls (vesicles without a ligand) were included in triplicate. Plates were incubated at 37 °C for 10 min in a plate shaker–thermostat (500 rpm). MitoCLue solution (10 µL; final concentration 5 µM; final volume 100 µL) was then added to each well and gently mixed with a pipette. Plates were incubated again at 37 °C for 15 min (500 rpm), and fluorescence intensity was measured on a Tecan Infinite 1000 plate reader (λ_exc_ = 497 nm, λ_em_ = 529 nm).

Data were analyzed using GraphPad Prism^®^ 10.4. Fluorescence values were normalized by setting CL liposomes with MitoCLue in the absence of ligand (vehicle) as 0% and the maximal ligand response as 100%. Normalized fluorescence intensity was plotted against ligand concentration on a logarithmic scale and fitted using a four-parameter logistic (4PL) or biphasic model to determine EC_50_ values.

### 2.6. Cell Cultures

HCC1937 (human breast carcinoma, BRCA1 (mutated, insertion C at nucleotide 5382) and H9C2 (CRL-1446™, subclone of the original clonal cell line derived from embryonic BD1X rat heart tissue) were obtained from the American Type Culture Collection (ATCC) (Manassas, VA, USA) for use in the current study. Cell lines were cultured at 37 °C and 5% CO_2_ in Dulbecco’s modified Eagle’s medium (DMEM) containing 10% fetal bovine serum (FBS) and 1% Pen/Strep according to recommendations.

### 2.7. Cytotoxicity Assay

Cell viability was assessed by the 3-(4,5-dimethylthiazol-2-yl)-2,5-diphenyltetrazolinium bromide (MTT) assay. Briefly, cells were seeded (5 × 10^3^ cells per well) in 96-well plates and allowed to attach for 24 h. Solutions of test compounds were prepared and serially diluted to obtain the appropriate concentrations. The cells were treated with test compounds at different concentrations (0.005–100 μM) and incubated for 48 h at 37 °C and 5% CO_2_. Then, the culture medium was removed, and the medium containing 1 mg/mL MTT was added. After 1 h the MTT-containing medium was removed, and 100 μL of isopropanol were immediately added to each sample. The optical density was assessed at 570/650 nm (Hidex Sense microplate reader, Hidex Finland). Experiments were performed in biological triplicates, each comprising three technical replicates. The half-maximal inhibitory concentration (IC_50_) of each compound was calculated using GraphPad Prism^®^ 10.4 (GraphPad, Inc., La Jolla, CA, USA).

### 2.8. Measurements of Mitochondrial Respiration and H_2_O_2_ Producation Rate in Permeabilized Cardiac Fibers

Mitochondrial function was assessed in permeabilized cardiac fibers from the right ventricle that were prepared as previously described [13]. The fibers were preincubated with **2c** or **3g** at 1 µM concentration for 15 min. The mitochondrial respiration measurements were performed in MiR05 media (110 mM sucrose, 60 mM K-lactobionate, 0.5 mM EGTA, 3 mM MgCl_2_, 20 mM taurine, 10 mM KH_2_PO_4_, 20 mM HEPES, pH 7.1, 0.1% BSA essentially free of fatty acids) at 37 °C using an Oxygraph-2k (O2k; Oroboros Instruments, Innsbruck, Austria). Mitochondrial functionality measurements were performed using the following protocol: pyruvate, malate, and succinate (5 mM, 0.5 mM, and 10 mM, respectively) were used to measure mitochondrial respiration in a complex I and II substrate-dependent LEAK state. Then, ADP (5 mM) was added to initiate oxidative phosphorylation-dependent respiration (OXPHOS state). Then, antimycin A (2.5 μM, complex III inhibitor) was added to determine residual oxygen consumption [14]. The flux of H_2_O_2_ was measured simultaneously by the O2k-Fluorometer module using the H_2_O_2_ sensitive probe Amplex ultra Red (AmR) as previously described [15]. Briefly, the assay medium was supplemented with AmR (5 μM), horseradish peroxidase (2 U/mL), and Cu,Zn-superoxide dismutase (2 U/mL) for quantitative conversion of the released O_2_^•−^ into H_2_O_2_. Fluorescence of the resorufin, the product of the AmR oxidation, was monitored at 590 nm (λ_Exc_ = 560 nm). The system was calibrated by adding known amounts of H_2_O_2_ (Sigma-Aldrich) in 0.1 μM steps. The H_2_O_2/_O flux ratio [%] was calculated as the H_2_O_2_ flux/(0.5 O_2_ flux).

## 3. Results

**Scheme 1:**
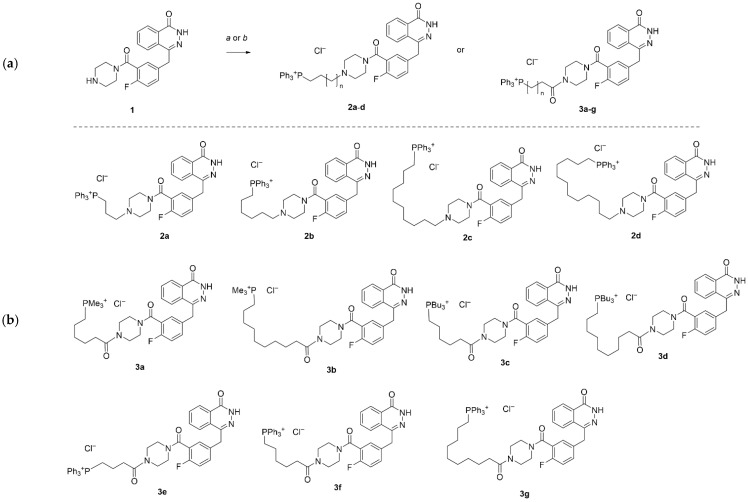
Synthesis of analogs of olaparib. Reaction conditions: (**a**) alkylating agent (1 equiv.), diisopropylethylamine (1.2 equiv.), and THF/NMP at 50 °C for 48 h; (**b**) appropriate phosphonium alkylcarboxylic acid (1.25 equiv.), EDC hydrochloride (4 equiv.), and HOBt (2 equiv.) DCM/N-methylmorpholine at ambient temperature for 2 h.

**Scheme 2:**
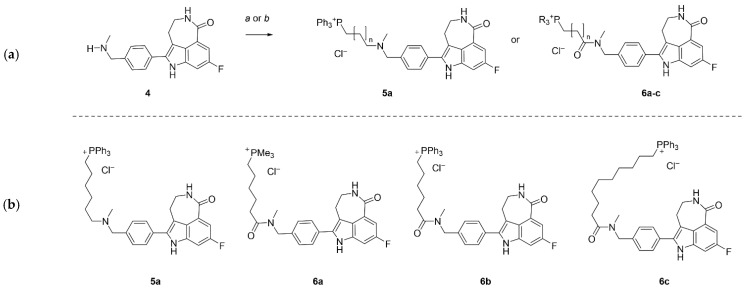
Synthesis of analogs of rucaparib. *Reaction conditions*: (**a**) alkylating agent (1 equiv.), diisopropylethylamine (1.2 equiv.), and THF/NMP at 50 °C for 48 h; (**b**) appropriate phosphonium alkylcarboxylic acid (1.25 equiv.), EDC hydrochloride (4 equiv.), and HOBt (2 equiv.) DCM/N-methylmorpholine at ambient temperature for 2 h.

The synthetic design of the trialkyl(aryl)phosphonium conjugates of olaparib and rucaparib was guided by two key considerations: preservation of the PARP1-binding pharmacophore to retain inhibitory potency, and the introduction of a mitochondria-targeting moiety to promote organelle-specific accumulation. To achieve this, we exploited peripheral positions on the olaparib and rucaparib scaffolds that are chemically accessible and spatially distant from the NAD^+^ binding site (Scheme 1 and Scheme 2).

We modified the substituent in the piperazine fragment of olaparib by direct alkylation or acylation of commercially available 4-(4-fluoro-3-(piperazine-1-carbonyl)benzyl)phthalazin-1(2H)-one (**1**) (Scheme 1). Alkylation was carried out by heating of **1** with the corresponding bromoalkyl phosphonium bromides in a THF/NMP mixture using diisopropylethylamine as base for two days. It was confirmed experimentally that all reagents should be used in equimolar concentrations to minimize the formation of side products. Crude products were purified by reverse-phase chromatography, yielding the desired products **2a**–**d** in modest yields (19–27%). Derivatives **3a**–**g** were prepared by the direct acylation of **1** with appropriate phosphonioalkyl carboxylic acid in the presence of EDC hydrochloride and HOBt. Varying the linker length and flexibility between the core and the phosphonium moiety (as in compounds **2a**–**d** and **3a**–**g**) allowed us to modulate lipophilicity, charge distribution, and conformational freedom. Rucaparib analog **5a** was obtained in 53% yield using the procedure for the synthesis of **2a**–**d**. Analogous acylations of **4** allowed us to obtain derivatives **6a**–**c** in good yields (Scheme 2). Copies of ^1^H, ^13^C, ^19^F, and ^31^P NMR and HRMS spectra are included in Supplementary Materials.

- **PARP1 Inhibition**

All synthesized olaparib derivatives exhibited strong PARP1 inhibitory activity (Table 1), with IC_50_ values in the low nanomolar range (3.4–17 nM), comparable to the parent compound (3.10 ± 0.21 nM). Notably, compounds **3a–g** demonstrated the most potent inhibition (3.4–7.6 nM), indicating that introduction of the phosphonium moiety at the periphery of the pharmacophore does not interfere with PARP1 binding. In contrast, the alkyl-substituted derivatives **2a–d** showed slightly reduced potency (IC_50_ = 8.2–17.0 nM) compared to their acyl-substituted analogs, suggesting that the presence of a lone pair on the piperazine nitrogen negatively affects PARP1 inhibition.

In turn, modification of the secondary amino group of rucaparib had a marked effect on the ability to suppress PARP1 activity. The most potent compound in this series, **5a,** was almost seven-fold less active than rucaparib (IC_50_ = 13.20 ± 1.79 versus 1.93 ± 0.29 nM). Within the series, alkyl-substituted derivative **5a** was significantly more potent than acyl-substituted analogs **6b** (87.84 ± 7.01 nM). Furthermore, introduction of the bulkier phosphonium cation (triphenyl versus trimethyl) and longer alkyl chain (C6 versus C10) reduced PARP1 inhibitory activity, indicating that steric hindrance significantly limits enzyme binding in the rucaparib scaffold, in contrast to the olaparib series, where this effect is less pronounced.

- **Mitochondrial Targeting via Cardiolipin Binding**

Cardiolipin (CL) is a signature phospholipid of mitochondria, representing approximately 20–25% of total phospholipids in the inner mitochondrial membrane and contributing to the membrane’s overall negative charge [16]. Triphenylphosphonium derivatives are known to bind to CL [17], and their mitochondrial targeting ability is likely driven by this interaction. To assess the affinity of novel compounds for CL, we used a MitoCLue assay, which measures a displacement of a CL-specific fluorescent probe (MitoCLue) from the CL-containing liposomal membrane model by the test compounds [12]. Olaparib and rucaparib showed no measurable binding. The presence of a triphenylphosphonium moiety was found to be essential for CL binding, as analogs containing trimethyl- or tributylphosphonium groups exhibited only weak affinity (EC_50_ > 400 µM). Extending the alkyl chain length markedly enhanced affinity for CL, obviously, by improving lipophilic interactions with acyl chains of CL. Particularly, the EC_50_ decreased more than forty-fold between C3 and C12 alkyl tails and more than four-fold between C10 and C12 alkyl tails. Additionally, compound **2c**, bearing an alkyl-substituted piperazine ring in the olaparib scaffold, exhibited twice higher affinity than its acyl-substituted analogue **3g**.

In rucaparib scaffold, C6 alkyl chain was sufficient to ensure high to moderate affinity for CL (EC_50_ = 11.85 ± 0.85 µM for **5a** and 25.07 ± 0.54 µM for **6b**), while **6c** with C10 tail had the highest affinity (EC_50_ = 6.77 ± 0.09 µM) in the series. Consistent with the olaparib-based series, **6a** with trimethylphsphonium group had weak interaction with CL (EC_50_ > 400 µM), and **5a** with alkyl-substituted amino group displayed approximately two-fold higher affinity than its acyl-substituted counterpart **6b**.

Collectively, these findings highlight that structural modifications significantly influence mitochondrial affinity, primarily through alterations in phosphonium charge distribution, tail lipophilicity, and basicity of substituted nitrogen.

- **Cytotoxicity in HCC1937 and H9C2 Cells**

Cytotoxicity was evaluated in BRCA1-deficient HCC1937 breast cancer cells and non-malignant H9C2 cardiomyocytes. The parent drugs olaparib and rucaparib exhibited no or weak cytotoxicity in these cell lines (IC_50_ > 100 µM and 32 µM, respectively). Interestingly, comparison of PARP1 inhibitory potency with cytotoxic activity revealed no apparent correlation between enzymatic inhibition and cell viability. Several of the most potent PARP1 inhibitors (IC_50_ < 10 nM), including compounds **3a**–**3c**, showed negligible cytotoxicity in both HCC1937 and H9C2 cells. In contrast, compounds with only moderate or weak PARP1 inhibition, such as **2c**, **2d**, and **6c**, displayed pronounced cytotoxicity in HCC1937 cells, with IC_50_ values in the low micromolar range. These findings indicate that cytotoxicity in this series is not primarily driven by PARP inhibition and point to an additional mechanism of action.

In contrast, a clear relationship emerged between CL affinity and cytotoxic potential. Compounds exhibiting high (EC_50_ < 20 µM) or moderate (EC_50_ 20–150 µM) affinity for CL were the most cytotoxic, whereas weak or non-binding analogs (EC_50_ > 400 µM) were largely inactive. This correlation suggests that interaction with CL in the inner mitochondrial membrane contributes to the observed cytotoxic effects, likely through perturbation of mitochondrial function or membrane integrity. However, the most potent CL binders, including **2d** and **6c**, also showed the lowest selectivity toward malignant cells, while compounds with moderate CL affinity, such as **3g** and **6b**, exhibited improved selectivity profiles.

Collectively, these results indicate that mitochondrial targeting through CL binding is a key determinant of cellular activity within this compound series, rather than PARP1 inhibition alone.

- **Impact on mitochondrial respiration and H_2_O_2_ production rate**

The effects of 1 µM compound **2c** (one of the most cytotoxic) and compound **3g** (the most selective) on mitochondrial respiration and H_2_O_2_ production were assessed using high-resolution respirometry in permeabilized cardiac muscle fibers. Permeabilized fibers were chosen as a model because of minimal tissue requirement, native mitochondrial morphology preservation (no mechanical homogenization), and representation of all mitochondrial subpopulations (no loss during isolation of mitochondria) [18].

**Figure 1:**
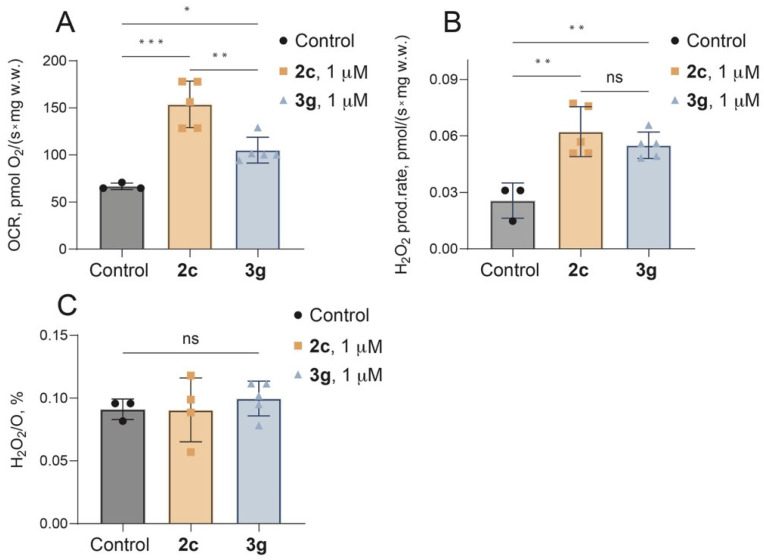
Impact of **2c** and **3g** on mitochondrial respiration and H_2_O_2_ production in cardiac muscle fibers at 1 µM concentration under OXPHOS conditions. (**A**) Oxygen consumption rate (OCR); (**B**) H_2_O_2_ production rate; (**C**) H_2_O_2_/O ratio. Values are shown as mean ± S.D. (n = 3–5 experiments). Statistically significant difference was considered when *p* < 0.05 (* *p* < 0.05, ** *p* < 0.01, *** *p* < 0.001, ns—not significant, one-way ANOVA, followed by Dunnett’s multiple comparisons test).

Both compounds significantly increased oxygen consumption rate (OCR) compared to control, but to different extent. In control fibers, complex I+II-supported OCR was 66.7 ± 3.5 pmol O_2_/s × mg w.w., whereas two-fold increase in OCR was observed in the presence of **2c** (153.8 ± 28.3 pmol O_2_/s × mg w.w). Compound **3g** also significantly increased OCR compared to control (105.2 ± 3.5 pmol O_2_/s × mg w.w), but had lower effect than **2c** (Figure 1A).

These effects were accompanied by a proportional increase in H_2_O_2_ production rate. H_2_O_2_ production increased from a basal level of 0.026 ± 0.009 pmol O_2_/s × mg w.w. to 0.062 ± 0.014 pmol O_2_/s × mg w.w. in the presence of **2c** and to 0.055 ± 0.004 pmol O_2_/s × mg w.w. with **3g** (Figure 1B). Despite these changes, the H_2_O_2_/O ratio remained unchanged (Figure 1C), that means that the elevated ROS generation is driven by accelerated electron flux rather than a specific defect in electron transfer system.

The observed combination of a dramatic increase in OCR accompanied by a proportional H_2_O_2_ release is characteristic for mitochondrial uncouplers. This can be explained by increased permeability and destabilization of the inner mitochondrial membrane causing a proton leakage. Similar effects have been shown for other lipophilic triphenylphosphonium derivatives [19,20,21], including well-known MitoQ [22]. This conclusion is further supported by the difference in the affinity for CL: **2c**, which possesses a higher affinity for CL, acted as a significantly stronger uncoupler than **3g**.

Notably, the excessive uncoupling observed with **2c** explains low selectivity toward cancer cells, leading to generalized cytotoxicity. In contrast, the moderate affinity of **3g** toward inner mitochondrial membrane functionally leads to milder uncoupling and provides better selectivity. These findings align well with established mitochondriotropic pharmacology, where moderate uncouplers can exploit the hyperpolarized mitochondrial membrane of cancer cells to induce selective lethality, while excessive accumulation of uncouplers, driven by high membrane affinity, causes membrane disruption and leads to toxicity in healthy tissue [23,24,25].

## 4. Discussion

The present study demonstrates that phosphonium-conjugated derivatives of PARPi retain potent PARP1 inhibitory activity while gaining new properties through selective CL binding and enhanced cytotoxicity in cancer cells. By combining mitochondrial targeting with PARP inhibition, these compounds offer a mechanistically distinct therapeutic approach that could complement conventional nuclear-directed PARP inhibitors.

All of synthesized olaparib-based conjugates exhibited low nanomolar PARP1 inhibitory activity, confirming that a trialkyl(aryl)phosphonium moiety does not compromise the molecules’ ability to bind PARP1. These findings align with prior structure–activity studies showing that olaparib tolerates modifications at peripheral regions without a major loss of potency [26,27]. In contrast, with rucaparib, which has been less frequently used as a modification scaffold, we found that introduction of bulky lipophilic substituents at its secondary amino group must be approached with caution—such modifications significantly reduce inhibitory potency. Structural insights from published PARP1 co-crystal structures provide a plausible explanation for the different tolerance of olaparib and rucaparib scaffolds toward peripheral modification [28]. Although both inhibitors occupy the NAD^+^ binding pocket, their binding geometries differ substantially. Rucaparib is compactly located in the binding pocket, and its secondary amino group forms an additional hydrogen bond with the HD-subdomain residue Glu763, resulting in limited tolerance for bulky substituents. This interaction appears to be very important for the tight binding, making rucaparib less tolerant to bulky substituents and leading to a pronounced loss of inhibitory potency upon modification. In contrast, olaparib represents a more flexible scaffold, with its piperazine moiety oriented toward a more solvent-exposed region of the pocket, allowing modification with bulky substituents without significantly perturbing the core interactions required for PARP1 inhibition.

A major distinguishing feature of the conjugated compounds is their ability to interact with CL, a phospholipid almost exclusively localized in the inner mitochondrial membrane. This property provides a mechanistic basis for their mitochondrial targeting, driven by lipophilic cation-mediated accumulation. Among the tested series, olaparib derivative **2d** and conjugates with rucaparib **5a** and **6c** displayed the highest cardiolipin affinity, with EC_50_ values of 6.77–12.99 µM. By contrast, acylated derivatives of olaparib **3a**–**e** exhibited negligible cardiolipin binding (EC_50_ > 400 µM). This can be attributed to the fact that a polar carbonyl group increases local hydrophilicity and partially restricts conformational flexibility, compared to a fully saturated alkyl chain, which can impair the optimal orientation required for efficient uptake of lipophilic cations. This observation highlights that even subtle structural modifications can have a pronounced impact on mitochondrial targeting potential.

Compounds with high affinity for CL displayed much higher cytotoxicity, far exceeding that of unmodified olaparib and rucaparib. This pattern suggests that mitochondrial accumulation amplifies cytotoxic effects, likely through a combination of mitochondrial stress and inhibition of PARP activity. This observation is consistent with recent findings that mitochondrial PARP1 contributes to mtDNA regulation, respiratory control, and stress adaptation; therefore, inhibition of this pool may potentially disrupt mitochondrial bioenergetics and promote apoptotic signaling [29,30]. We also confirmed that in this type of conjugates, lipophilic triphenylphosphonium moiety not only acts as a targeting moiety, but can also have a functional impact on mitochondrial respiration by uncoupling electron transfer system and increasing H_2_O_2_ production.

However, the higher efficacy of strong CL binders was accompanied by higher uncoupling potency that resulted in reduced selectivity toward cancer cells, highlighting a trade-off between efficacy and safety. In contrast, moderate CL binders retained substantial cytotoxic activity while exhibiting improved selectivity. These findings indicate that fine-tuning cardiolipin affinity, and thus mitochondrial accumulation, by careful selection of the targeting warhead offers a strategy to balance potency and safety.

These findings also raise important mechanistic questions for future investigation. Although the new conjugates preserve high PARP1 inhibitory potency, the relative contribution of PARP1 inhibition versus CL binding-induced membrane perturbation remains to be discovered. Furthermore, mitochondria-targeted PARP inhibitors may exhibit therapeutic advantages when combined with agents that induce mitochondrial apoptosis, such as temozolomide, [31] mtDNA-damaging chemotherapeutics, e.g., cisplatin or anthracyclines, or oxidative stress inducers [32], which could synergize through complementary activation of mitochondrial apoptosis pathways.

## 5. Conclusions

In summary, this study shows that phosphonium-conjugated derivatives of olaparib and rucaparib retain strong PARP1 inhibitory activity while gaining high affinity for CL and enhanced cytotoxicity in BRCA1-deficient cancer cells. The observed link between PARP inhibition, CL binding, and cytotoxic activity supports the emerging concept of mitochondria-targeted PARP inhibitors. Redirecting these classical, nuclear-acting PARP inhibitors toward mitochondria introduces a distinct mechanism that may improve therapeutic efficacy and help overcome resistance associated with current PARP-based treatments. Among the tested compounds, **3g** and **5a** stand out as promising lead structures for the further development of next-generation PARP inhibitors designed to exploit mitochondrial vulnerabilities and broaden the therapeutic potential of this drug class.

## Figures and Tables

**Table 1:** In vitro PARP1 inhibition, affinity for CL, and cytotoxicity of **2a**–**d, 3a**–**g, 5**, and **6a**–**c**.

			Cytotoxicity, µM	
	PARP, IC_50_, nM	CL Binding EC_50_, µM *	HCC1937	H9C2
**2a**	17.0 ± 1.91	579.7 ± 13.07	>10	>100
**2b**	8.20 ± 0.14	234.03 ± 4.02	>10	>100
**2c**	14.11 ± 3.53	56.65 ± 1.67	1.25 ± 0.43	3.15 ± 0.28
**2d**	10.3 ± 0.22	12.99 ± 1.75	0.93 ± 0.25	1.05 ± 0.24
**3a**	3.43 ± 0.04	>500	>10	>100
**3b**	3.90 ± 0.44	>500	>10	>100
**3c**	7.71 ± 0.78	>500	>10	>100
**3d**	6.23 ± 0.38	421.2 ± 36.62	>10	75 ± 11
**3e**	6.14 ± 0.76	521 ± 17.54	>10	>100
**3f**	3.91 ± 0.37	445.43 ± 19.63	>10	>100
**3g**	4.73 ± 0.24	125.03 ± 2.21	4.67 ± 0.61	15.03 ± 3.49
**5a**	13.20 ± 1.79	11.85 ± 0.85	18.59 ± 2.52	69.38 ± 2.39
**6a**	20.29 ± 3.61	>500	>100	>100
**6b**	87.84 ± 7.01	25.07 ± 0.54	26.06 ± 4.6	89.57 ± 11.47
**6c**	>100	6.77 ± 0.09	2.01 ± 0.37	5.37 ± 1.42
Rucaparib	1.93 ± 0.29	>500	32.24 ± 2.21	35.01 ± 0.67
Olaparib	3.10 ± 0.21	>500	>100	>100

* EC_50_ of *cytochrome* *c* is 0.29 ± 0.05 µM.

## Data Availability

Supplementary Materials contain copies of ^1^H, ^13^C, ^19^F, and ^31^P NMR and HRMS spectra. More data are available upon polite request.

## Supplementary Materials

The following supporting information can be downloaded at: https://www.mdpi.com/article/10.3390/biom16010165/s1. Copies of HRMS spectra of all final compounds are presented on pages S2–S5. Copies of ^1^H, ^13^C, ^19^F, ^31^P NMR spectra are presented on pages S6–S64.
