# Supplementary material for: Development of Mitochondria-Targeted PARP Inhibitors
Source: Biomolecules. 2026 Jan 19;16(1):165. doi: 10.3390/biom16010165 (PMC12839213; doi:10.3390/biom16010165)
Supplement: Supplementary file 1 [file biomolecules-16-00165-s001.zip › biomolecules-3984470-supplementary.pdf]

Supplementary Materials

HRMS spectra of **2a**

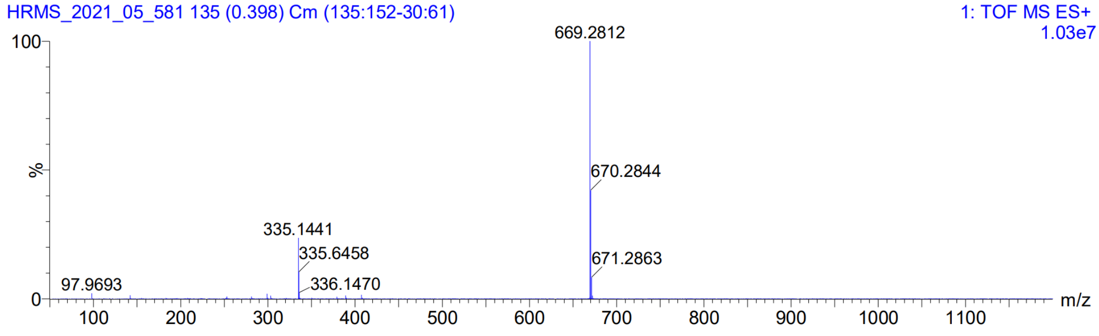

HRMS spectra of **2b**

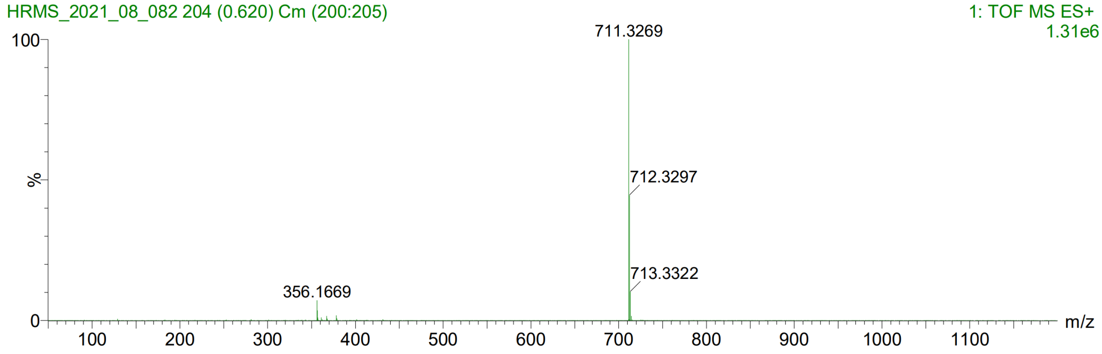

HRMS spectra of **2c**

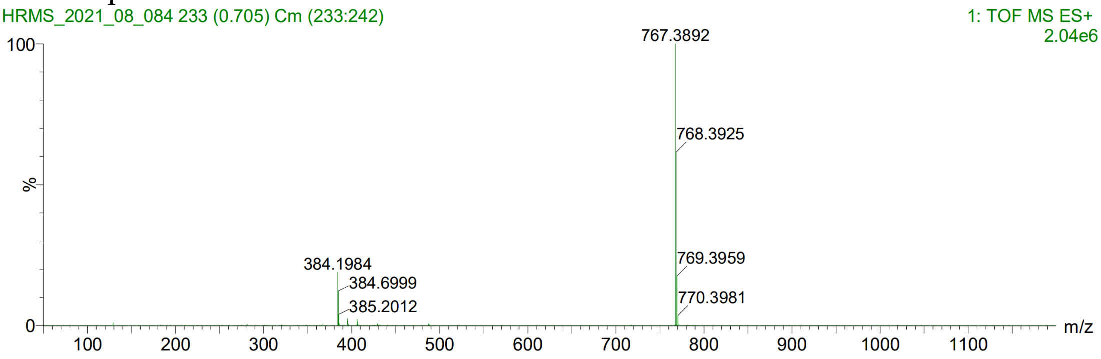

HRMS spectra of **2d**

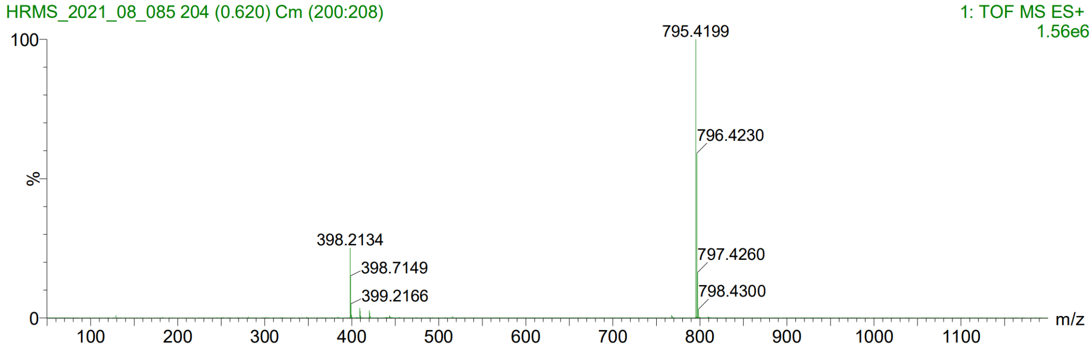

HRMS spectra of **3a**

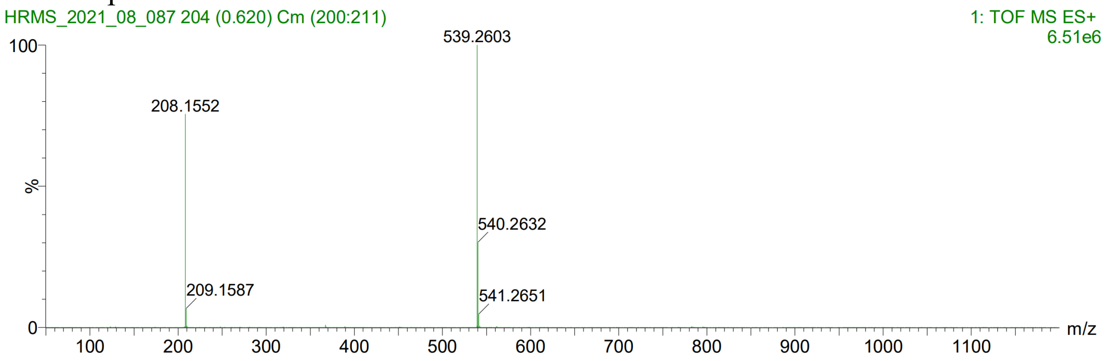

HRMS spectra of **3b**

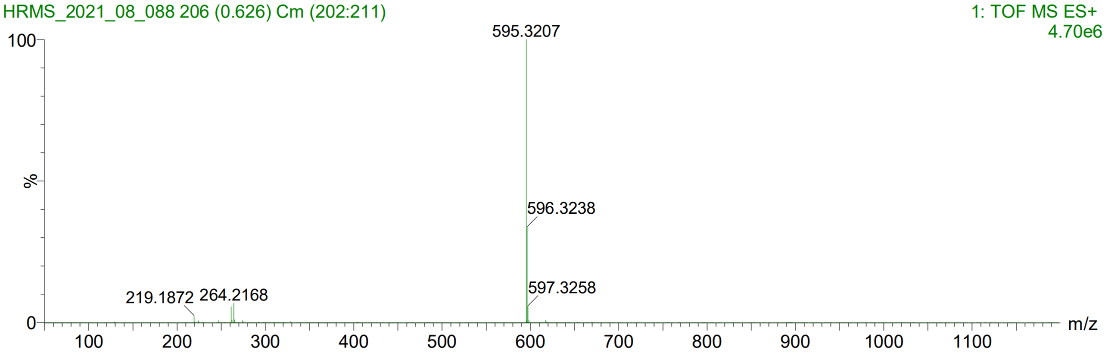

HRMS spectra of **3c**

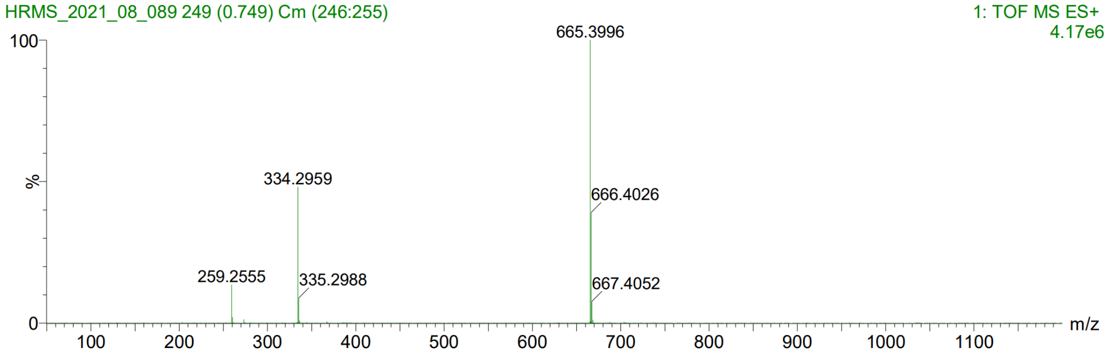

HRMS spectra of **3d**

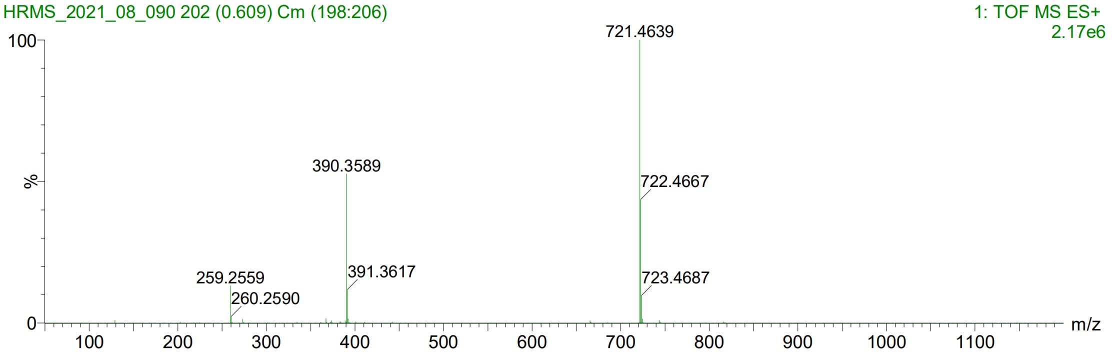

HRMS spectra of **3e**

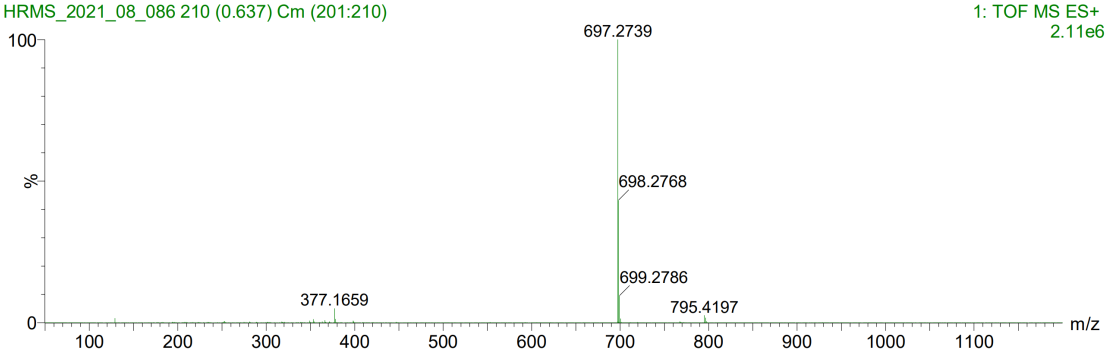

HRMS spectra of **3f**

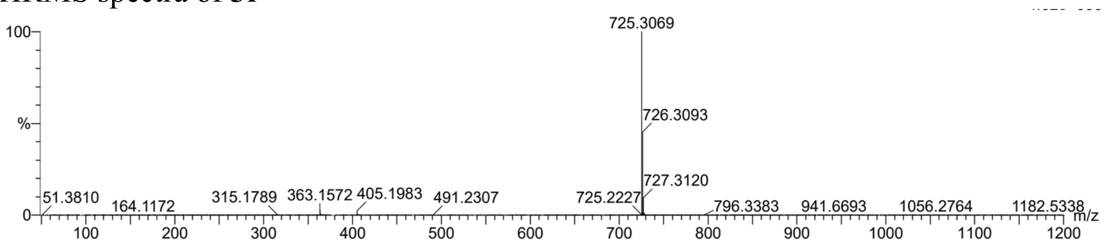

HRMS spectra of **3g**

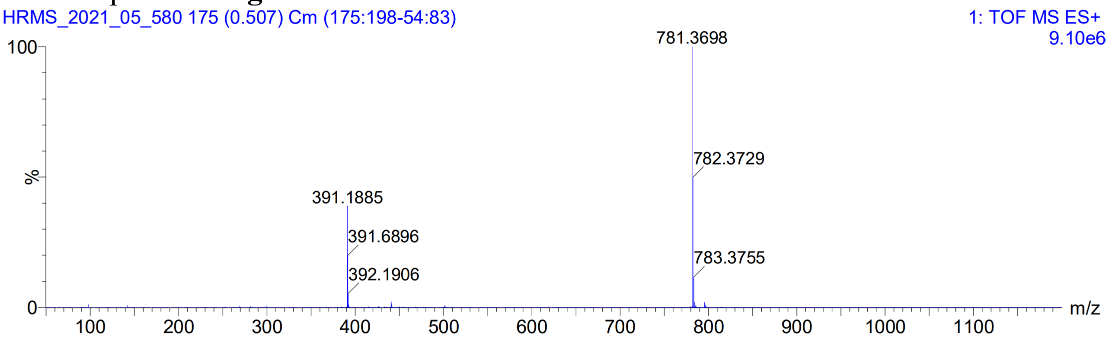

HRMS spectra of **5a**

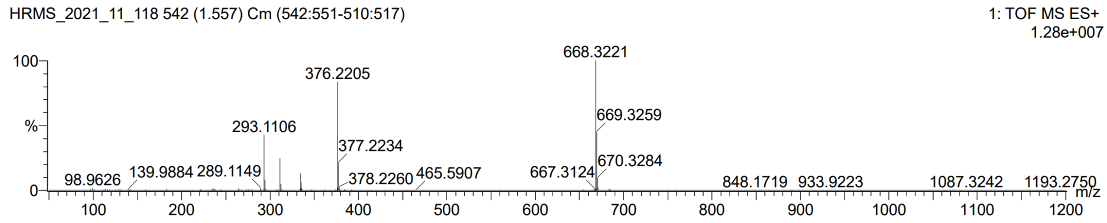

HRMS spectra of **6a**

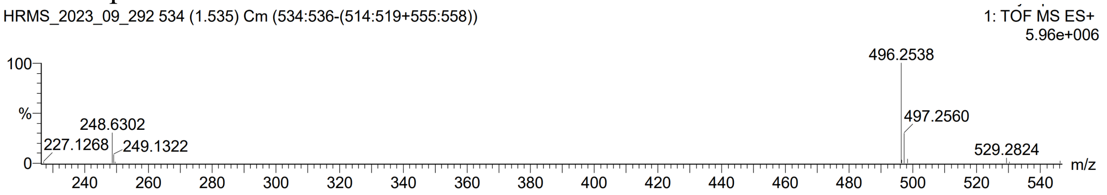

HRMS spectra of **6b**

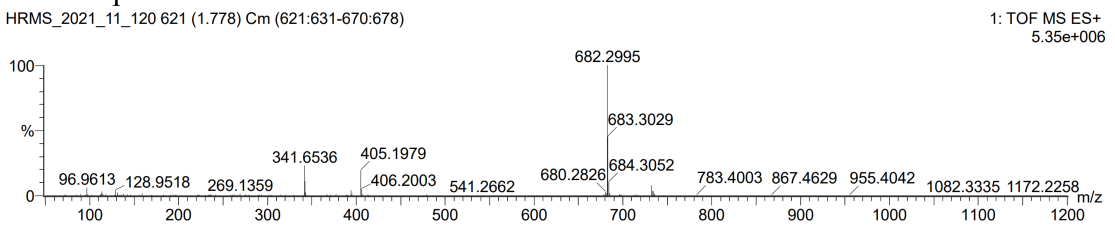

HRMS spectra of **6c**

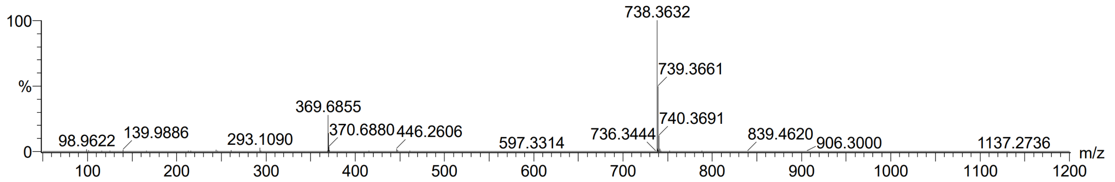

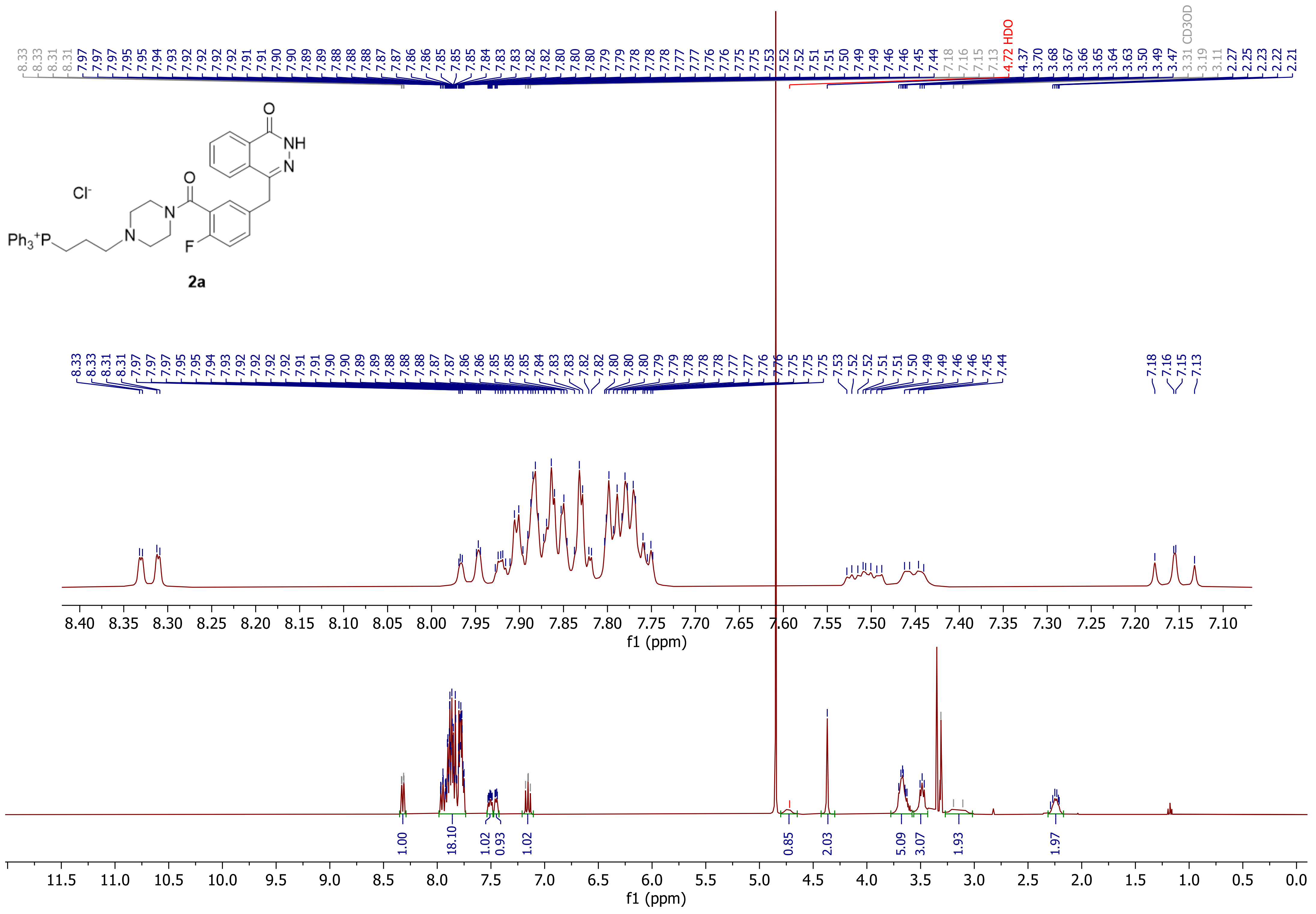

8.33 8.33 8.31 8.31 7.97 7.97 7.97 7.95 7.95 7.94 7.93 7.92 7.92 7.92 7.92 7.91 7.91 7.90 7.90 7.89 7.89 7.88 7.88 7.88 7.87 7.87 7.86 7.85 7.85 7.85 7.84 7.83 7.83 7.82 7.82 7.80 7.80 7.80 7.79 7.79 7.78 7.78 7.78 7.77 7.77 7.76 7.76 7.75 7.75 7.53 7.52 7.52 7.51 7.51 7.50 7.49 7.49 7.46 7.46 7.45 7.44 7.18 7.16 7.15 7.13 4.72 HDO 4.37 3.70 3.68 3.67 3.66 3.65 3.64 3.63 3.50 3.49 3.47 3.31 CD3OD 3.19 3.11 2.27 2.25 2.23 2.22 2.21
O
NH
N
O
Cl⁻
N
N
Ph3+P
F
2a
f1 (ppm)
1.00 18.10 1.02 0.93 1.02 0.85 2.03 5.09 3.07 1.93 1.97
f1 (ppm)

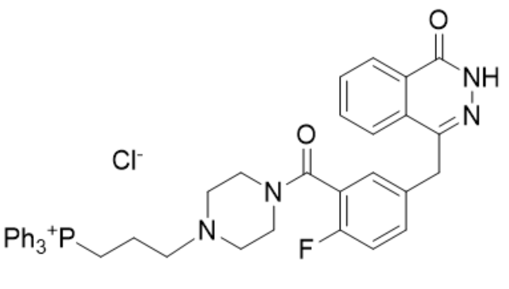
O
NH
N
O
Cl-
N
Ph3+P
N
F

2a

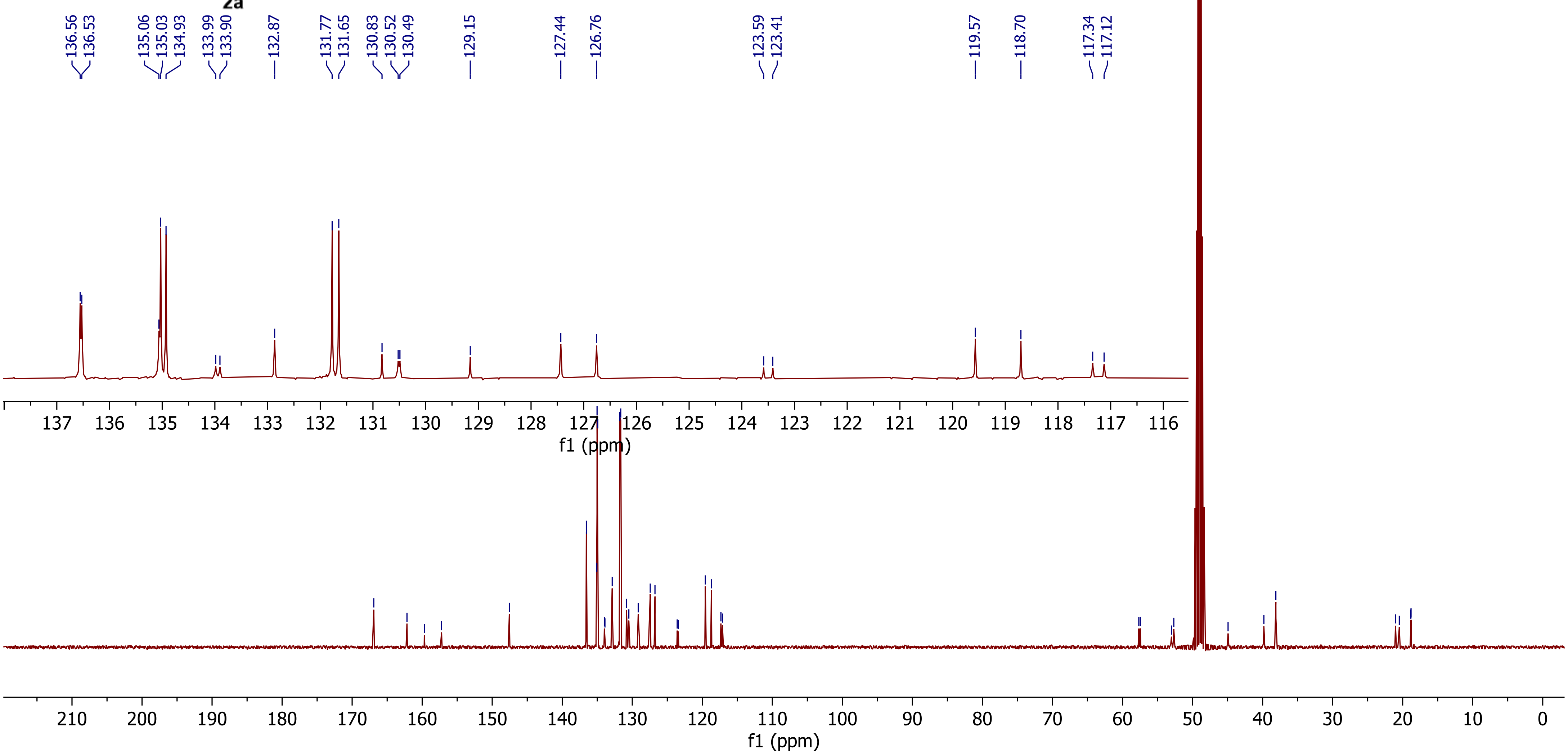
166.91
162.17
159.69
157.23
147.56
136.56
136.53
135.06
135.03
134.93
133.99
133.90
132.87
131.77
131.65
130.83
130.52
130.49
129.15
127.44
126.76
123.59
123.41
119.57
118.70
117.34
117.12
57.67
57.45
53.01
52.68
49.00 CD3OD
44.93
39.81
38.10
21.02
20.48
18.83
18.80
137 136 135 134 133 132 131 130 129 128 127 126 125 124 123 122 121 120 119 118 117 116
f1 (ppm)
210 200 190 180 170 160 150 140 130 120 110 100 90 80 70 60 50 40 30 20 10 0
f1 (ppm)

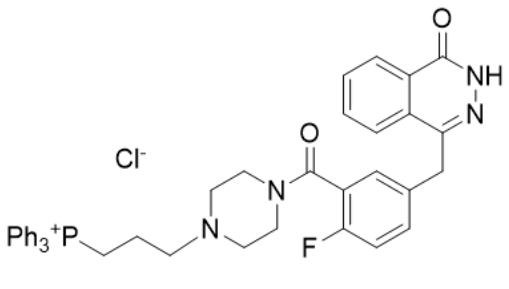

Cl-
Ph3+P
O
NH
N
N
F

2a

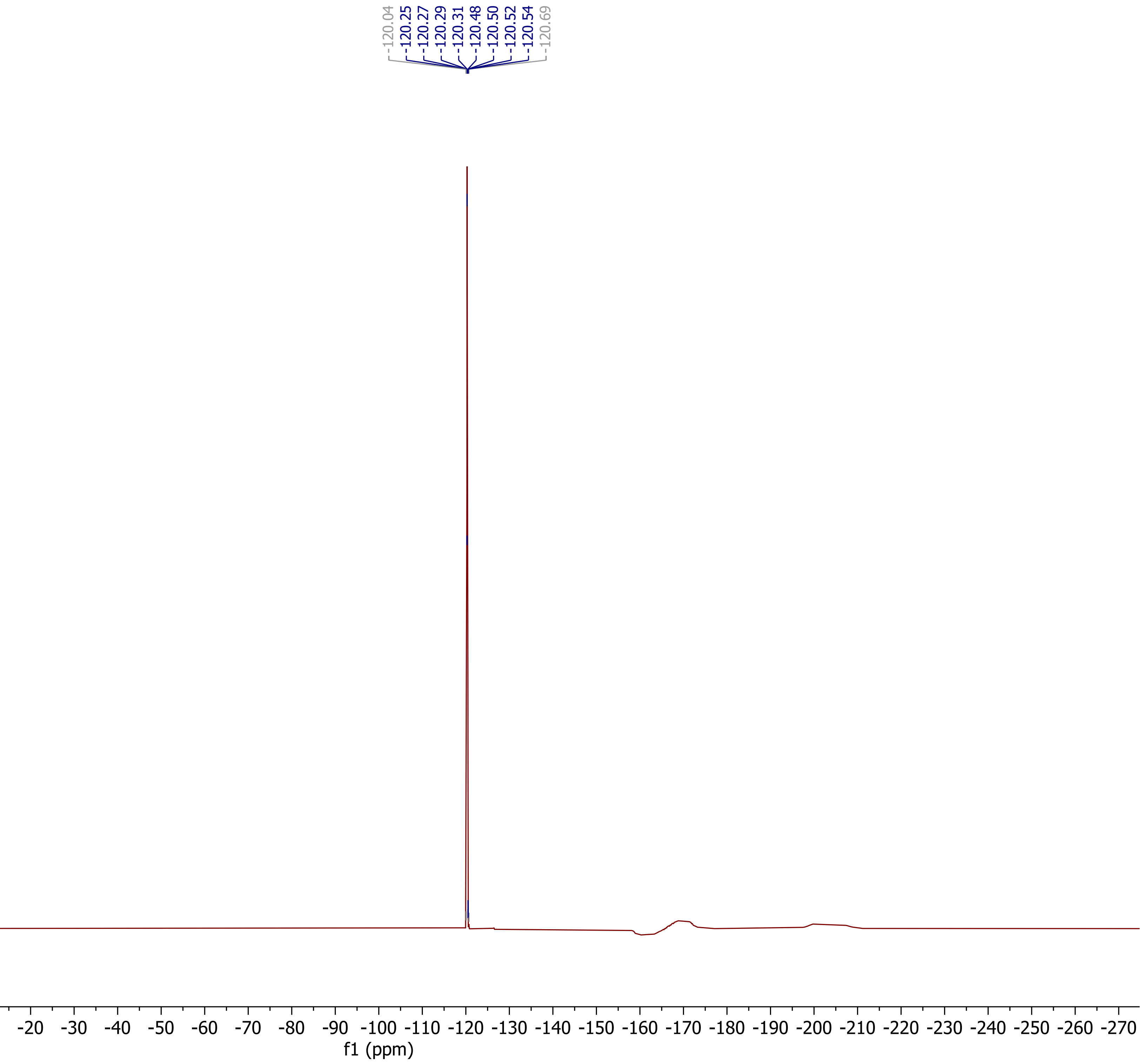

-120.04
-120.25
-120.27
-120.29
-120.31
-120.48
-120.50
-120.52
-120.54
-120.69
70 60 50 40 30 20 10 0 -10 -20 -30 -40 -50 -60 -70 -80 -90 -100 -110 -120 -130 -140 -150 -160 -170 -180 -190 -200 -210 -220 -230 -240 -250 -260 -270
f1 (ppm)

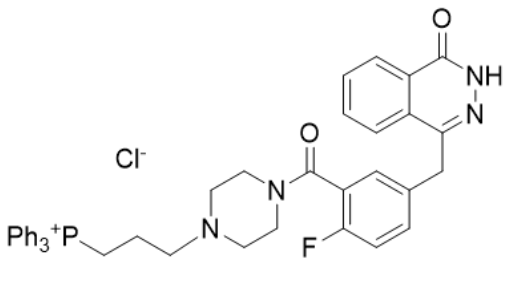

O
NH
N
O
Cl⁻
N
N
Ph3+P
F

2a

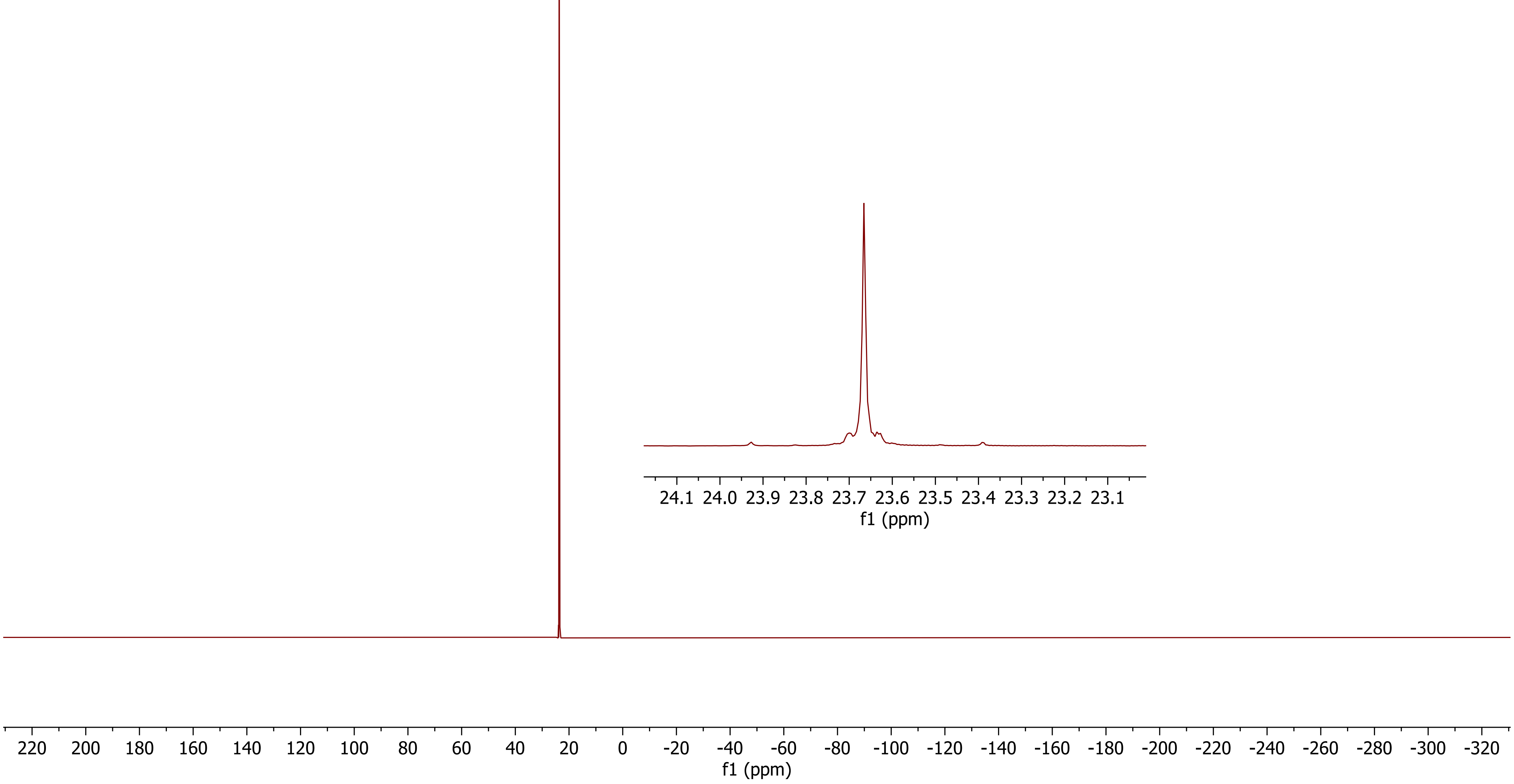

23.67
24.1 24.0 23.9 23.8 23.7 23.6 23.5 23.4 23.3 23.2 23.1
f1 (ppm)
220 200 180 160 140 120 100 80 60 40 20 0 -20 -40 -60 -80 -100 -120 -140 -160 -180 -200 -220 -240 -260 -280 -300 -320
f1 (ppm)

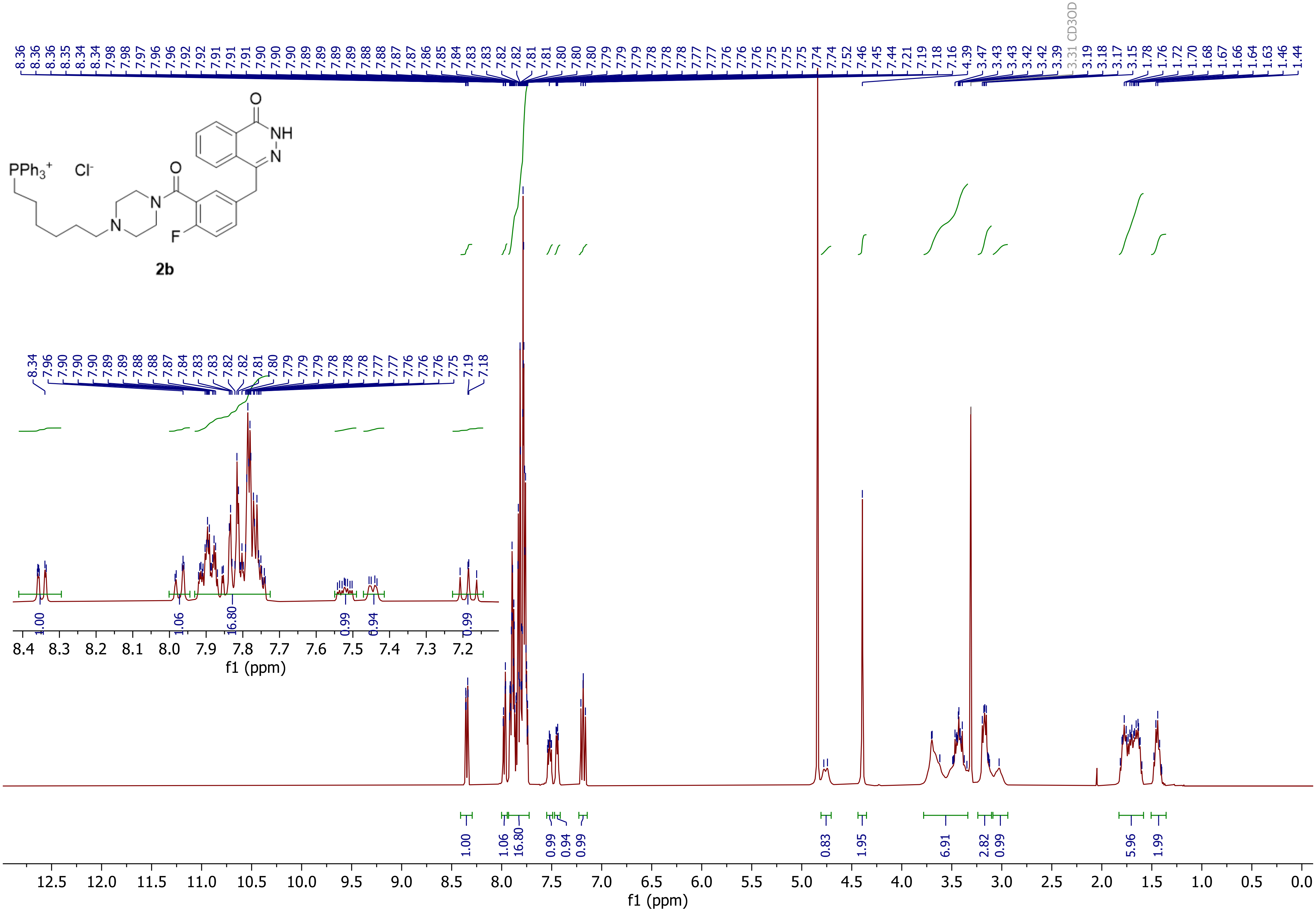

8.36 8.36 8.36 8.35 8.34 8.34 7.98 7.98 7.97 7.96 7.96 7.92 7.92 7.91 7.91 7.91 7.90 7.90 7.89 7.89 7.89 7.89 7.88 7.88 7.87 7.87 7.86 7.85 7.84 7.83 7.83 7.82 7.82 7.81 7.81 7.80 7.80 7.80 7.79 7.79 7.78 7.78 7.78 7.77 7.77 7.76 7.76 7.76 7.75 7.75 7.74 7.74 7.52 7.46 7.45 7.44 7.21 7.19 7.18 7.16 4.39 3.47 3.43 3.43 3.42 3.42 3.39 3.31 CD3OD 3.19 3.18 3.17 3.15 1.78 1.76 1.72 1.70 1.68 1.67 1.66 1.64 1.63 1.46 1.44
O
NH
N
PPh3+ Cl-
O
N
N
F
2b
8.34 7.96 7.90 7.90 7.89 7.89 7.88 7.88 7.87 7.84 7.83 7.83 7.82 7.82 7.81 7.80 7.79 7.79 7.79 7.78 7.78 7.78 7.77 7.77 7.76 7.76 7.75 7.19 7.18
1.00 1.06 16.80 0.99 0.94 0.99
8.4 8.3 8.2 8.1 8.0 7.9 7.8 7.7 7.6 7.5 7.4 7.3 7.2
f1 (ppm)
1.00 1.06 16.80 0.99 0.94 0.99 0.83 1.95 6.91 2.82 0.99 5.96 1.99
12.5 12.0 11.5 11.0 10.5 10.0 9.5 9.0 8.5 8.0 7.5 7.0 6.5 6.0 5.5 5.0 4.5 4.0 3.5 3.0 2.5 2.0 1.5 1.0 0.5 0.0
f1 (ppm)

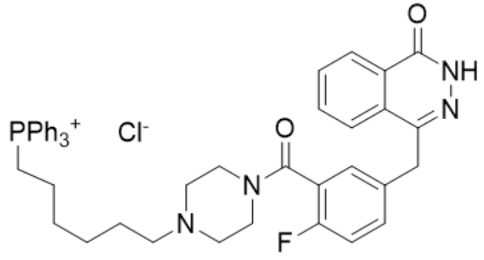

PPh3+
Cl-
O
NH
N
N
O
F

2b

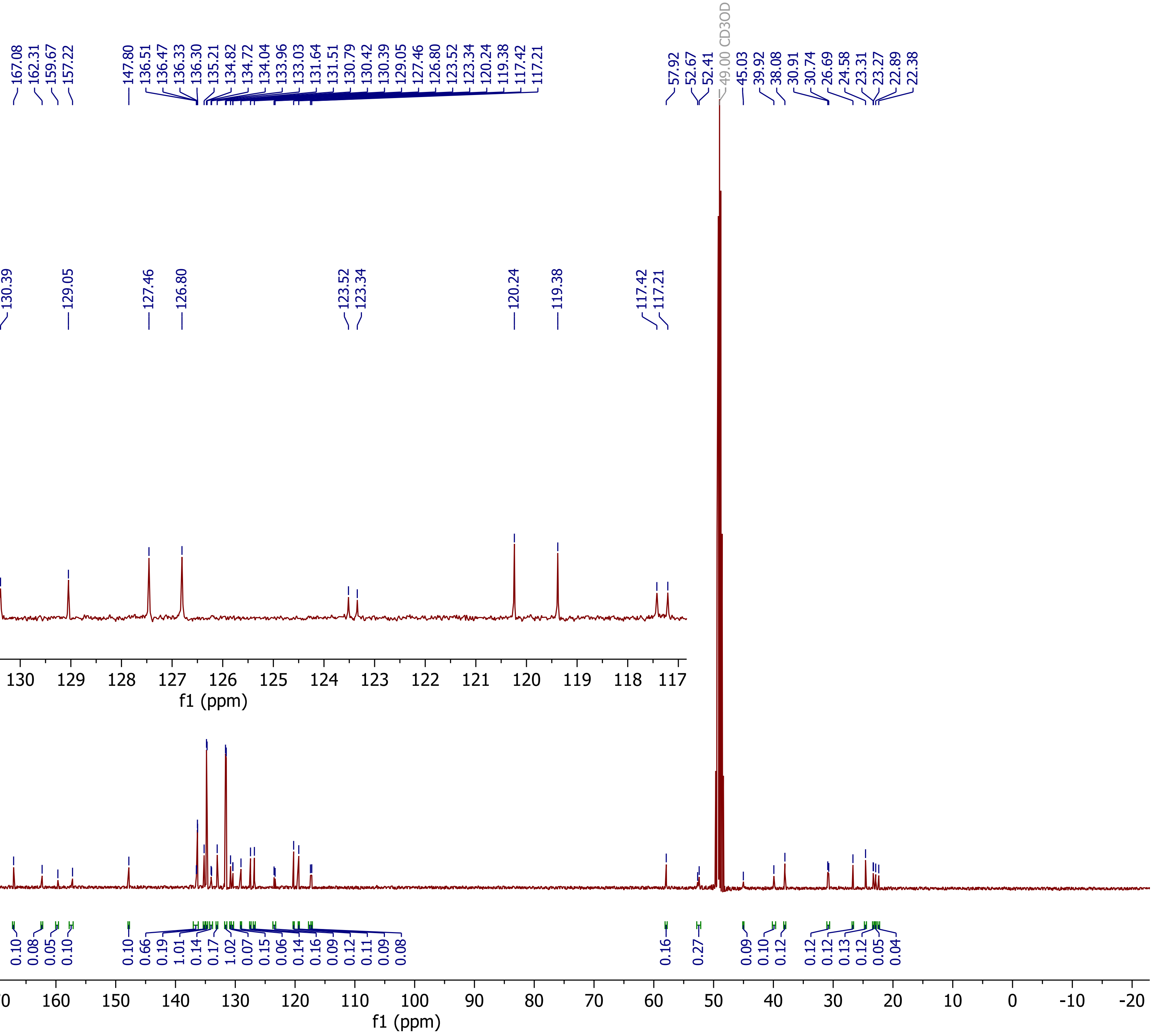

167.08
162.31
159.67
157.22
147.80
136.51
136.47
136.33
136.30
135.21
134.82
134.72
134.04
133.96
133.03
131.64
131.51
130.79
130.42
130.39
129.05
127.46
126.80
123.52
123.34
120.24
119.38
117.42
117.21
57.92
52.67
52.41
49.00 CD3OD
45.03
39.92
38.08
30.91
30.74
26.69
24.58
23.31
23.27
22.89
22.38
0.10
0.08
0.05
0.10
0.10
0.66
0.19
1.01
0.14
0.17
1.02
0.07
0.15
0.06
0.14
0.16
0.09
0.12
0.11
0.09
0.08
0.16
0.27
0.09
0.10
0.12
0.12
0.12
0.13
0.12
0.05
0.04
f1 (ppm)
f1 (ppm)

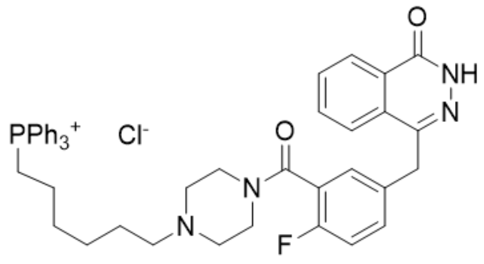

2b

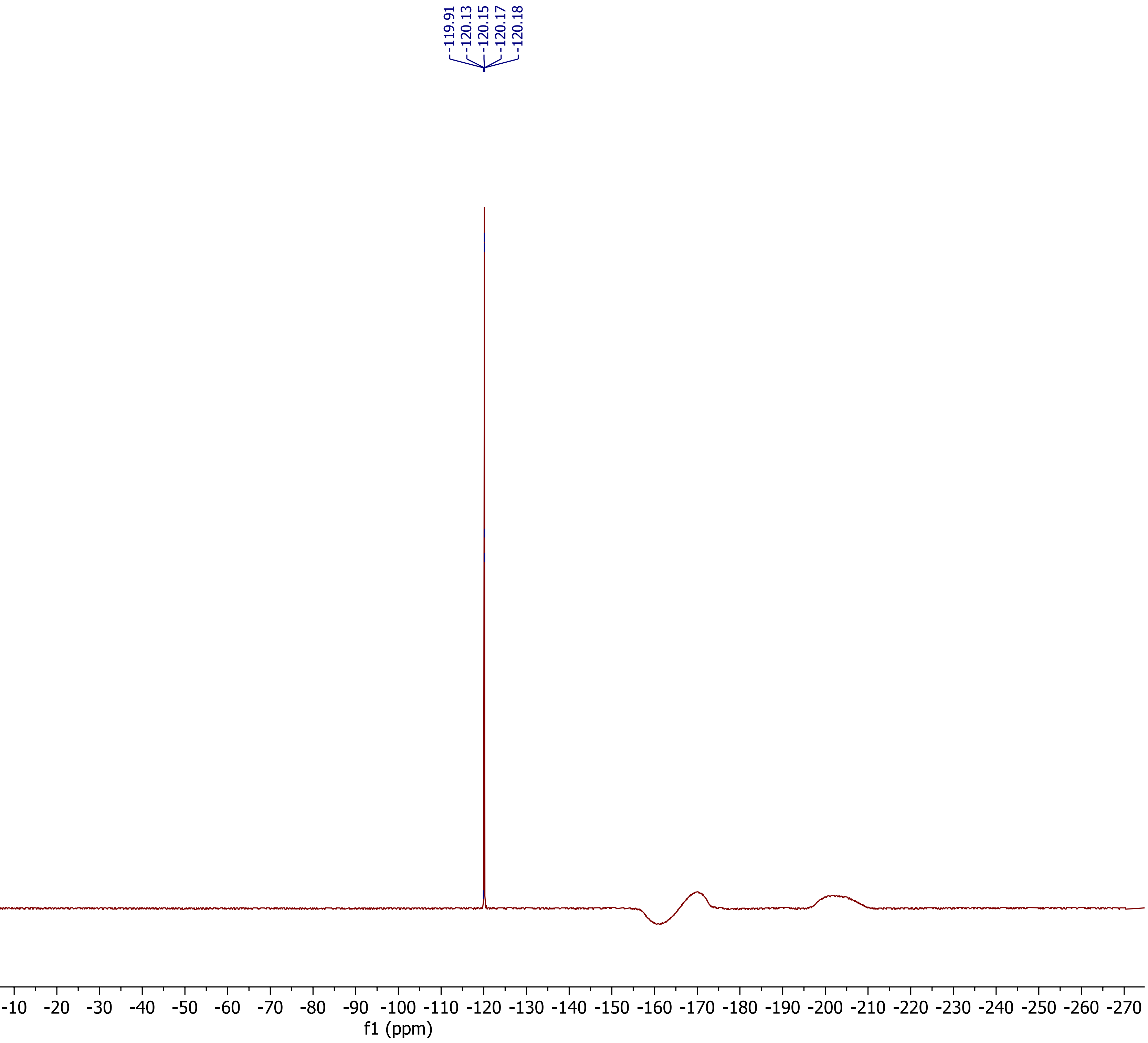

-119.91
-120.13
-120.15
-120.17
-120.18
70 60 50 40 30 20 10 0 -10 -20 -30 -40 -50 -60 -70 -80 -90 -100 -110 -120 -130 -140 -150 -160 -170 -180 -190 -200 -210 -220 -230 -240 -250 -260 -270
f1 (ppm)

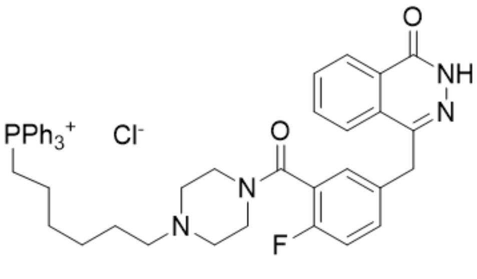

PPh3+
Cl-
O
NH
N
N
F

2b

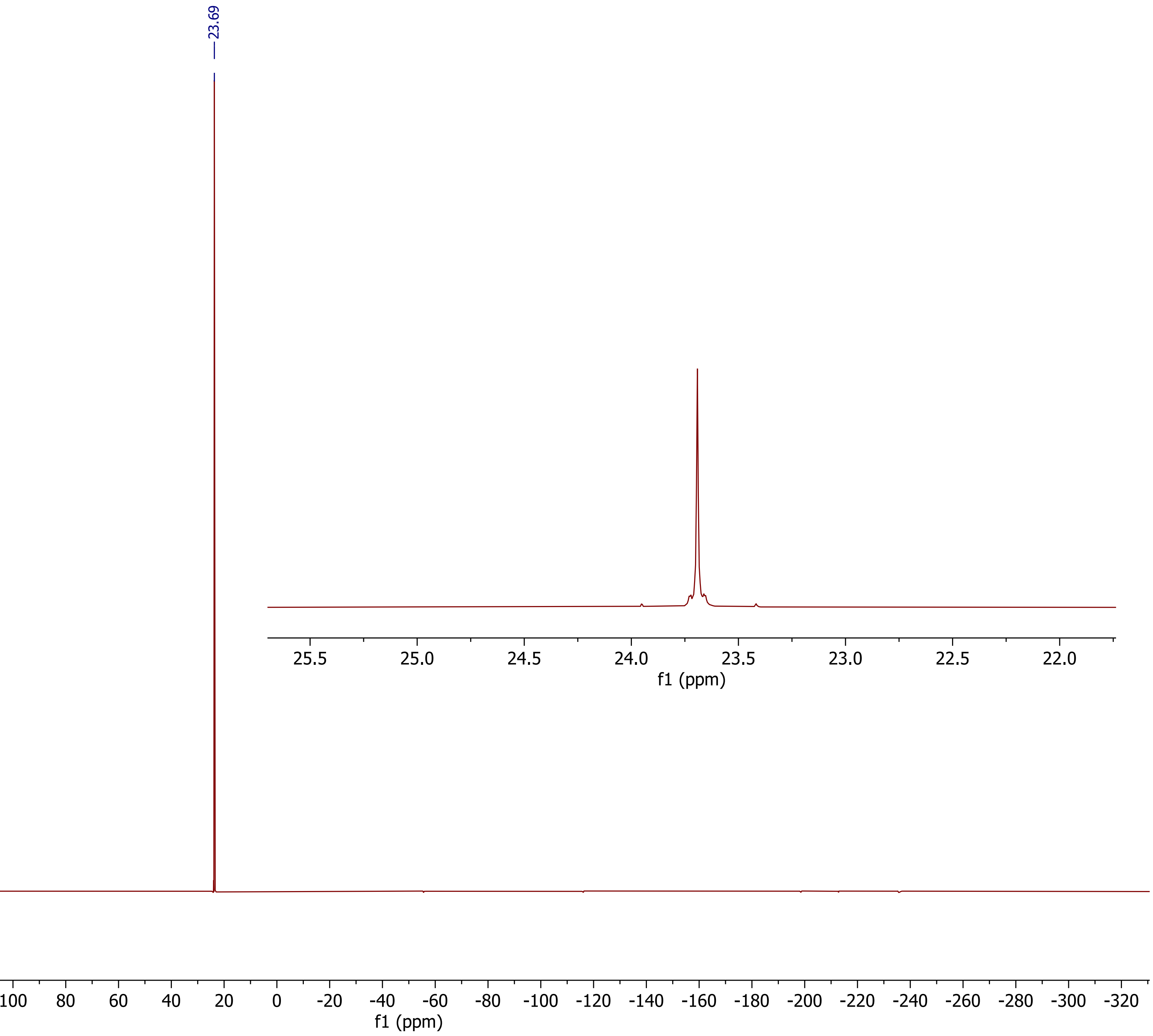

23.69
25.5
25.0
24.5
24.0
23.5
23.0
22.5
22.0
f1 (ppm)
220 200 180 160 140 120 100 80 60 40 20 0 -20 -40 -60 -80 -100 -120 -140 -160 -180 -200 -220 -240 -260 -280 -300 -320
f1 (ppm)

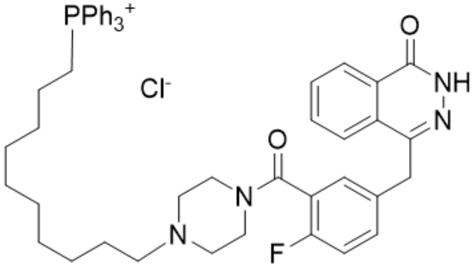

PPh3+
Cl-
O
NH
N
O
N
N
F

2c

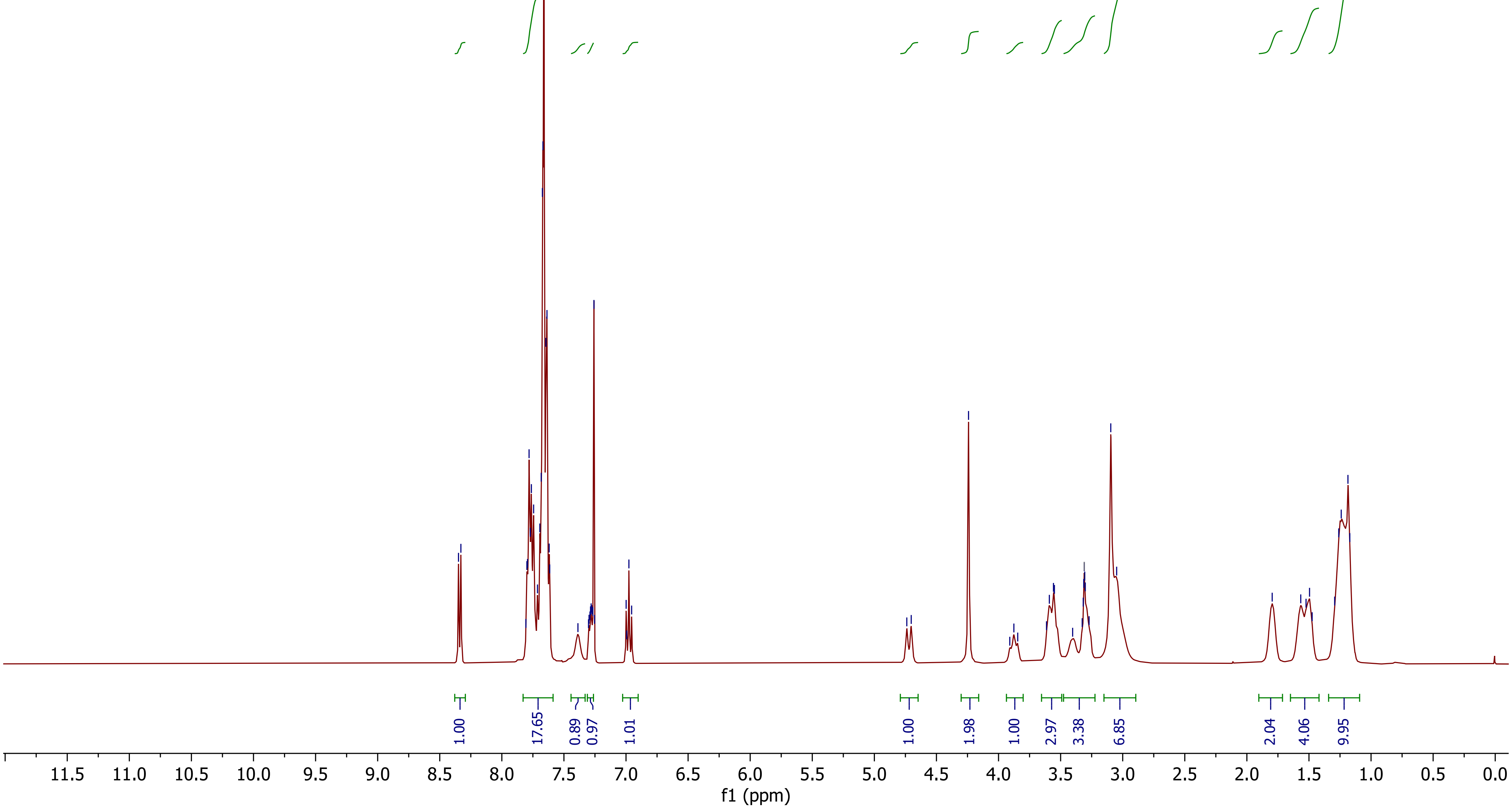

8.35 8.33 7.81 7.80 7.79 7.78 7.77 7.76 7.74 7.71 7.69 7.68 7.67 7.67 7.66 7.64 7.64 7.62 7.61 7.39 7.30 7.30 7.29 7.29 7.28 7.28 7.27 7.26 7.25 7.00 6.99 6.98 6.95 4.74 4.70 4.24 3.91 3.88 3.85 3.61 3.59 3.56 3.55 3.40 3.33 3.32 3.31 3.31 3.31 3.30 3.27 3.10 3.05 1.80 1.57 1.52 1.50 1.47 1.29 1.26 1.24 1.19 1.17
CD3OD
1.00
17.65
0.89
0.97
1.01
1.00
1.98
1.00
2.97
3.38
6.85
2.04
4.06
9.95
11.5 11.0 10.5 10.0 9.5 9.0 8.5 8.0 7.5 7.0 6.5 6.0 5.5 5.0 4.5 4.0 3.5 3.0 2.5 2.0 1.5 1.0 0.5 0.0
f1 (ppm)

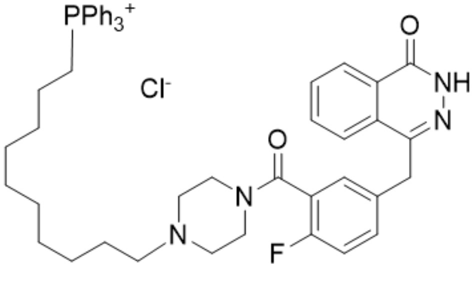

PPh3+
Cl-
O
NH
N
O
N
N
F

2c

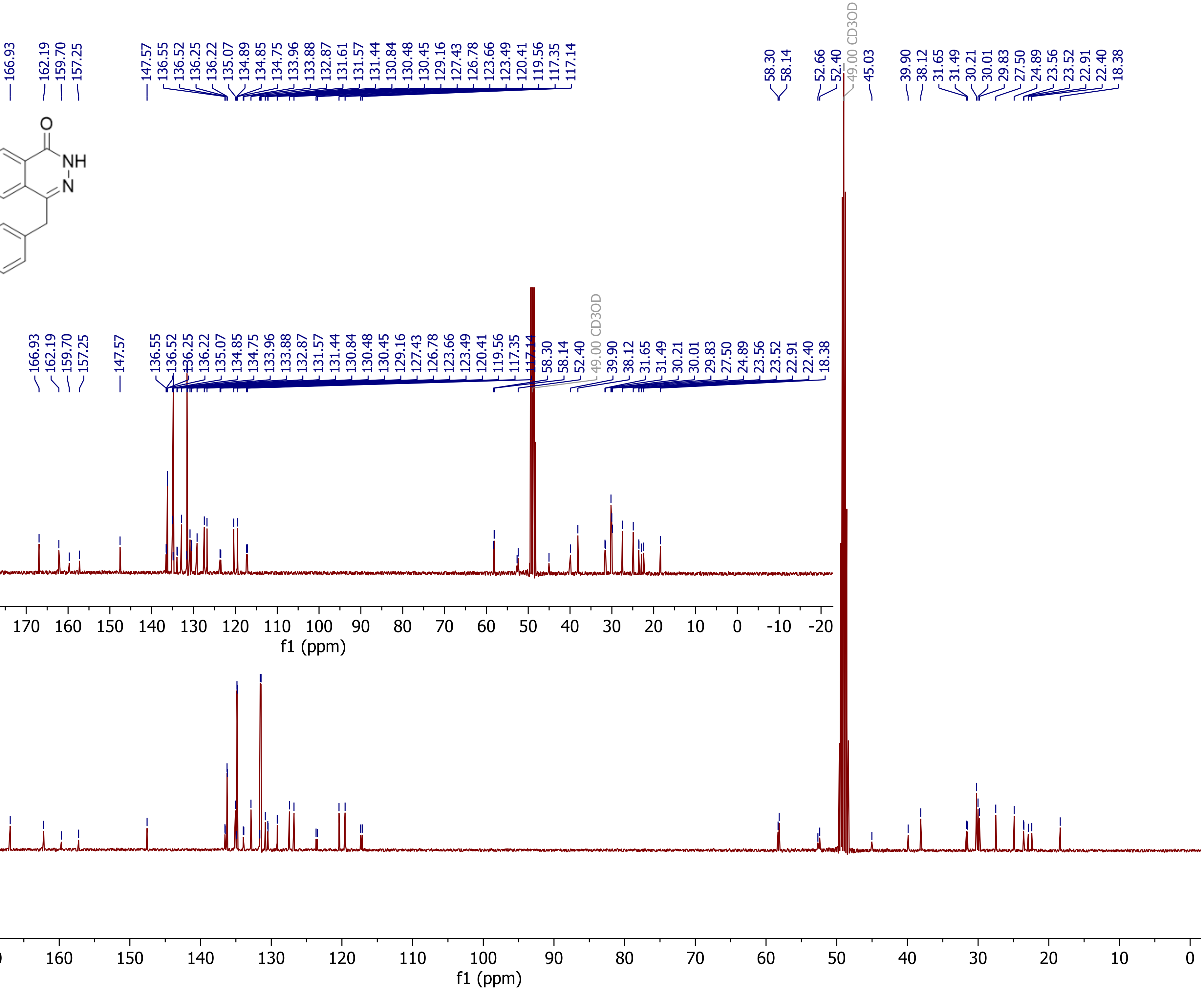

166.93
162.19
159.70
157.25
147.57
136.55
136.52
136.25
136.22
135.07
134.89
134.85
134.75
133.96
133.88
132.87
131.61
131.57
131.44
130.84
130.48
130.45
129.16
127.43
126.78
123.66
123.49
120.41
119.56
117.35
117.14
58.30
58.14
52.66
52.40
49.00 CD3OD
45.03
39.90
38.12
31.65
31.49
30.21
30.01
29.83
27.50
24.89
23.56
23.52
22.91
22.40
18.38
f1 (ppm)
f1 (ppm)

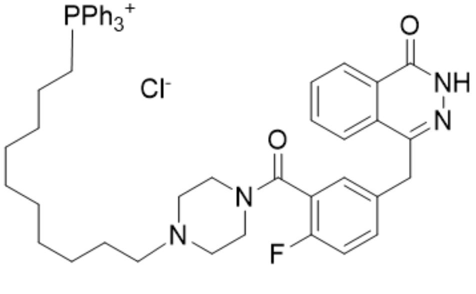
PPh3+
Cl-
O
NH
N
O
N
N
F

2c

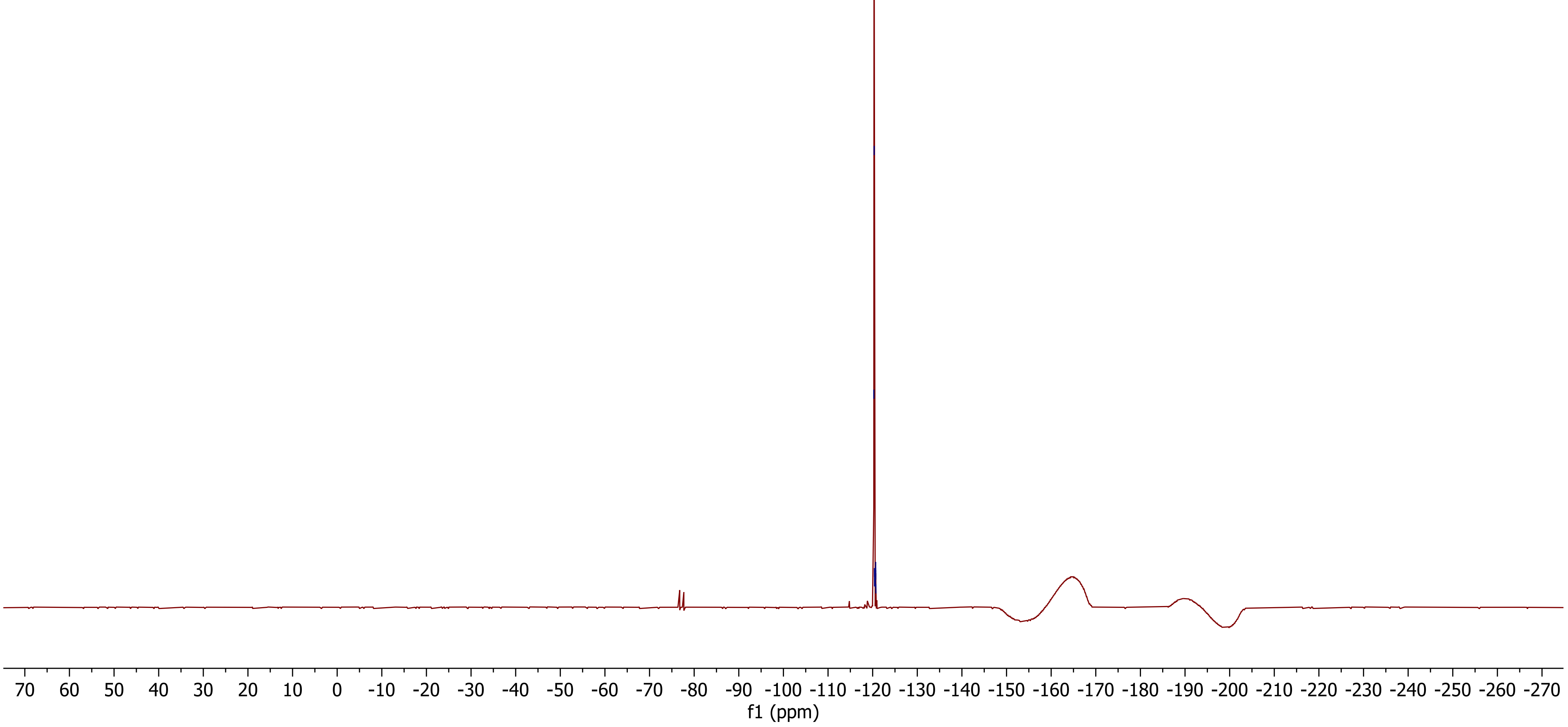
-120.27
-120.30
-120.32
-120.33
-120.41
-120.42
-120.63
-120.65
-120.67
-120.69
-120.71
70 60 50 40 30 20 10 0 -10 -20 -30 -40 -50 -60 -70 -80 -90 -100 -110 -120 -130 -140 -150 -160 -170 -180 -190 -200 -210 -220 -230 -240 -250 -260 -270
f1 (ppm)

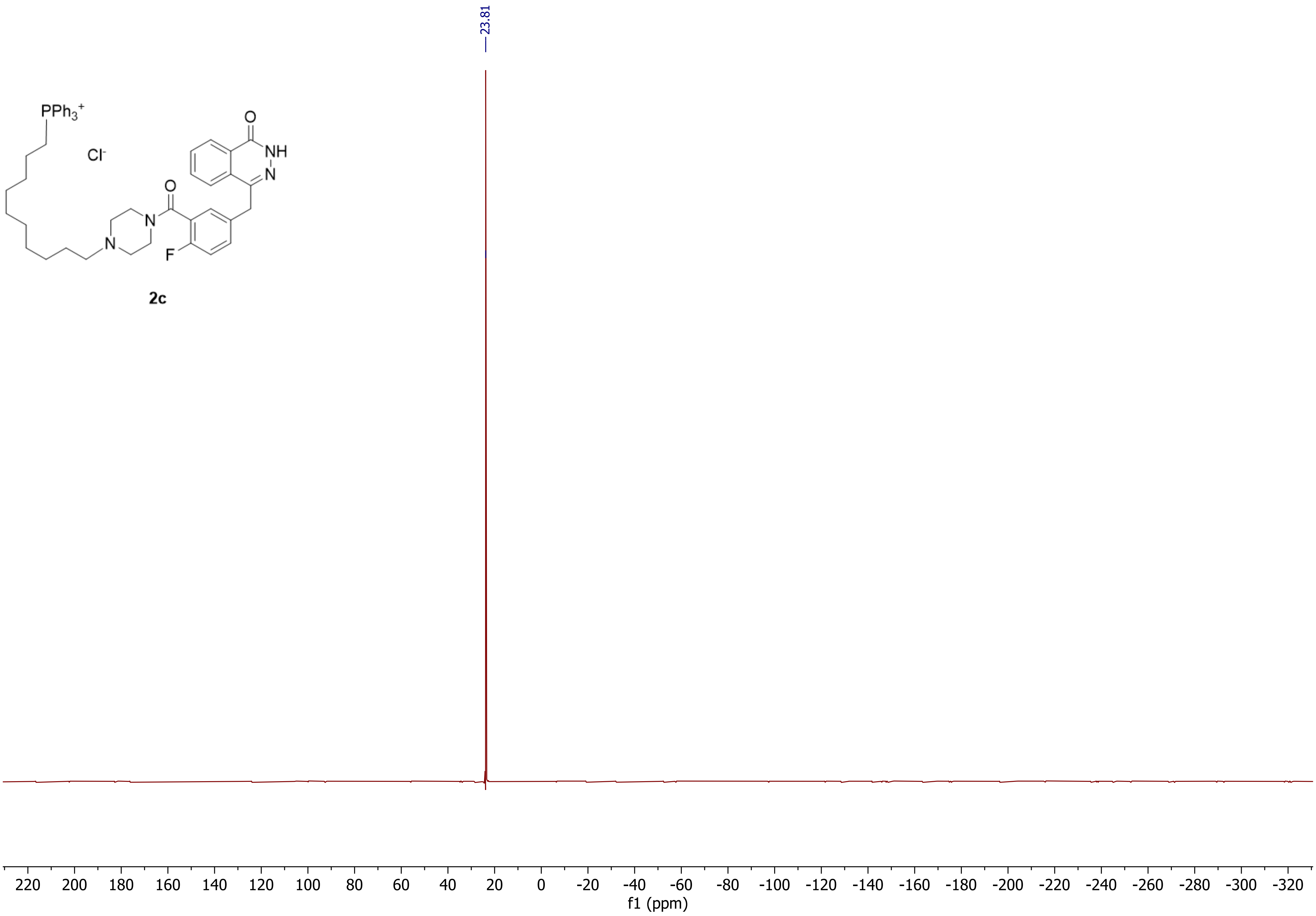
PPh3+
Cl-
O
NH
N
O
N
N
F
23.81
220 200 180 160 140 120 100 80 60 40 20 0 -20 -40 -60 -80 -100 -120 -140 -160 -180 -200 -220 -240 -260 -280 -300 -320
f1 (ppm)

2c

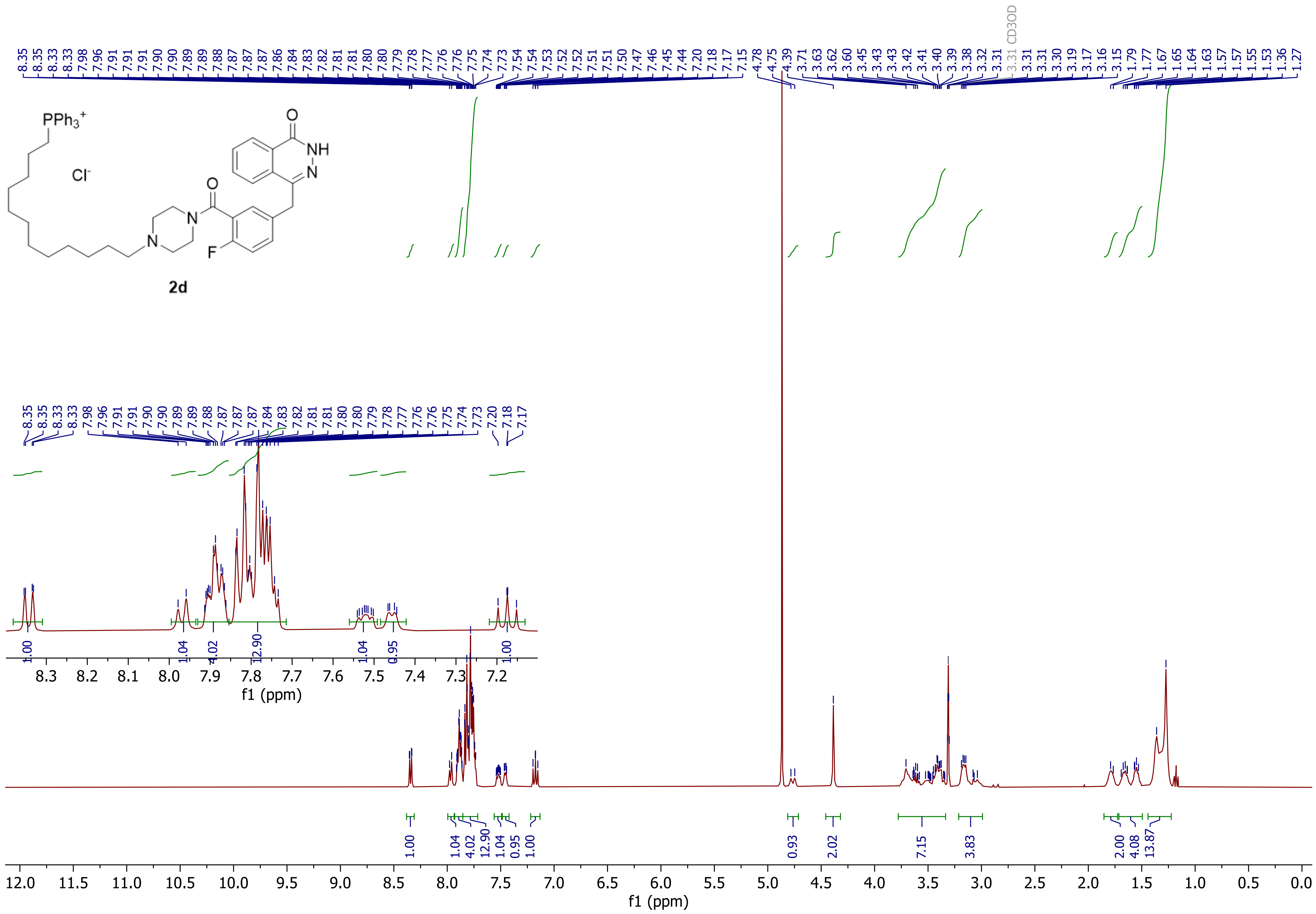

CD3OD
8.35 8.35 8.33 8.33 7.98 7.96 7.91 7.91 7.91 7.90 7.90 7.89 7.89 7.88 7.87 7.87 7.87 7.86 7.84 7.83 7.82 7.81 7.81 7.80 7.80 7.79 7.78 7.77 7.76 7.76 7.75 7.75 7.74 7.73 7.54 7.54 7.53 7.52 7.52 7.51 7.51 7.50 7.47 7.46 7.45 7.44 7.20 7.18 7.17 7.15 4.78 4.75 4.39 3.71 3.63 3.62 3.60 3.45 3.43 3.43 3.42 3.41 3.40 3.39 3.38 3.32 3.31 3.31 3.31 3.30 3.19 3.17 3.16 3.15 1.79 1.77 1.67 1.65 1.64 1.63 1.57 1.57 1.55 1.53 1.36 1.27
PPh3+
Cl-
O
NH
N
N
O
F
2d
1.00 1.04 4.02 12.90 1.04 0.95 1.00
f1 (ppm)
1.00 1.04 4.02 12.90 1.04 0.95 1.00 0.93 2.02 7.15 3.83 2.00 4.08 13.87
f1 (ppm)

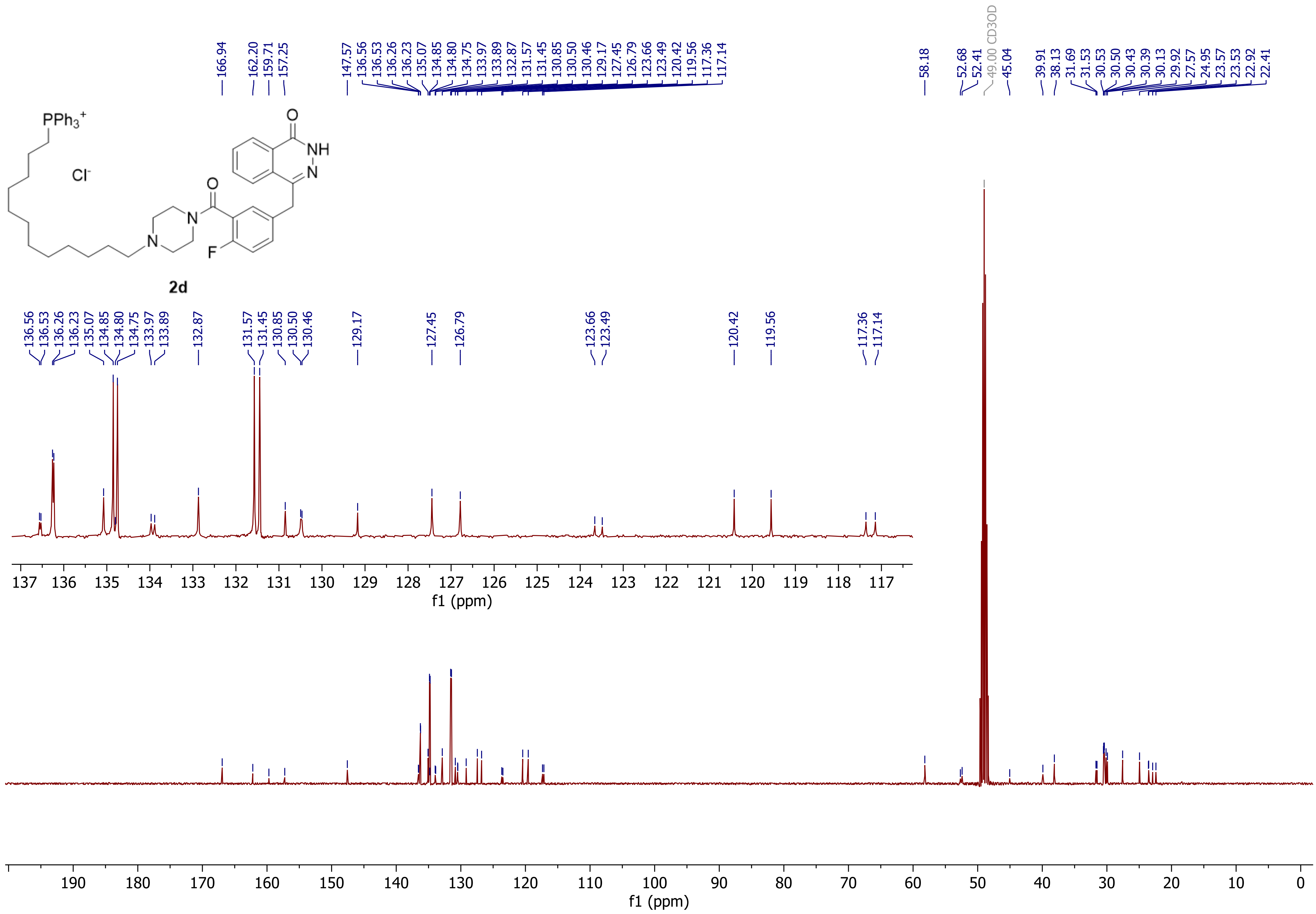
166.94
162.20
159.71
157.25
147.57
136.56
136.53
136.26
136.23
135.07
134.85
134.80
134.75
133.97
133.89
132.87
131.57
131.45
130.85
130.50
130.46
129.17
127.45
126.79
123.66
123.49
120.42
119.56
117.36
117.14
58.18
52.68
52.41
49.00 CD3OD
45.04
39.91
38.13
31.69
31.53
30.53
30.50
30.43
30.39
30.13
29.92
27.57
24.95
23.57
23.53
22.92
22.41
PPh3+
Cl-
O
NH
N
N
F
2d
f1 (ppm)
f1 (ppm)

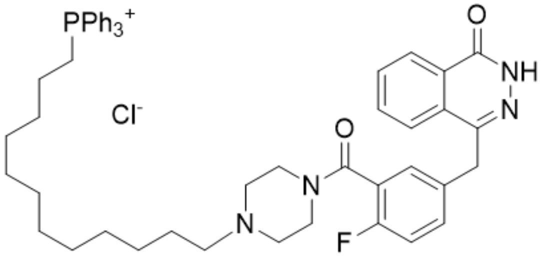
PPh3+
Cl-
O
NH
N
O
N
N
F

2d

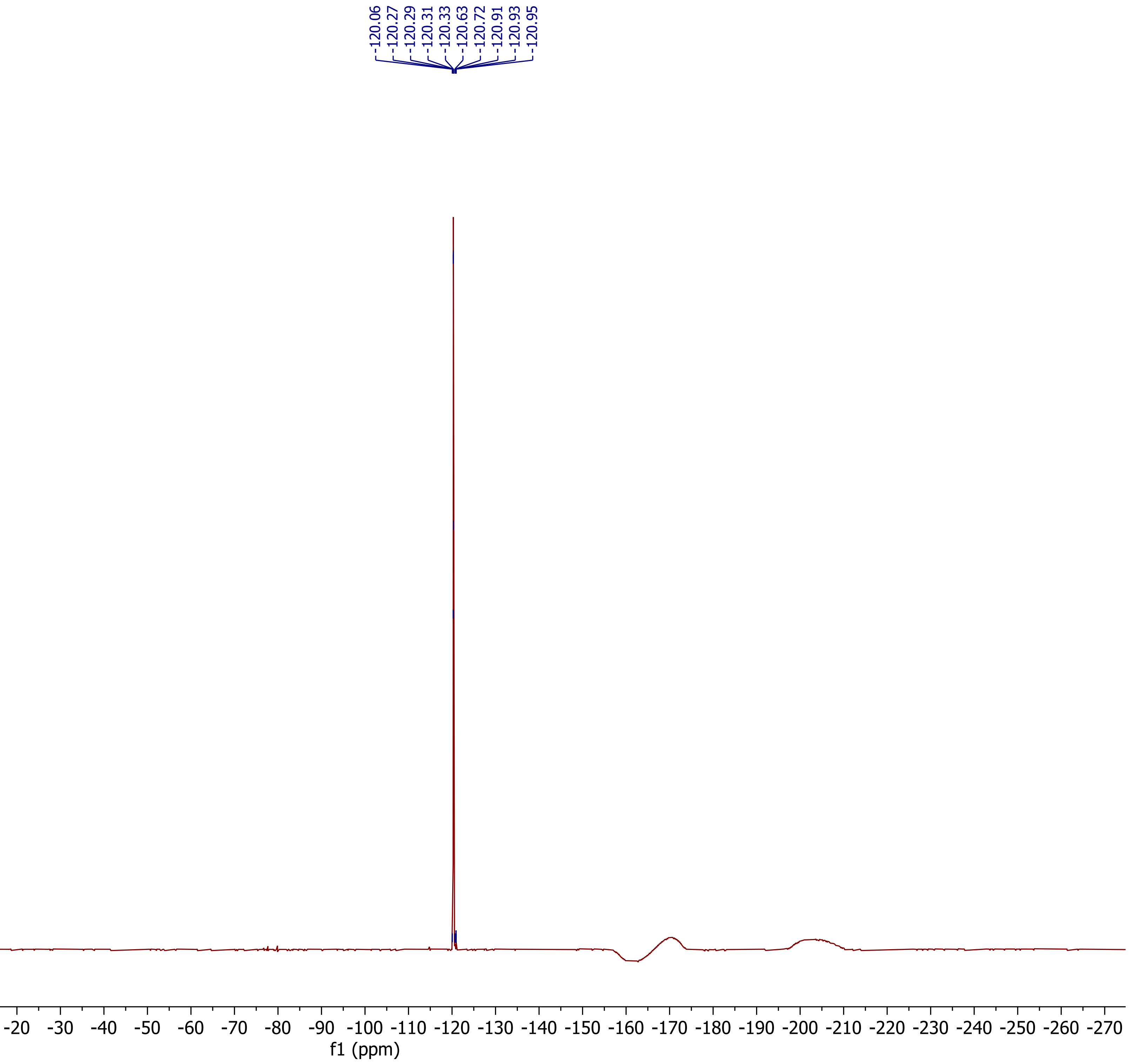
-120.06
-120.27
-120.29
-120.31
-120.33
-120.63
-120.72
-120.91
-120.93
-120.95
70 60 50 40 30 20 10 0 -10 -20 -30 -40 -50 -60 -70 -80 -90 -100 -110 -120 -130 -140 -150 -160 -170 -180 -190 -200 -210 -220 -230 -240 -250 -260 -270
f1 (ppm)

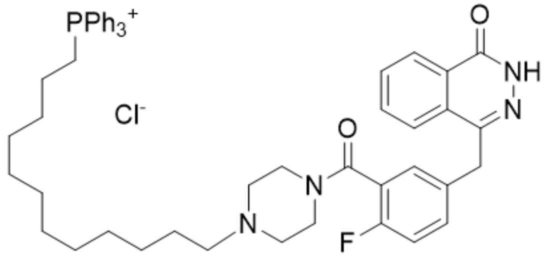
PPh3+
Cl-
O
NH
N
O
N
N
F

2d

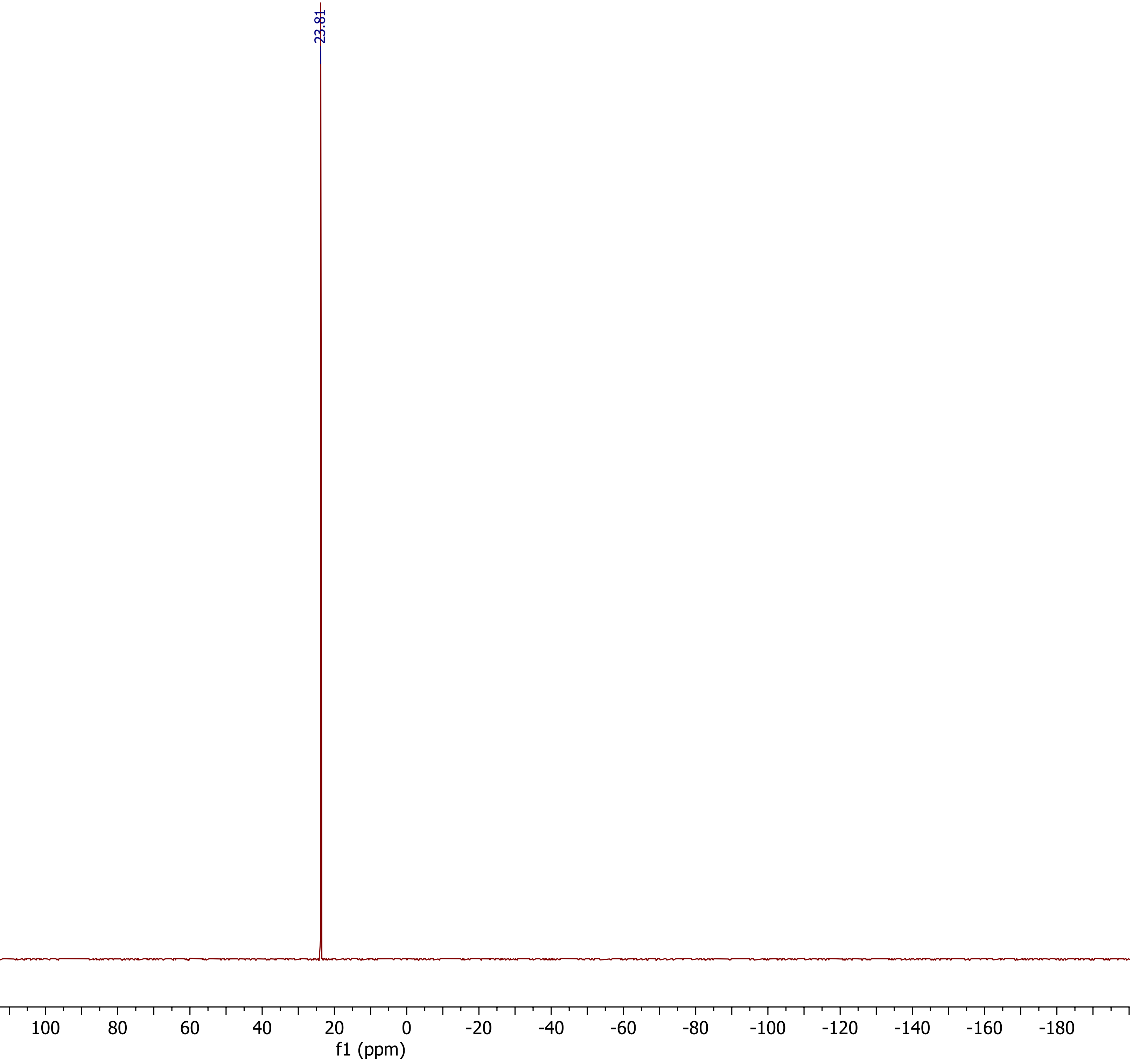
23.81
200
180
160
140
120
100
80
60
40
20
0
-20
-40
-60
-80
-100
-120
-140
-160
-180
f1 (ppm)

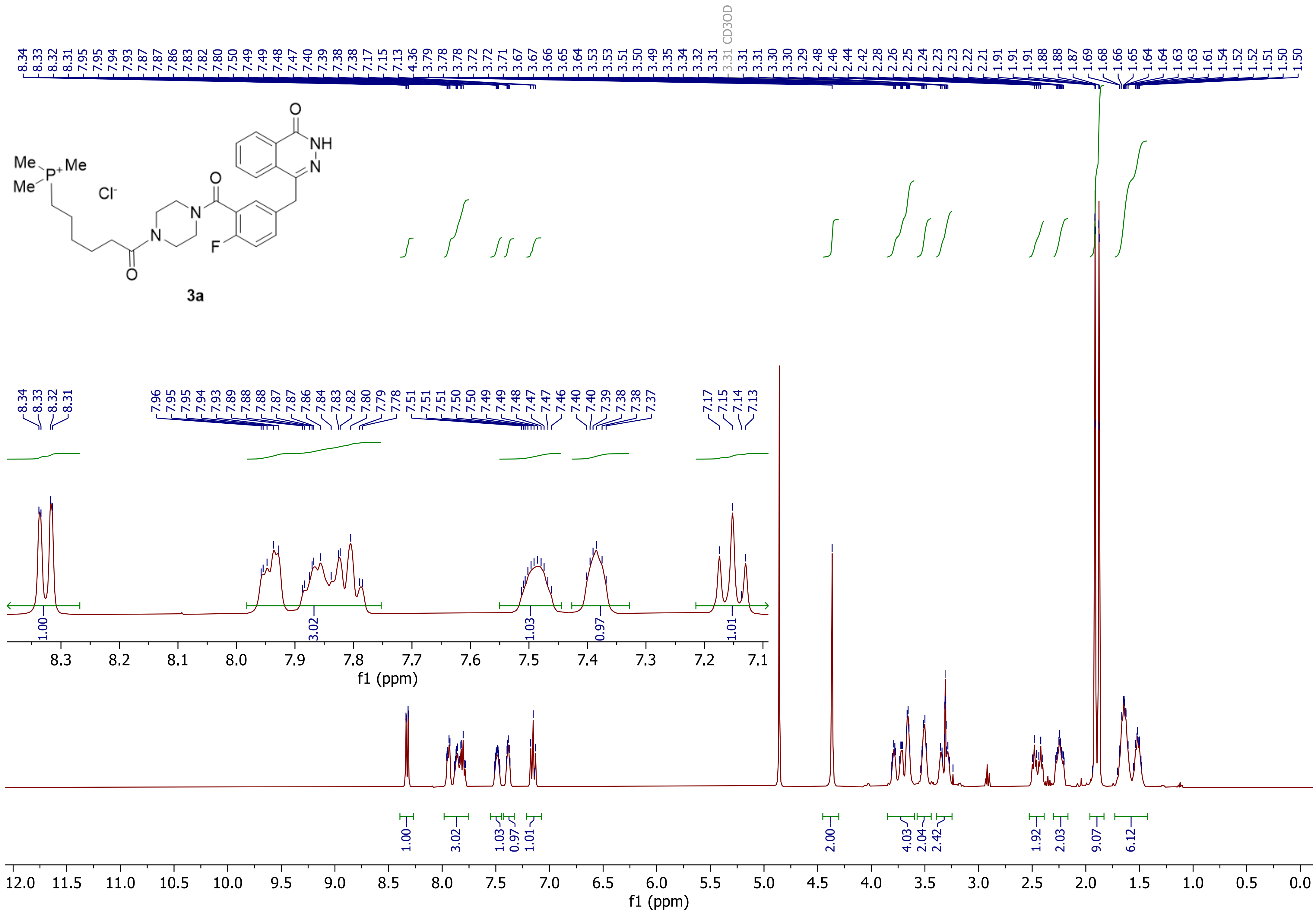

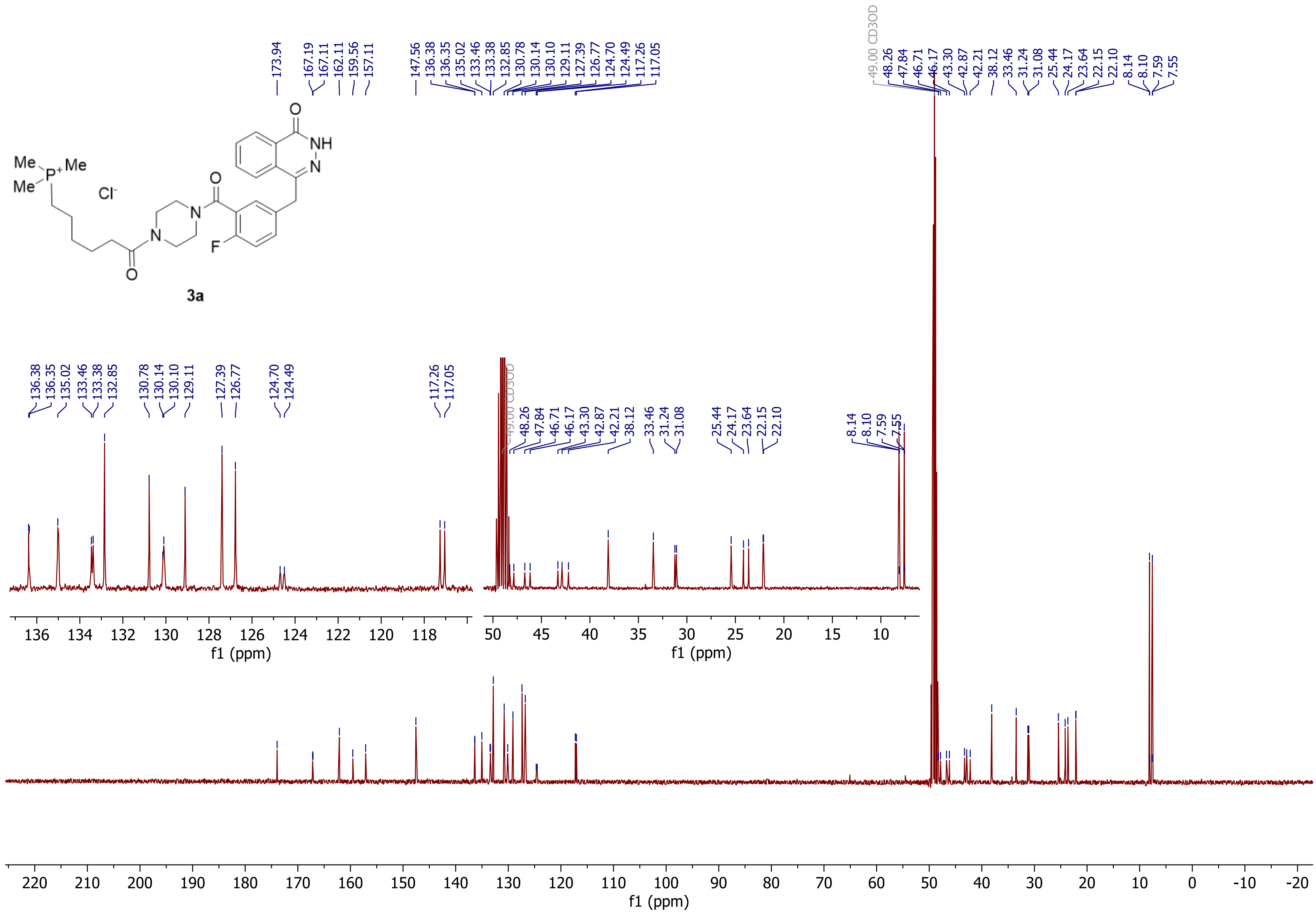

173.94
167.19
167.11
162.11
159.56
157.11
147.56
136.38
136.35
135.02
133.46
133.38
132.85
130.78
130.14
130.10
129.11
127.39
126.77
124.70
124.49
117.26
117.05
49.00 CD3OD
48.26
47.84
46.71
46.17
43.30
42.87
42.21
38.12
33.46
31.24
31.08
25.44
24.17
23.64
22.15
22.10
8.14
8.10
7.59
7.55
Me
Me
Me
P+
Cl-
O
NH
N
N
F
3a
f1 (ppm)

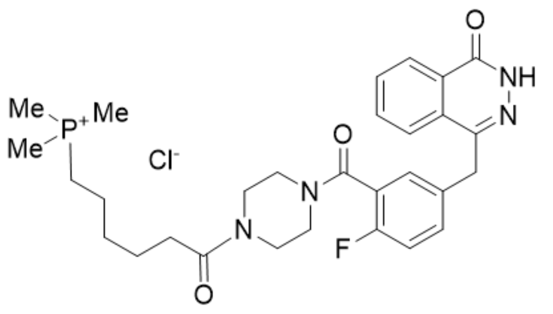

3a

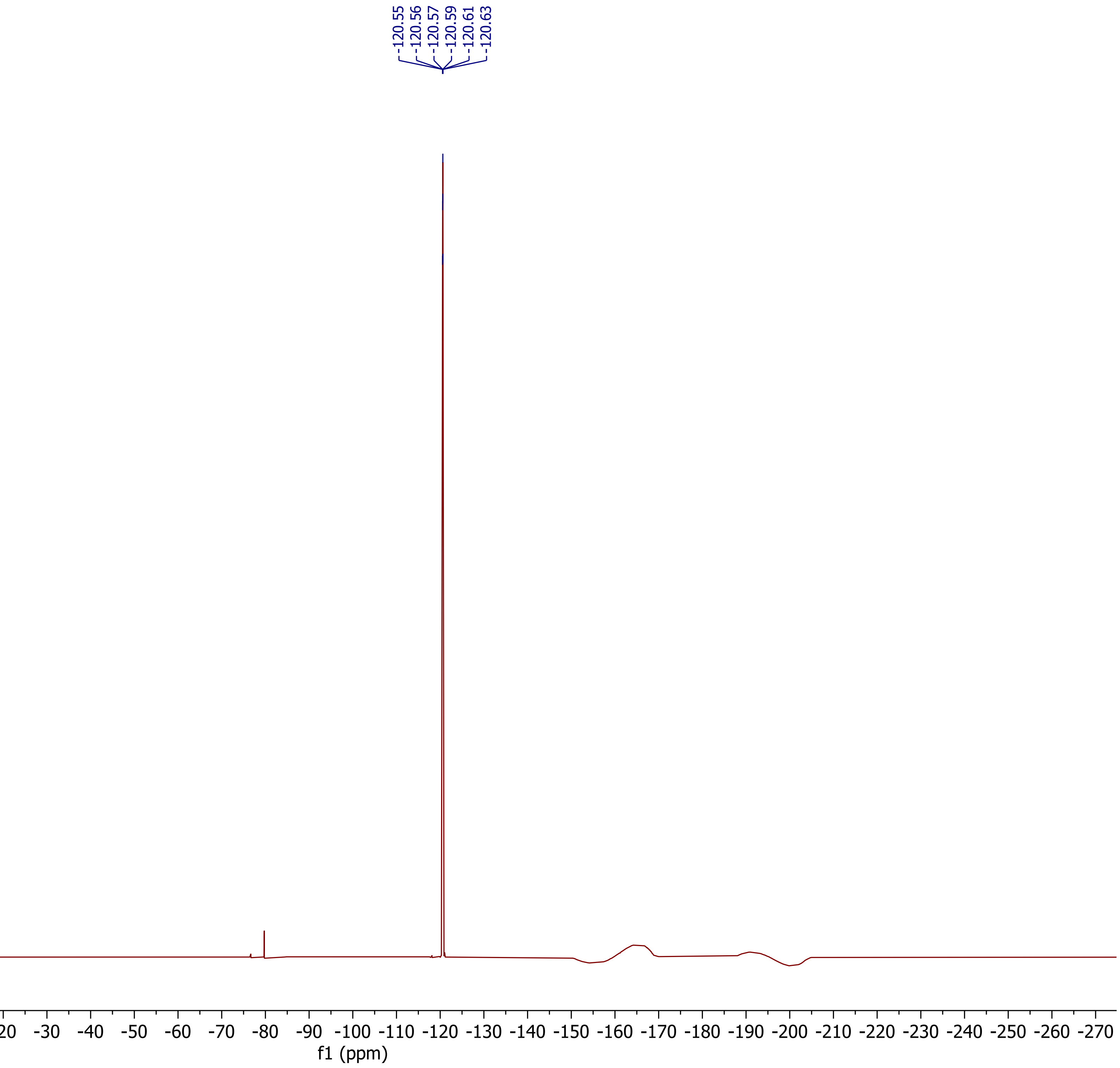

-120.55
-120.56
-120.57
-120.59
-120.61
-120.63
70 60 50 40 30 20 10 0 -10 -20 -30 -40 -50 -60 -70 -80 -90 -100 -110 -120 -130 -140 -150 -160 -170 -180 -190 -200 -210 -220 -230 -240 -250 -260 -270
f1 (ppm)

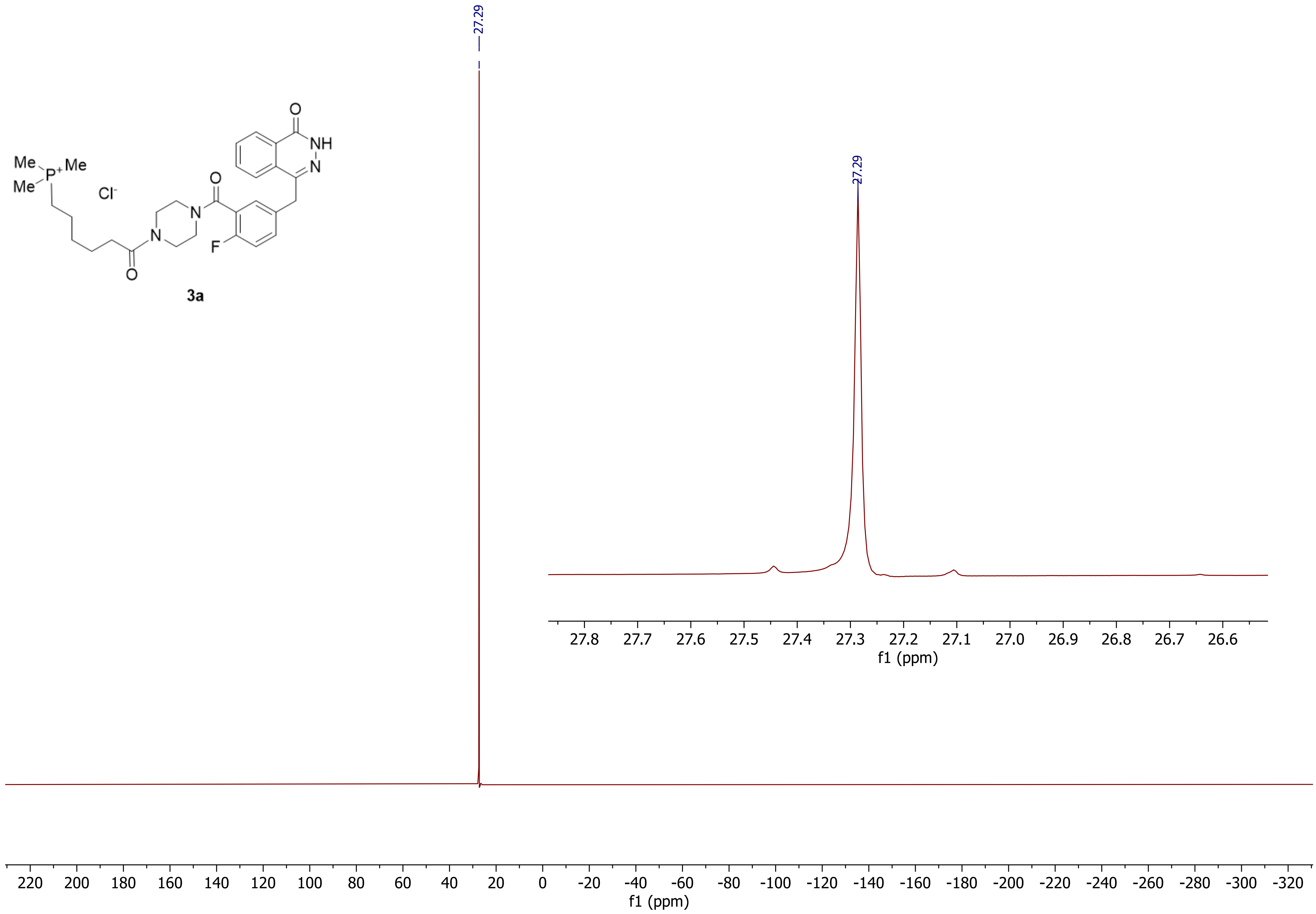
27.29
Me
Me
Me
P+
Cl-
O
NH
N
N
O
N
F
O
3a
27.29
27.8 27.7 27.6 27.5 27.4 27.3 27.2 27.1 27.0 26.9 26.8 26.7 26.6
f1 (ppm)
220 200 180 160 140 120 100 80 60 40 20 0 -20 -40 -60 -80 -100 -120 -140 -160 -180 -200 -220 -240 -260 -280 -300 -320
f1 (ppm)

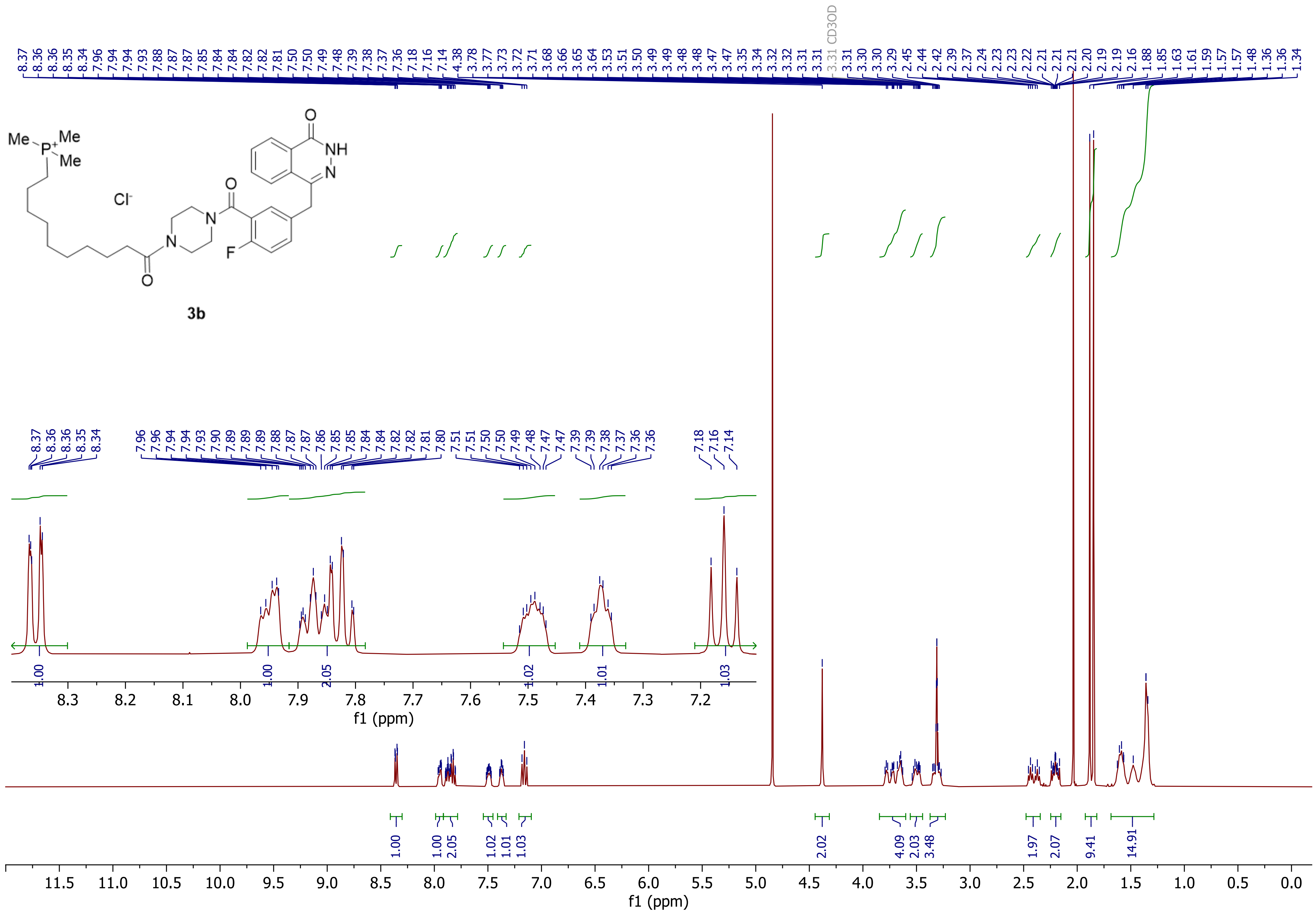
8.37 8.36 8.36 8.35 8.34 7.96 7.94 7.94 7.93 7.88 7.87 7.87 7.85 7.84 7.84 7.82 7.82 7.81 7.50 7.49 7.48 7.39 7.38 7.37 7.36 7.18 7.16 7.14 4.38 3.78 3.77 3.73 3.72 3.71 3.68 3.66 3.65 3.64 3.53 3.51 3.50 3.49 3.49 3.48 3.48 3.47 3.35 3.34 3.32 3.32 3.31 3.31 3.31 CD3OD 3.31 3.30 3.29 2.45 2.44 2.42 2.39 2.37 2.24 2.23 2.23 2.22 2.21 2.21 2.21 2.20 2.19 2.16 1.88 1.85 1.63 1.61 1.59 1.57 1.57 1.48 1.36 1.36 1.34
Me
Me
Me
P+
Cl-
O
NH
N
O
N
N
F
O
3b
8.37 8.36 8.36 8.35 8.34 7.96 7.96 7.94 7.94 7.93 7.90 7.89 7.89 7.88 7.87 7.87 7.86 7.85 7.85 7.84 7.84 7.82 7.82 7.81 7.80 7.51 7.51 7.50 7.50 7.49 7.48 7.47 7.47 7.39 7.39 7.38 7.37 7.36 7.36 7.18 7.16 7.14
1.00 1.00 2.05 1.02 1.01 1.03
f1 (ppm)
1.00 1.00 2.05 1.02 1.01 1.03 2.02 4.09 2.03 3.48 1.97 2.07 9.41 14.91
f1 (ppm)

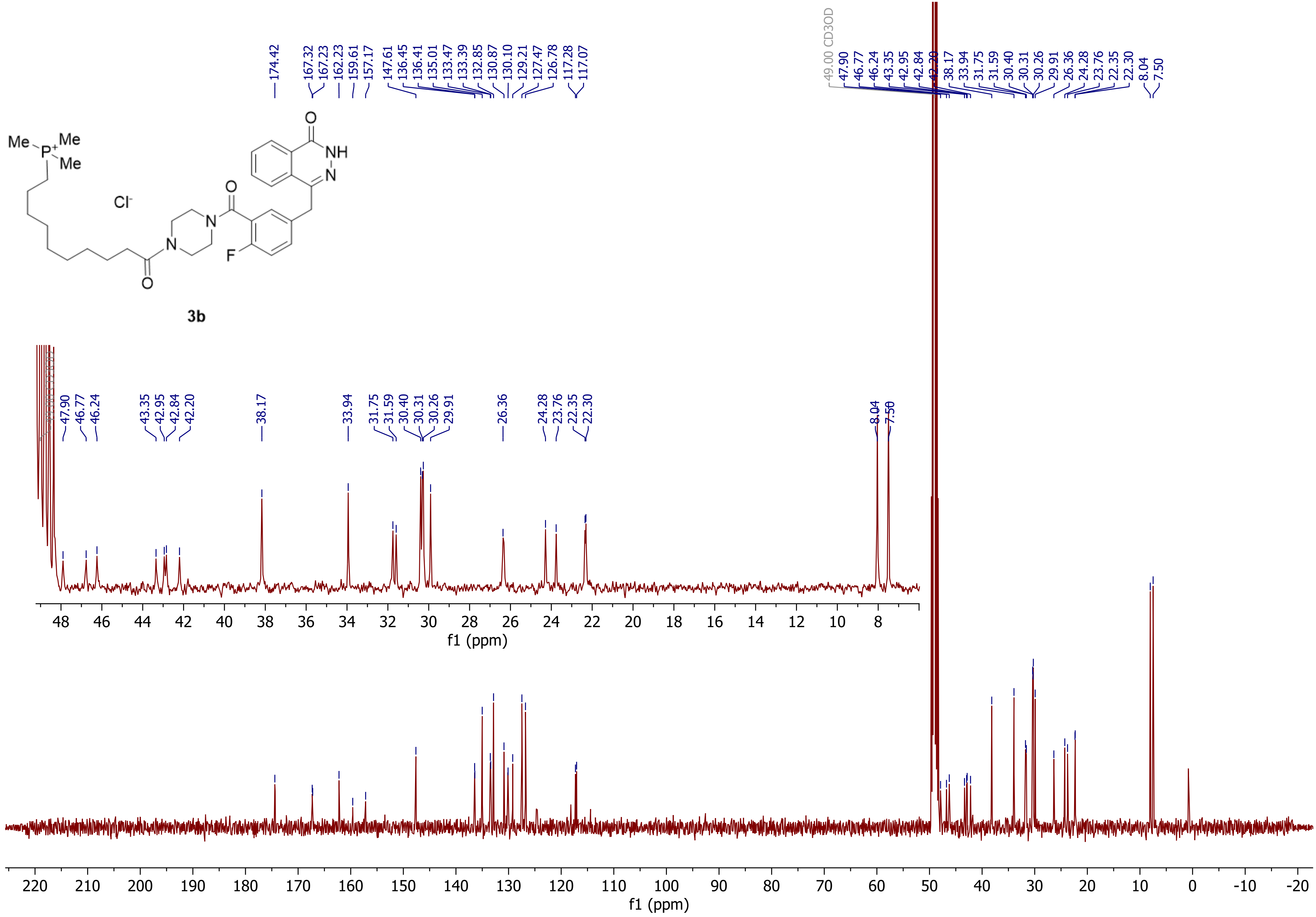
3b
174.42
167.32
167.23
162.23
159.61
157.17
147.61
136.45
136.41
135.01
133.47
133.39
132.85
130.87
130.10
129.21
127.47
126.78
117.28
117.07
49.00 CD3OD
47.90
46.77
46.24
43.35
42.95
42.84
38.17
33.94
31.75
31.59
30.40
30.31
30.26
29.91
26.36
24.28
23.76
22.35
22.30
8.04
7.50
f1 (ppm)

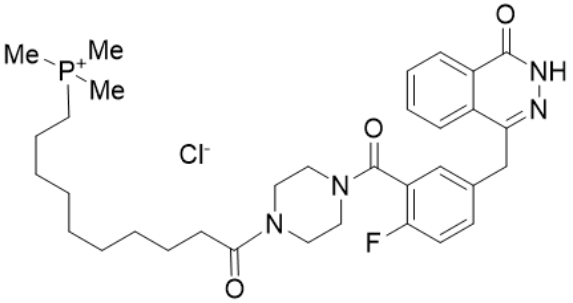
Me
P+
Me
Me
Cl-
O
NH
N
O
N
N
F
O

3b

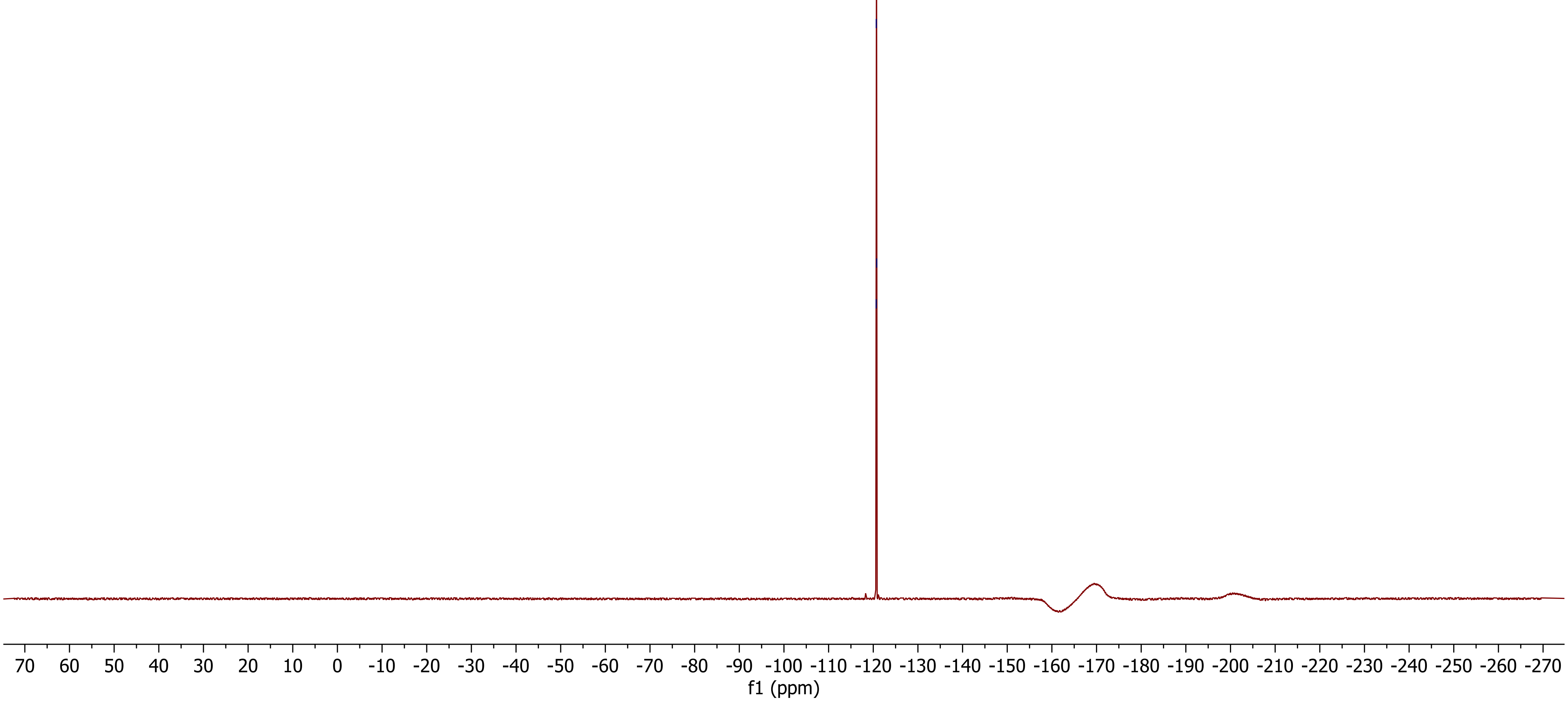
-120.66
-120.67
-120.68
-120.70
-120.71
-120.73
-120.74
-120.75
-120.77
70 60 50 40 30 20 10 0 -10 -20 -30 -40 -50 -60 -70 -80 -90 -100 -110 -120 -130 -140 -150 -160 -170 -180 -190 -200 -210 -220 -230 -240 -250 -260 -270
f1 (ppm)

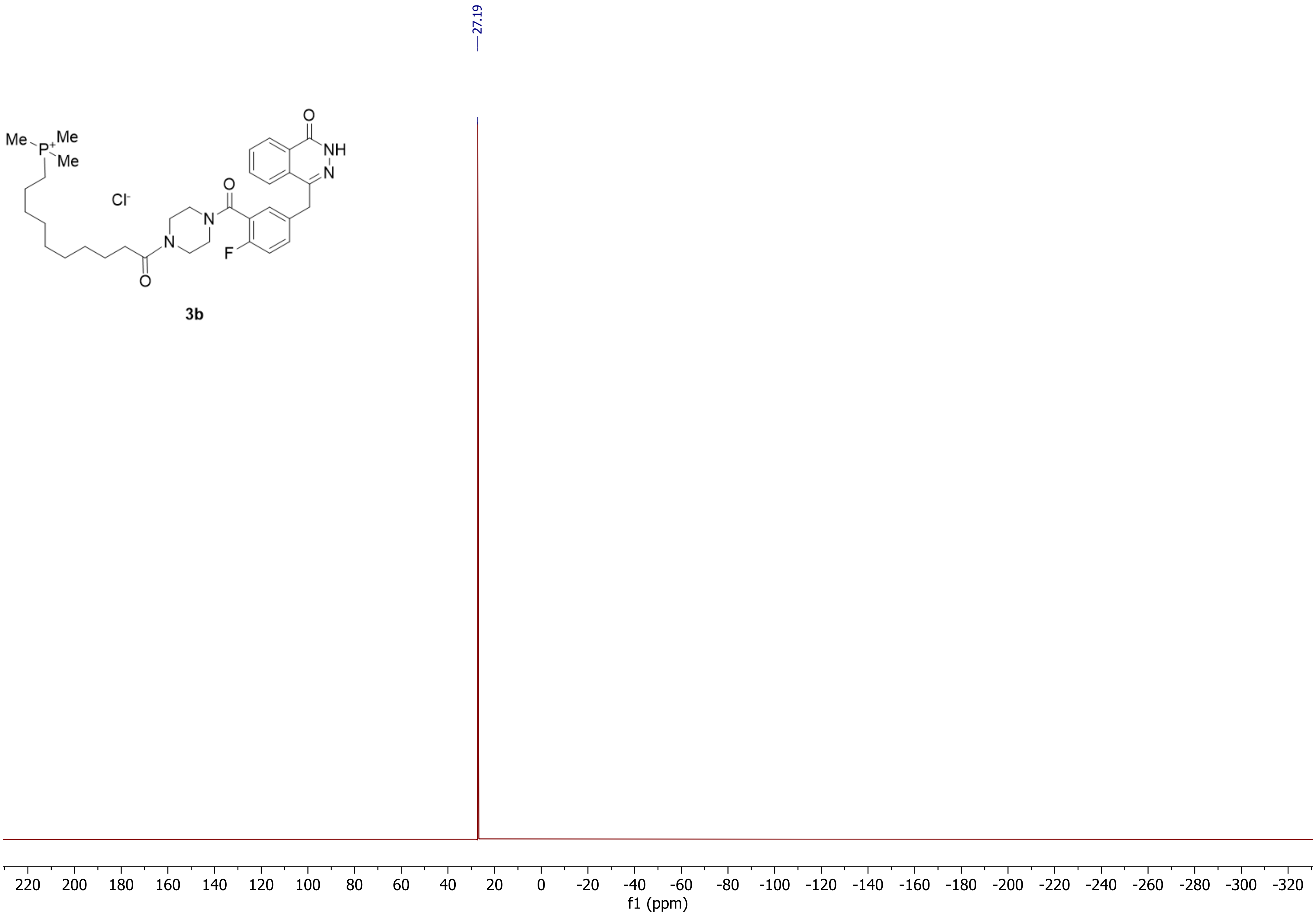
27.19
Me
Me
Me
P+
Cl-
O
NH
N
N
N
O
F
O
3b
220 200 180 160 140 120 100 80 60 40 20 0 -20 -40 -60 -80 -100 -120 -140 -160 -180 -200 -220 -240 -260 -280 -300 -320
f1 (ppm)

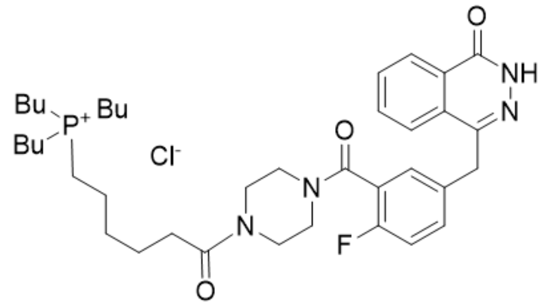
Bu
Bu
Bu
P+
Cl-
O
NH
N
N
F

3c

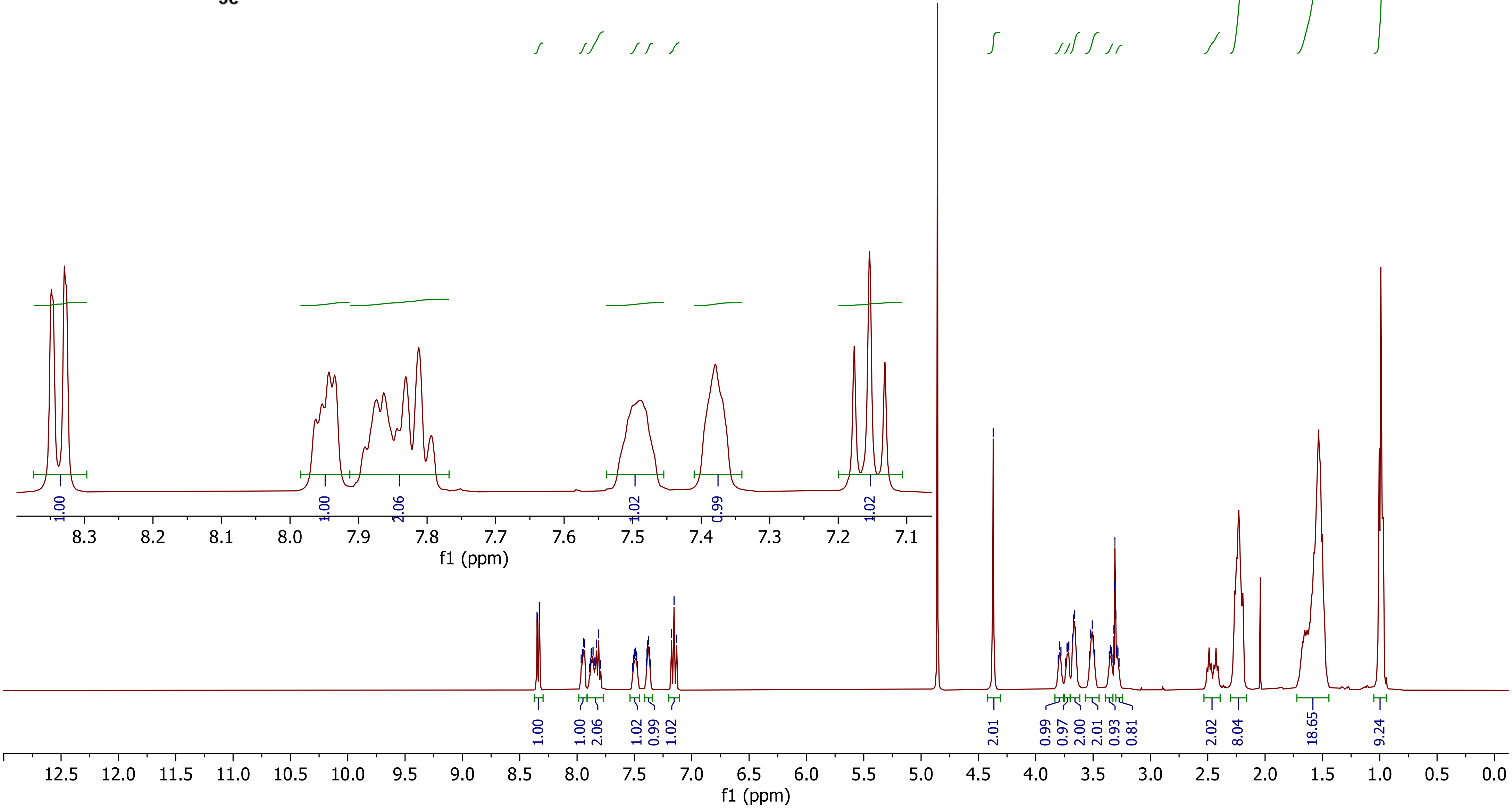
8.35 8.35 8.33 8.33 7.96 7.95 7.94 7.93 7.89 7.88 7.88 7.87 7.86 7.84 7.83 7.81 7.79 7.51 7.51 7.50 7.50 7.49 7.48 7.47 7.40 7.39 7.38 7.38 7.37 7.36 7.18 7.15 7.13 4.37
3.79 3.78 3.73 3.72 3.71 3.68 3.67 3.66 3.65 3.64 3.53 3.52 3.51 3.50 3.49 3.36 3.35 3.34 3.33 3.32 3.31 3.31 CD3OD 3.31 3.31 3.30 3.30 3.29 3.28
1.00 1.00 2.06 1.02 0.99 1.02
2.01 0.99 0.97 2.00 2.01 0.93 0.81 2.02 8.04 18.65 9.24
f1 (ppm)
f1 (ppm)

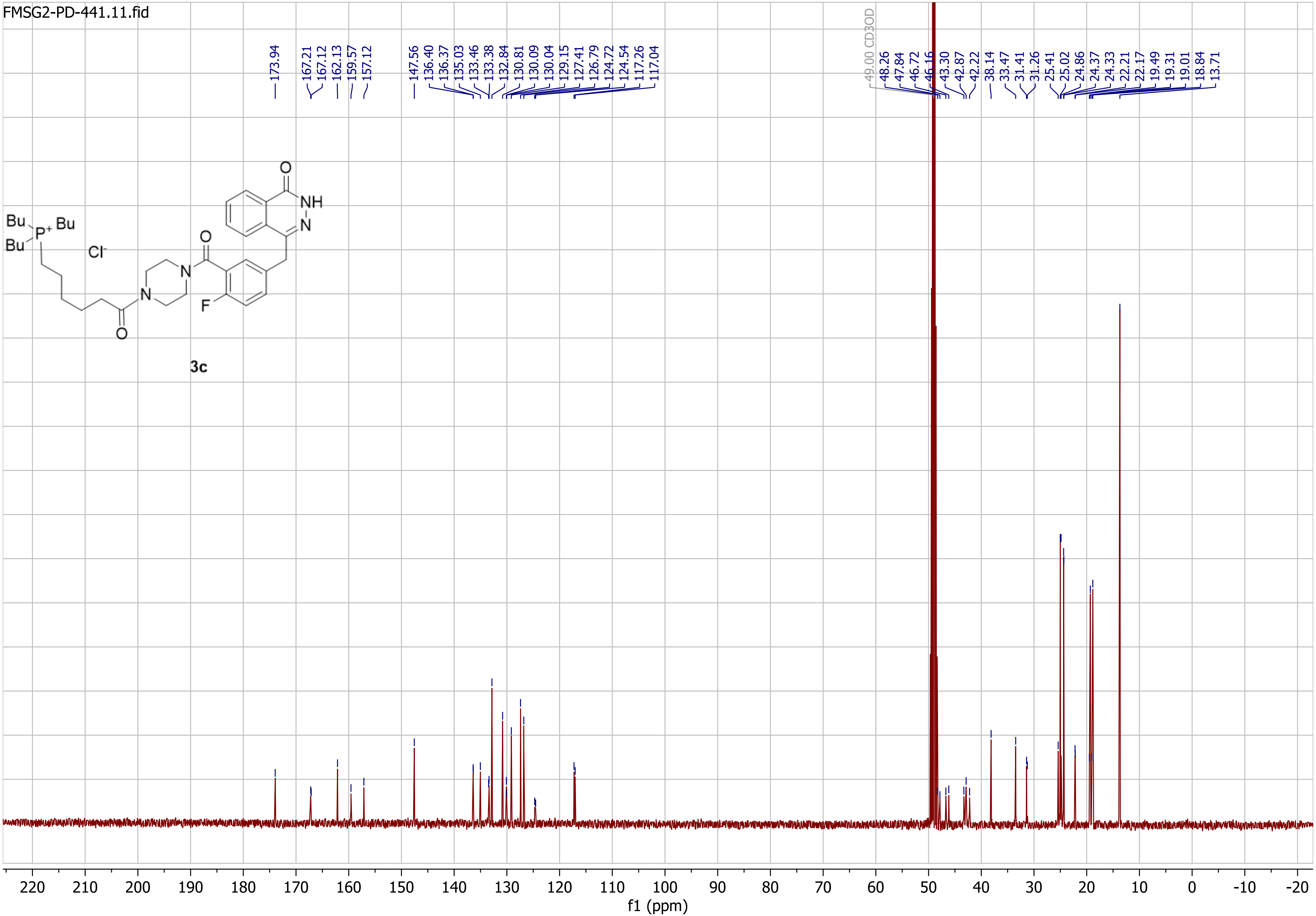

FMSG2-PD-441.11.fid
173.94
167.21
167.12
162.13
159.57
157.12
147.56
136.40
136.37
135.03
133.46
133.38
132.84
130.81
130.09
130.04
129.15
127.41
126.79
124.72
124.54
117.26
117.04
49.00 CD3OD
48.26
47.84
46.72
46.16
43.30
42.87
42.22
38.14
33.47
31.41
31.26
25.41
25.02
24.86
24.37
24.33
22.21
22.17
19.49
19.31
19.01
18.84
13.71
Bu
Bu
Bu
P+
Cl-
O
NH
N
N
O
F
3c
220 210 200 190 180 170 160 150 140 130 120 110 100 90 80 70 60 50 40 30 20 10 0 -10 -20
f1 (ppm)

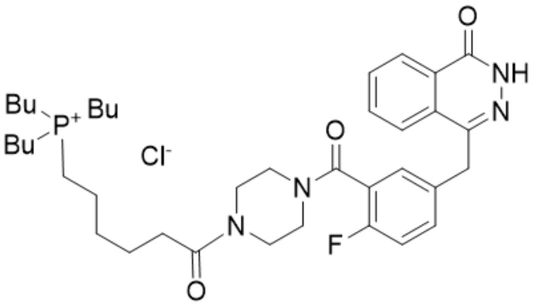
Bu
Bu
Bu
P+
Cl-
O
NH
N
O
N
N
F
O

3c

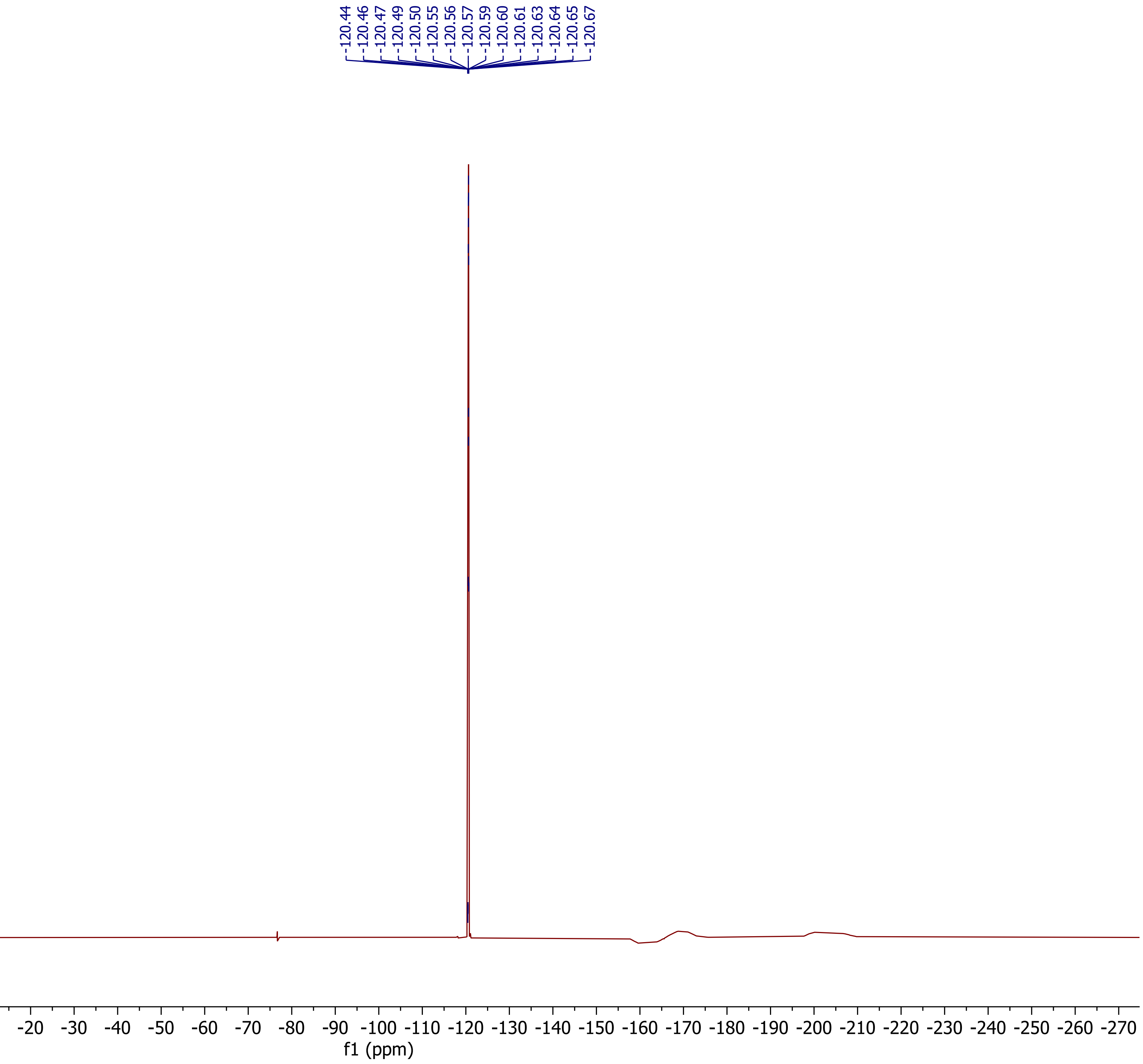
-120.44
-120.46
-120.47
-120.49
-120.50
-120.55
-120.56
-120.57
-120.59
-120.60
-120.61
-120.63
-120.64
-120.65
-120.67
70 60 50 40 30 20 10 0 -10 -20 -30 -40 -50 -60 -70 -80 -90 -100 -110 -120 -130 -140 -150 -160 -170 -180 -190 -200 -210 -220 -230 -240 -250 -260 -270
f1 (ppm)

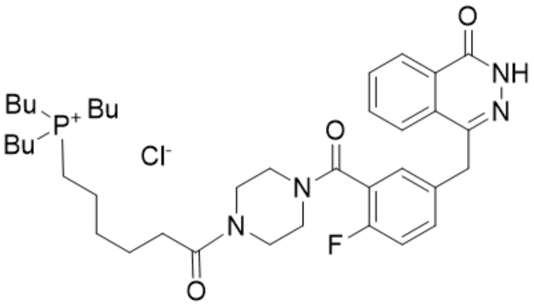
Bu
Bu
Bu
P+
Cl-
O
NH
N
N
N
O
O
F

3c

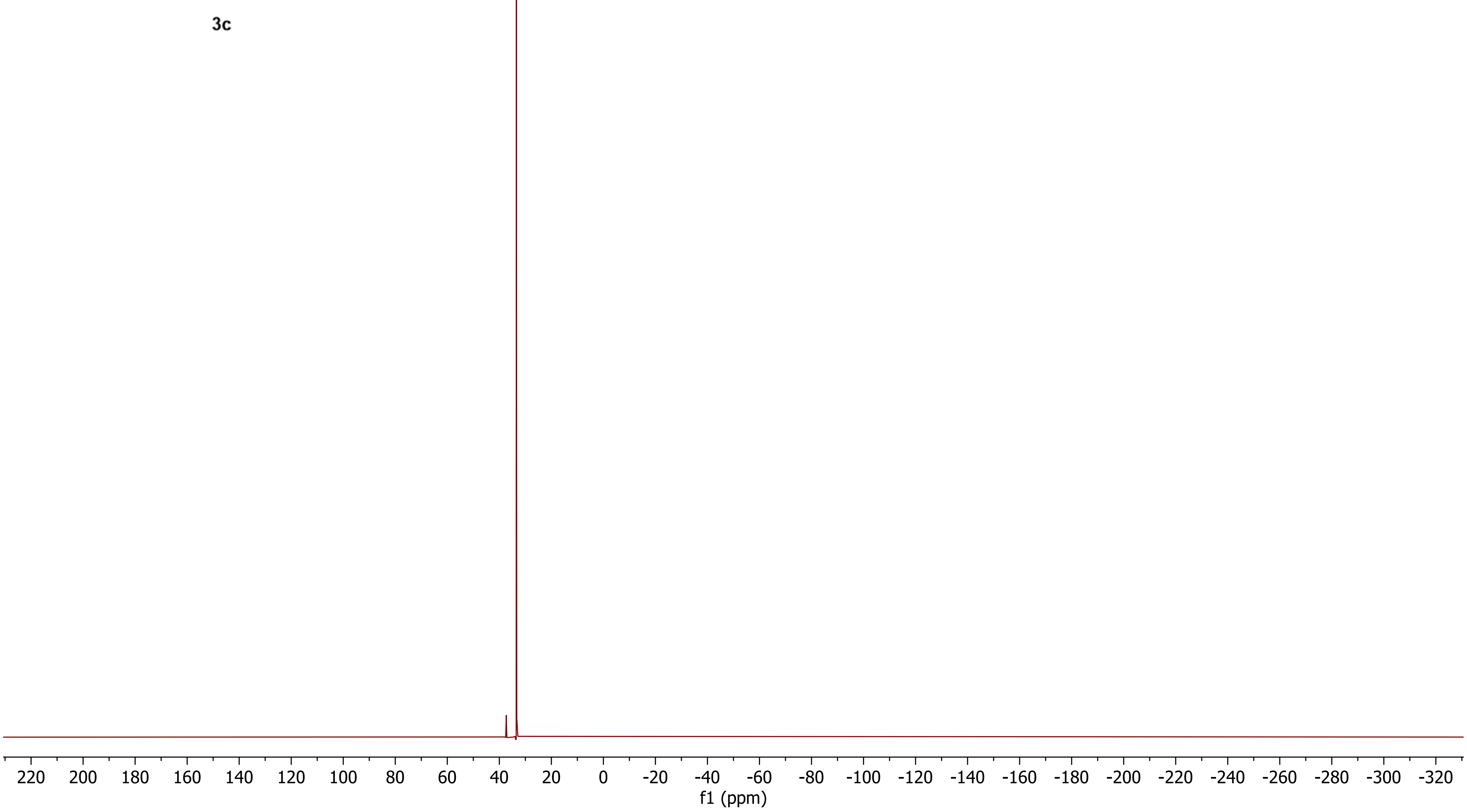
33.45
220 200 180 160 140 120 100 80 60 40 20 0 -20 -40 -60 -80 -100 -120 -140 -160 -180 -200 -220 -240 -260 -280 -300 -320
f1 (ppm)

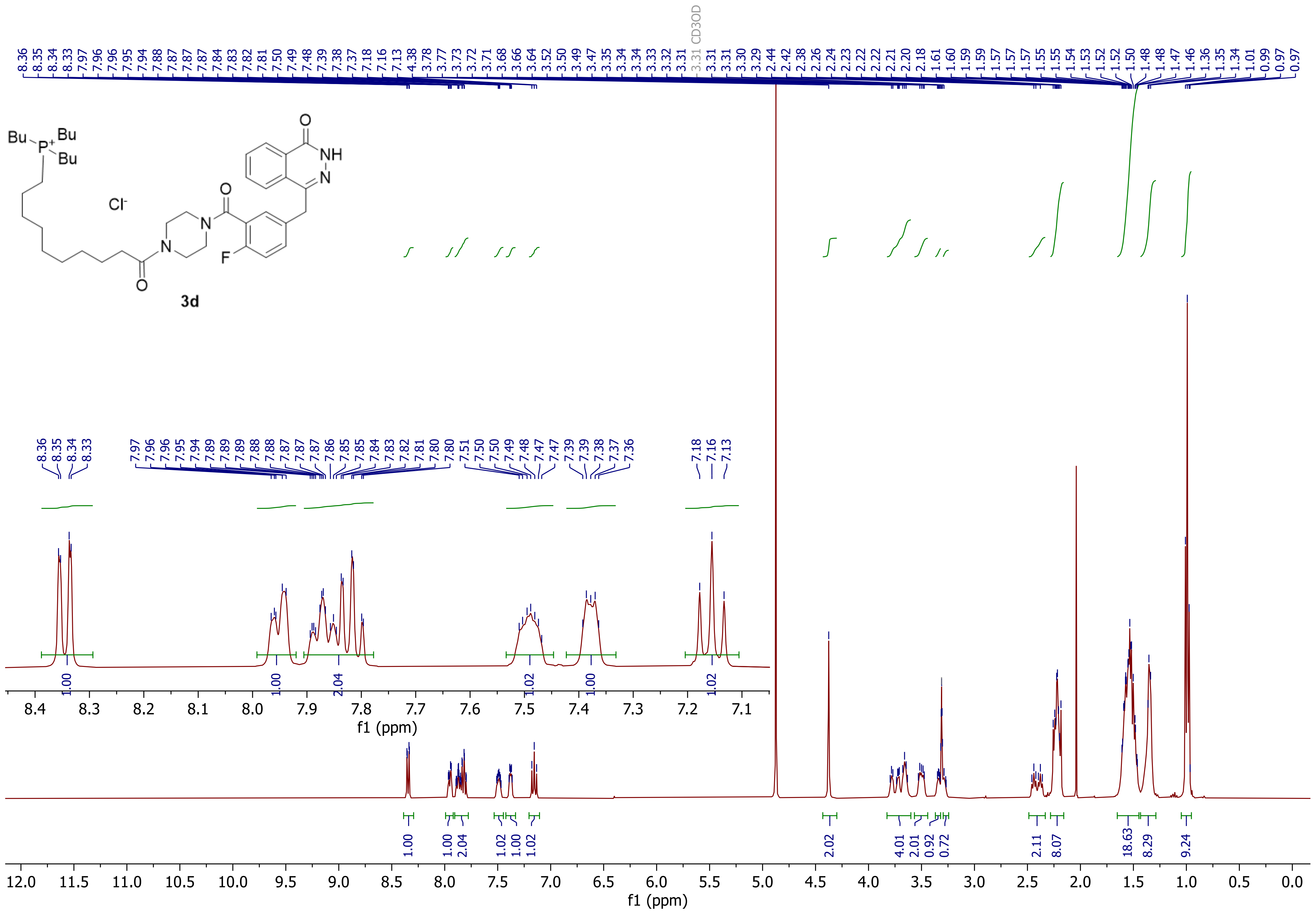

8.36 8.35 8.34 8.33 7.97 7.96 7.96 7.95 7.94 7.88 7.87 7.87 7.87 7.84 7.83 7.82 7.81 7.50 7.49 7.48 7.39 7.38 7.37 7.18 7.16 7.13 4.38 3.78 3.77 3.73 3.72 3.71 3.68 3.66 3.64 3.52 3.50 3.49 3.47 3.35 3.34 3.33 3.32 3.31 3.31 CD3OD 3.31 3.31 3.30 3.29 2.44 2.42 2.38 2.26 2.24 2.23 2.22 2.22 2.21 2.20 2.18 1.61 1.60 1.59 1.59 1.57 1.57 1.57 1.55 1.55 1.54 1.53 1.52 1.52 1.50 1.48 1.48 1.47 1.46 1.36 1.35 1.34 1.01 0.99 0.97 0.97
Bu
P+
Bu
Bu
Cl-
O
NH
N
O
N
N
F
O
3d
1.00 1.00 2.04 1.02 1.00 1.02 2.02 4.01 2.01 0.92 0.72 2.11 8.07 18.63 8.29 9.24
f1 (ppm)

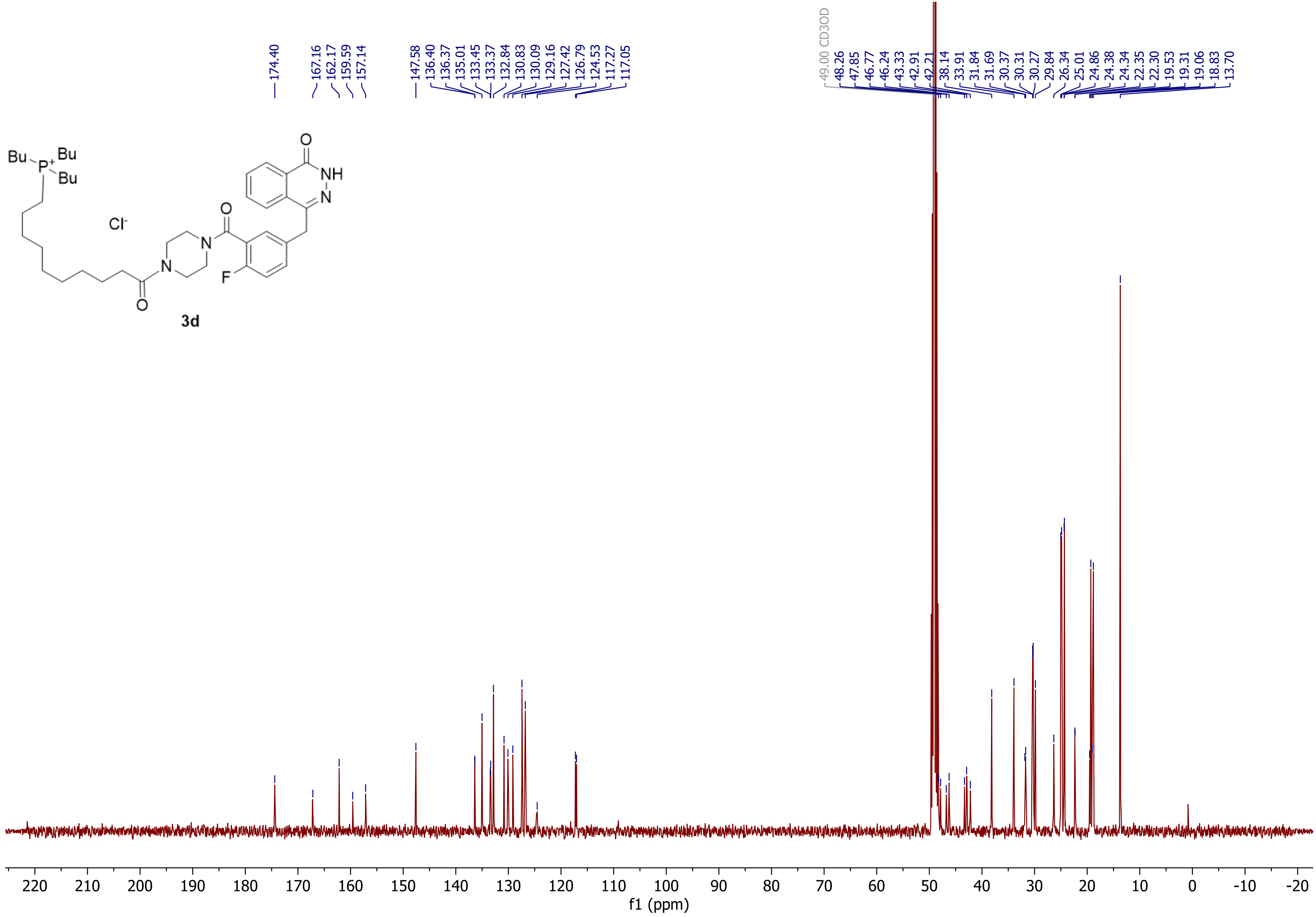

174.40
167.16
162.17
159.59
157.14
147.58
136.40
136.37
135.01
133.45
133.37
132.84
130.83
130.09
129.16
127.42
126.79
124.53
117.27
117.05
49.00 CD3OD
48.26
47.85
46.77
46.24
43.33
42.91
42.21
38.14
33.91
31.84
31.69
30.37
30.31
30.27
29.84
26.34
25.01
24.86
24.38
24.34
22.35
22.30
19.53
19.31
19.06
18.83
13.70
Bu
Bu
Bu
Cl-
O
NH
N
O
N
N
F
O
3d
f1 (ppm)

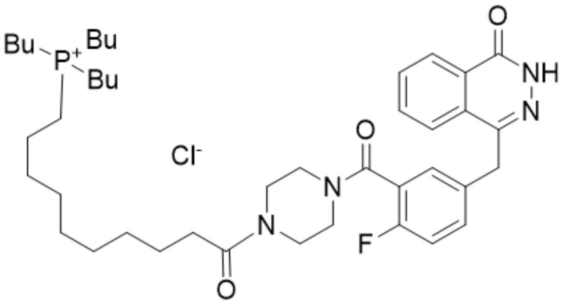
Bu
P+
Bu
Bu
Cl-
O
NH
N
O
N
N
F
O

3d

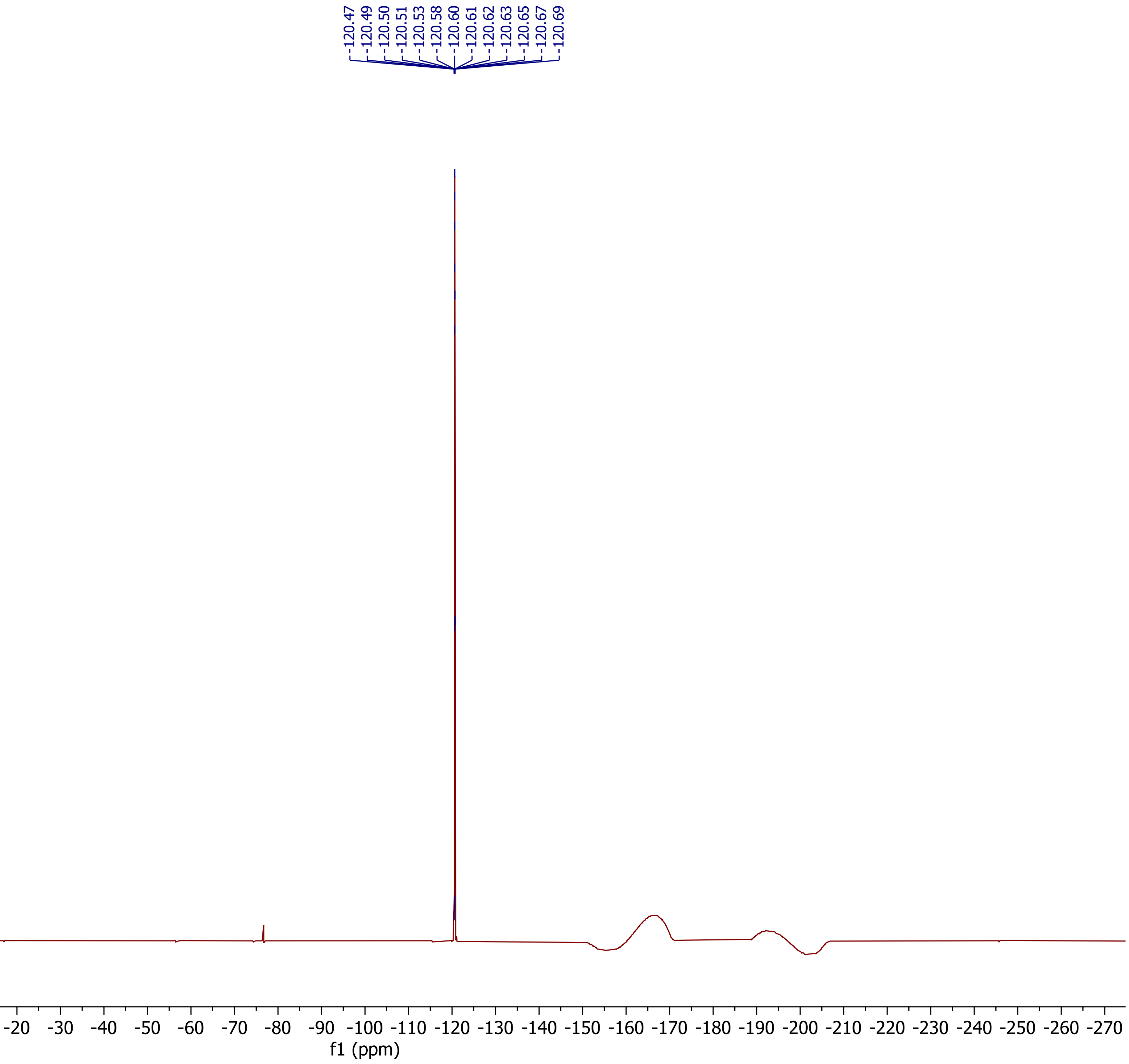
-120.47
-120.49
-120.50
-120.51
-120.53
-120.58
-120.60
-120.61
-120.62
-120.63
-120.65
-120.67
-120.69
70 60 50 40 30 20 10 0 -10 -20 -30 -40 -50 -60 -70 -80 -90 -100 -110 -120 -130 -140 -150 -160 -170 -180 -190 -200 -210 -220 -230 -240 -250 -260 -270
f1 (ppm)

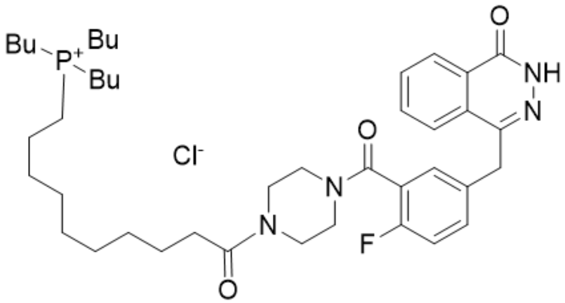
Bu
Bu
Bu
Cl-
O
NH
N
N
N
F
O

3d

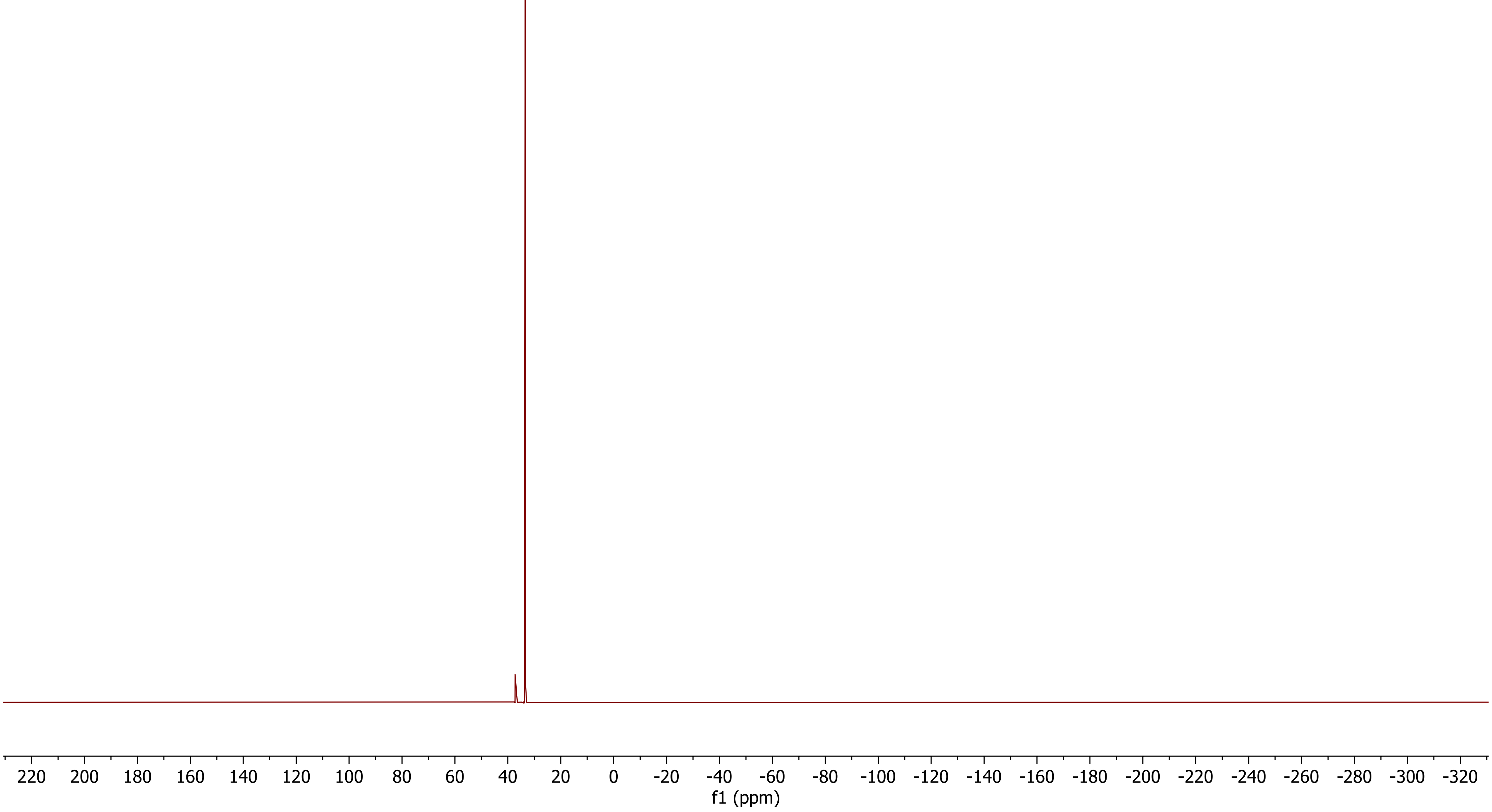
33.41
220
200
180
160
140
120
100
80
60
40
20
0
-20
-40
-60
-80
-100
-120
-140
-160
-180
-200
-220
-240
-260
-280
-300
-320
f1 (ppm)

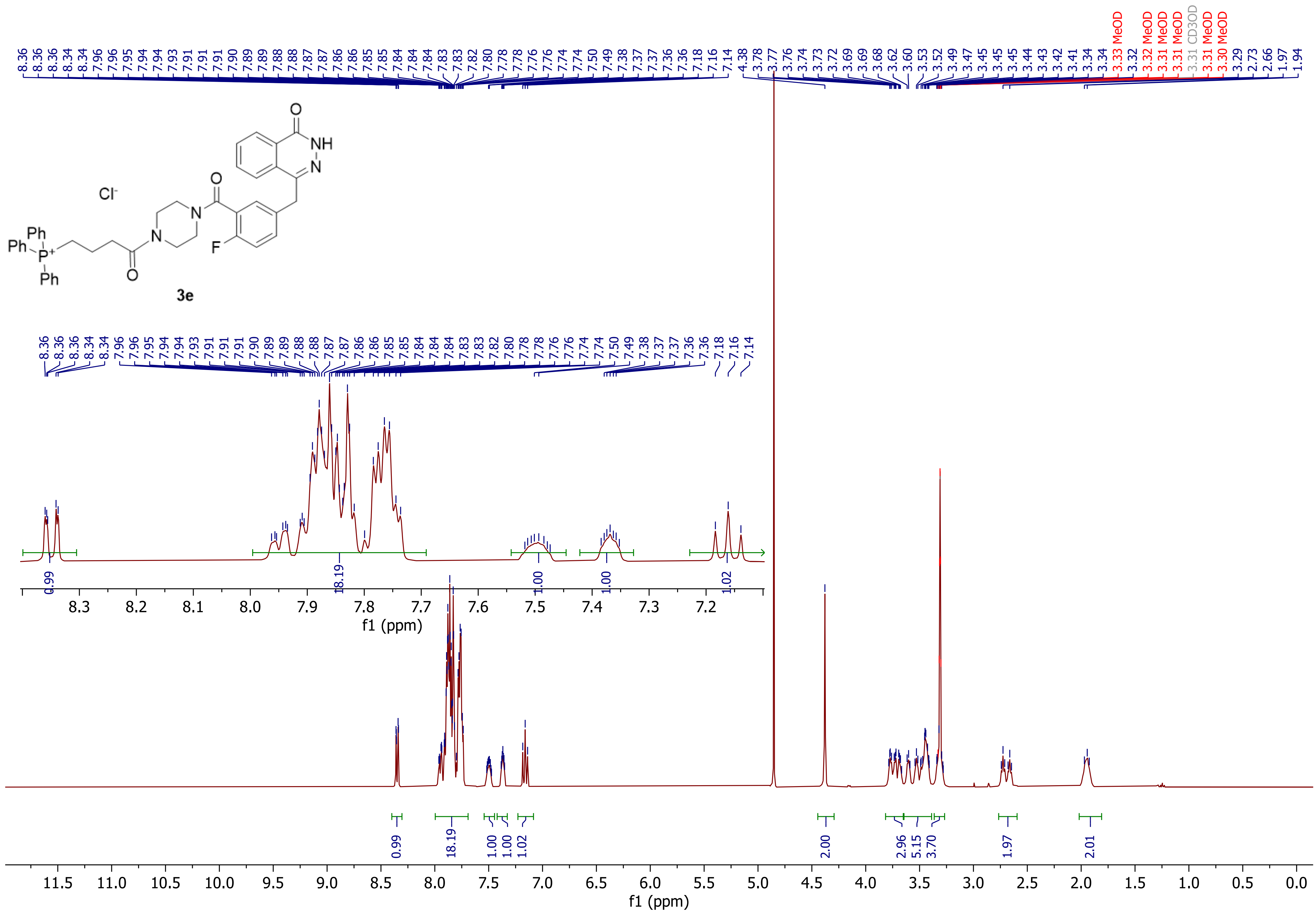

Cl⁻
Ph
Ph
P⁺
Ph
O
N
N
O
F
O
NH
N
3e
f1 (ppm)
f1 (ppm)
MeOD
CD3OD

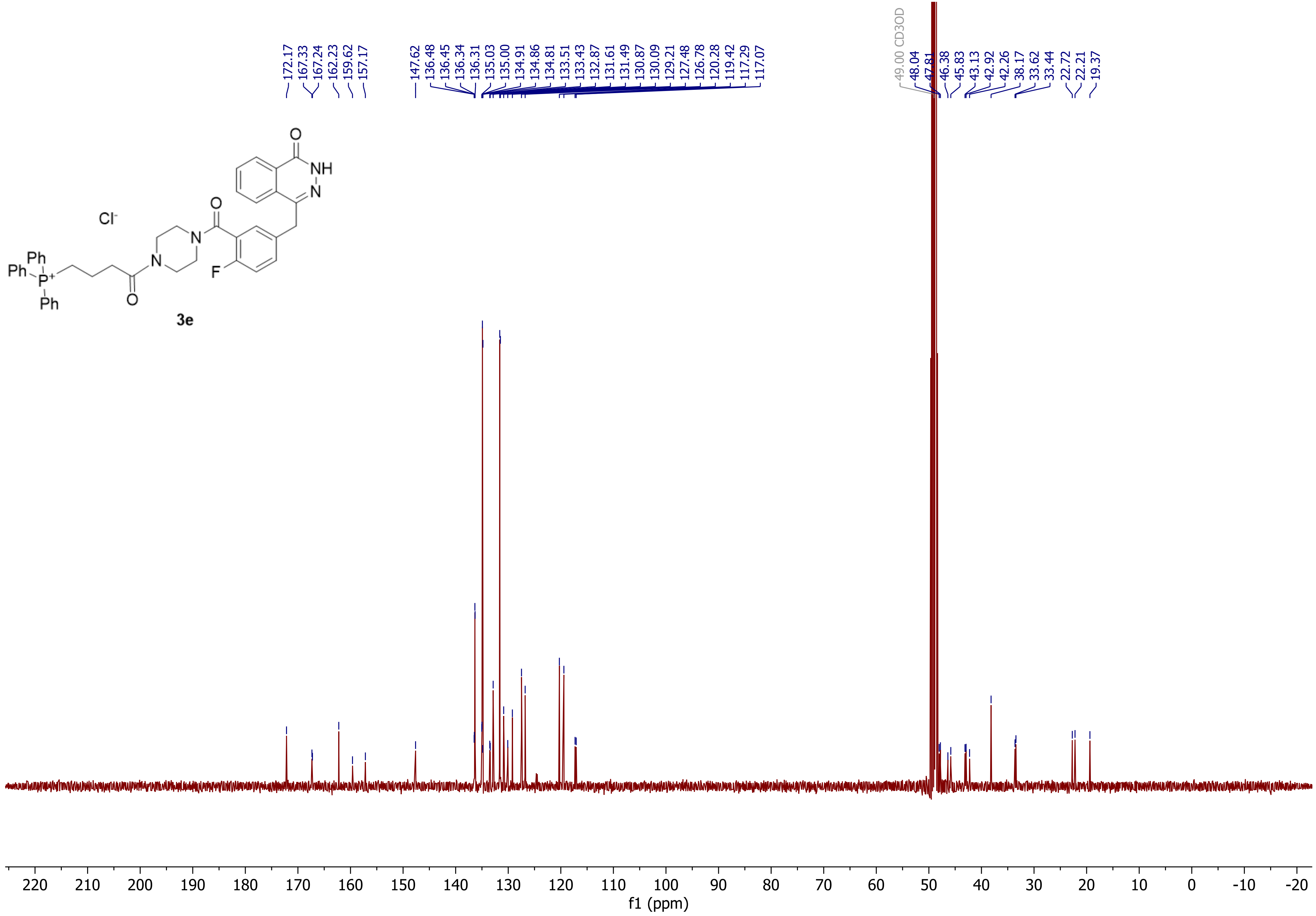

172.17
167.33
167.24
162.23
159.62
157.17
147.62
136.48
136.45
136.34
136.31
135.03
135.00
134.91
134.86
134.81
133.51
133.43
132.87
131.61
131.49
130.87
130.09
129.21
127.48
126.78
120.28
119.42
117.29
117.07
49.00 CD3OD
48.04
47.81
46.38
45.83
43.13
42.92
42.26
38.17
33.62
33.44
22.72
22.21
19.37
Cl⁻
Ph
Ph
P⁺
Ph
O
N
N
O
F
O
NH
N
O
3e
220 210 200 190 180 170 160 150 140 130 120 110 100 90 80 70 60 50 40 30 20 10 0 -10 -20
f1 (ppm)

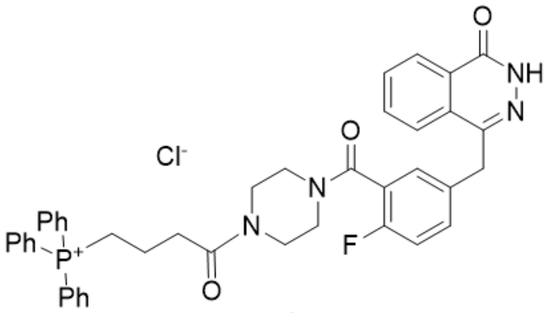

Cl⁻
Ph
Ph
P⁺
Ph
O
N
N
O
F
O
NH
N

3e

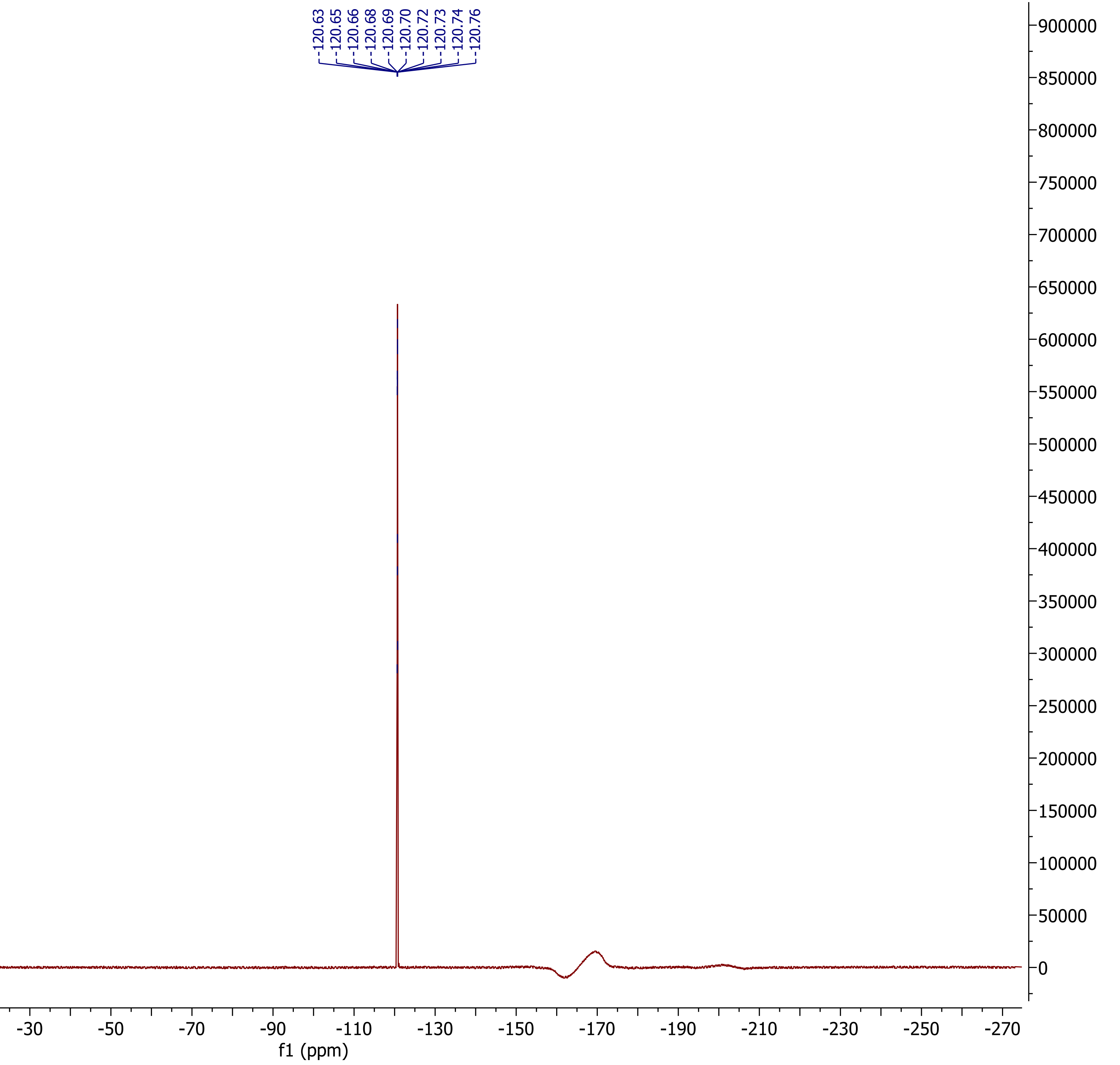

-120.63
-120.65
-120.66
-120.68
-120.69
-120.70
-120.72
-120.73
-120.74
-120.76
900000
850000
800000
750000
700000
650000
600000
550000
500000
450000
400000
350000
300000
250000
200000
150000
100000
50000
0
70 50 30 10 -10 -30 -50 -70 -90 -110 -130 -150 -170 -190 -210 -230 -250 -270
f1 (ppm)

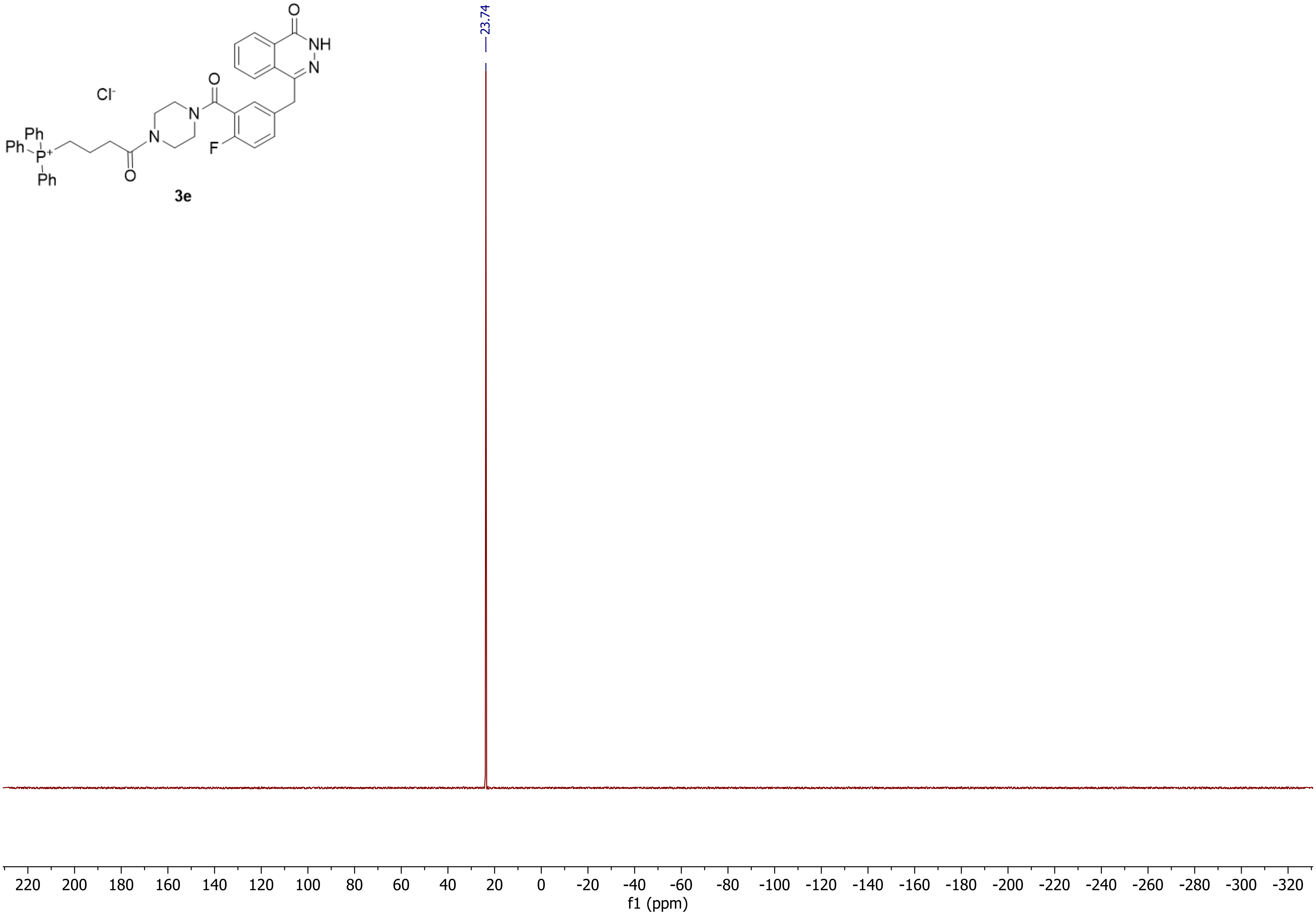

3e

23.74
220 200 180 160 140 120 100 80 60 40 20 0 -20 -40 -60 -80 -100 -120 -140 -160 -180 -200 -220 -240 -260 -280 -300 -320
f1 (ppm)

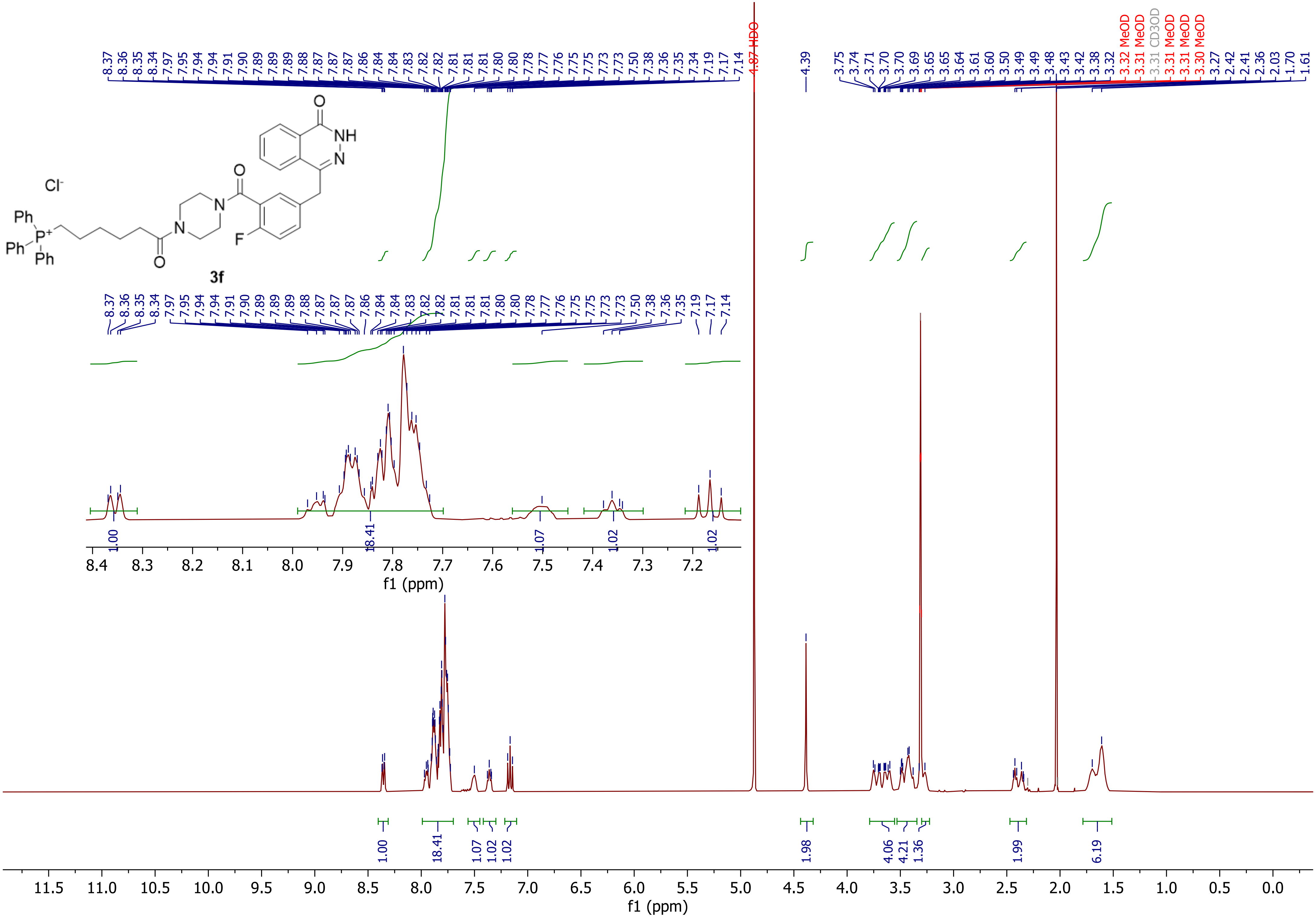

8.37 8.36 8.35 8.34 7.97 7.95 7.94 7.94 7.91 7.90 7.89 7.89 7.89 7.88 7.87 7.87 7.87 7.86 7.84 7.84 7.83 7.82 7.82 7.81 7.81 7.81 7.80 7.80 7.78 7.77 7.76 7.75 7.75 7.73 7.73 7.50 7.38 7.36 7.35 7.34 7.19 7.17 7.14
4.87 HDO
4.39
3.75 3.74 3.71 3.70 3.70 3.69 3.65 3.65 3.64 3.61 3.60 3.50 3.49 3.49 3.48 3.43 3.42 3.38 3.32
3.32 MeOD
3.31 MeOD
3.31 CD3OD
3.31 MeOD
3.30 MeOD
3.27 2.42 2.41 2.36 2.03 1.70 1.61
Cl⁻
Ph
P⁺
O
NH
N
F
3f
1.00
18.41
1.07
1.02
1.02
1.98
4.06
4.21
1.36
1.99
6.19
f1 (ppm)

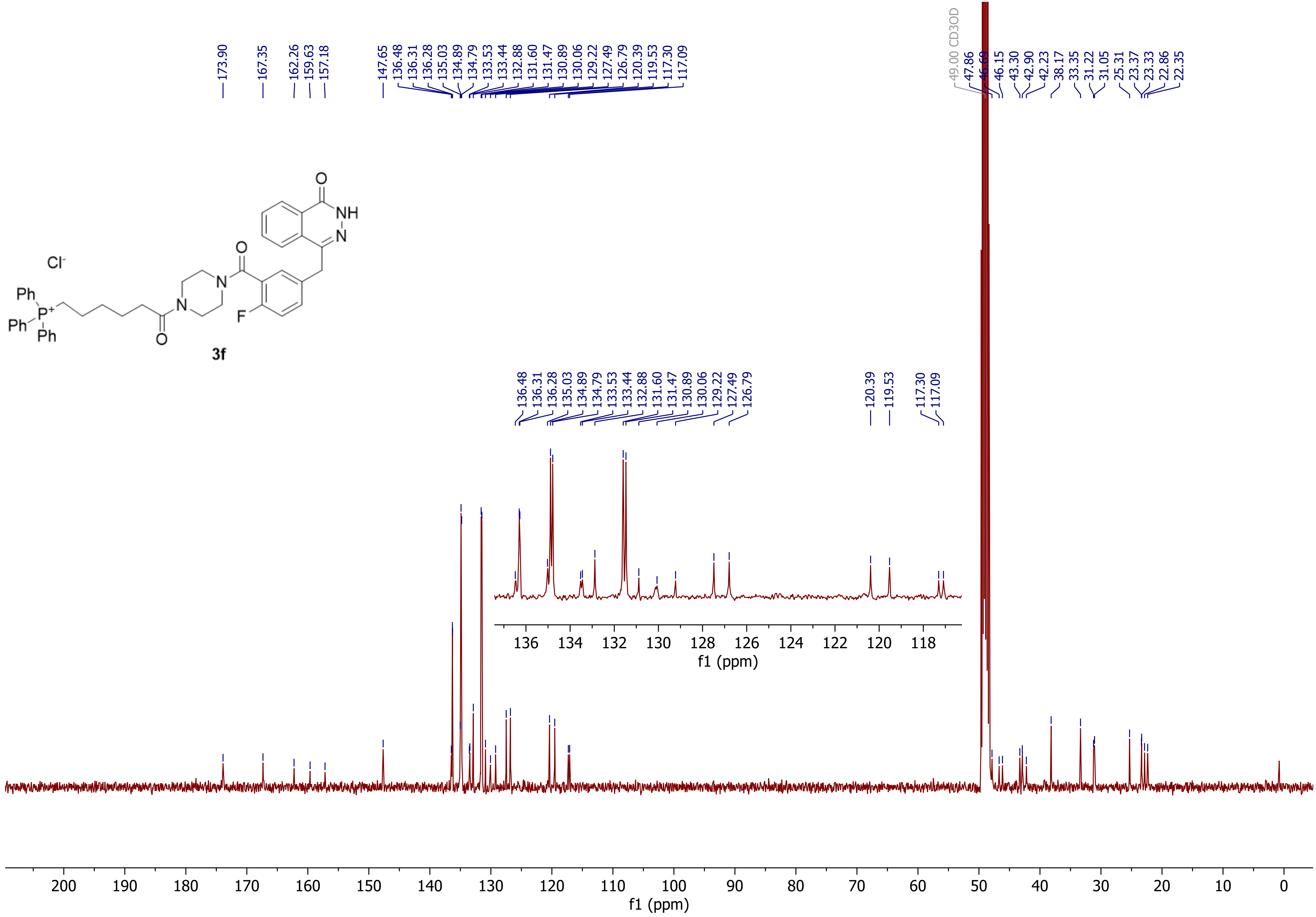
173.90
167.35
162.26
159.63
157.18
147.65
136.48
136.31
136.28
135.03
134.89
134.79
133.53
133.44
132.88
131.60
131.47
130.89
130.06
129.22
127.49
126.79
120.39
119.53
117.30
117.09
49.00 CD3OD
47.86
46.15
43.30
42.90
42.23
38.17
33.35
31.22
31.05
25.31
23.37
23.33
22.86
22.35
Cl⁻
Ph
Ph
Ph
P⁺
N
N
O
O
F
O
NH
N
3f
f1 (ppm)
f1 (ppm)

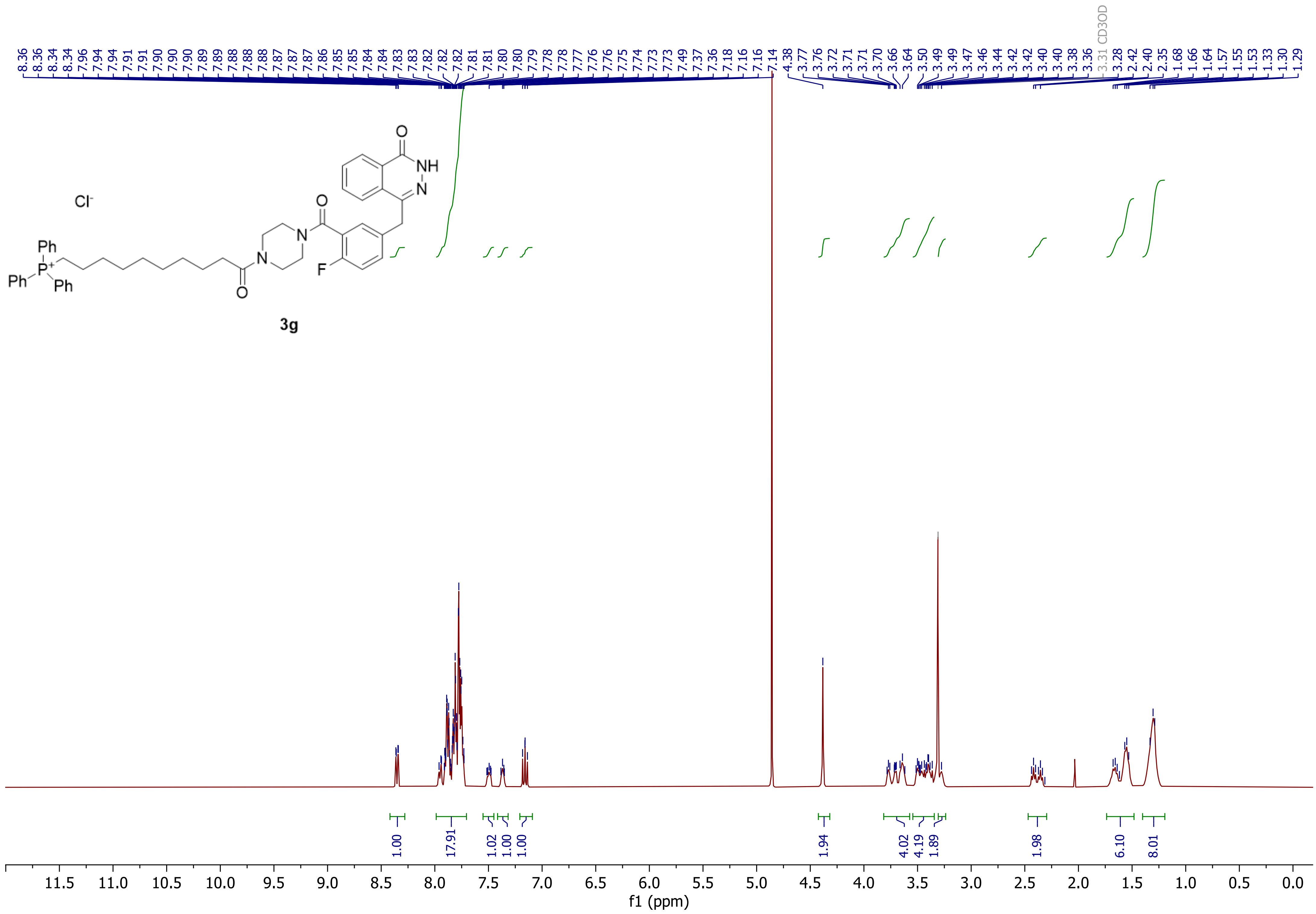
8.36 8.36 8.34 8.34 7.96 7.94 7.94 7.91 7.91 7.90 7.90 7.90 7.89 7.89 7.88 7.88 7.88 7.87 7.87 7.87 7.86 7.85 7.85 7.84 7.84 7.83 7.83 7.82 7.82 7.81 7.81 7.80 7.80 7.79 7.78 7.78 7.77 7.76 7.76 7.75 7.74 7.73 7.73 7.49 7.37 7.36 7.18 7.16 7.14 4.38 3.77 3.76 3.72 3.71 3.71 3.70 3.66 3.64 3.50 3.49 3.47 3.46 3.44 3.42 3.42 3.40 3.40 3.38 3.36 3.31 CD3OD 3.28 2.42 2.40 2.35 1.68 1.66 1.64 1.57 1.55 1.53 1.33 1.30 1.29
O
NH
N
Cl⁻
O
N
N
Ph
P⁺
Ph
Ph
F
O
3g
1.00
17.91
1.02
1.00
1.00
1.94
4.02
4.19
1.89
1.98
6.10
8.01
11.5 11.0 10.5 10.0 9.5 9.0 8.5 8.0 7.5 7.0 6.5 6.0 5.5 5.0 4.5 4.0 3.5 3.0 2.5 2.0 1.5 1.0 0.5 0.0
f1 (ppm)

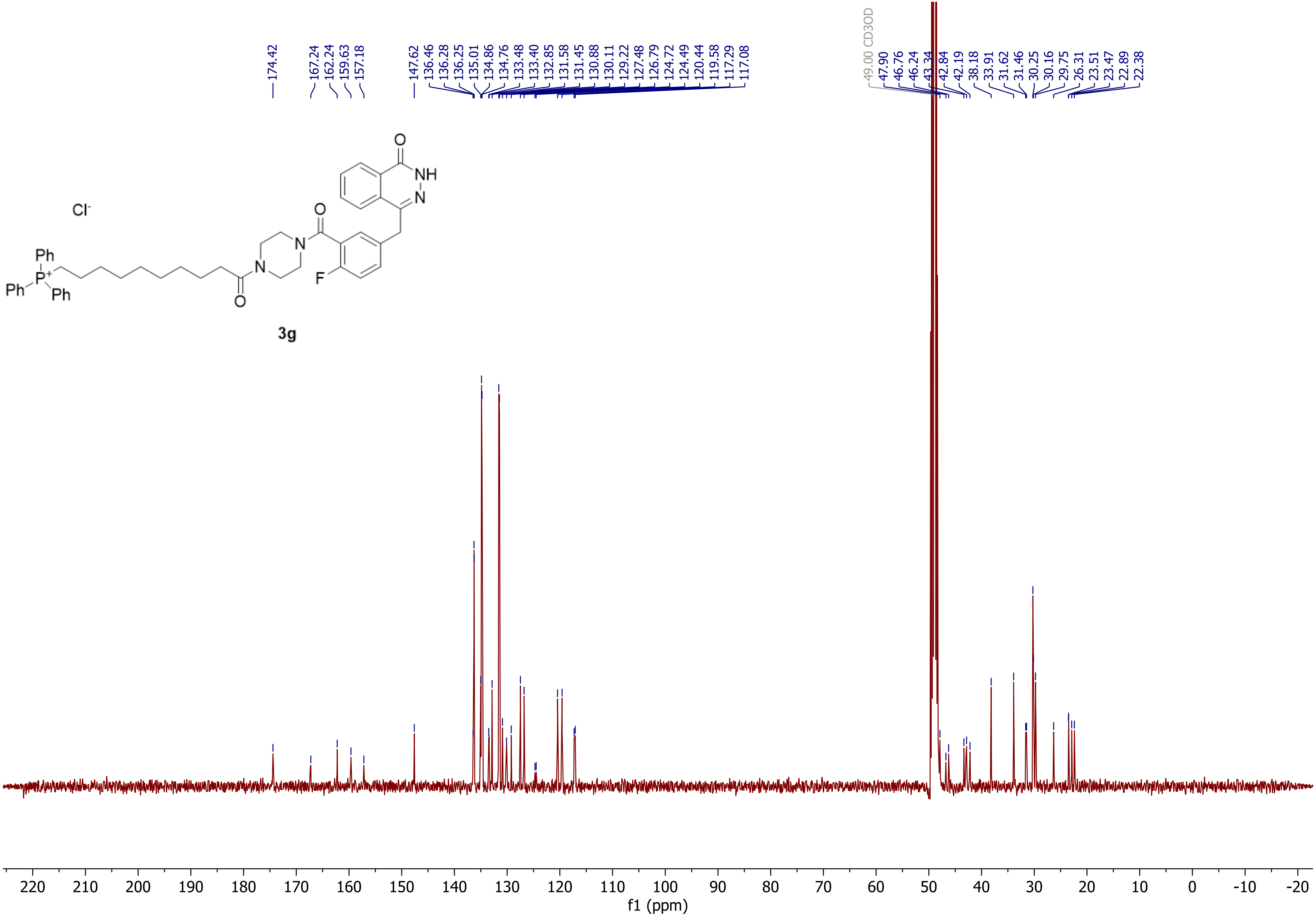
174.42
167.24
162.24
159.63
157.18
147.62
136.46
136.28
136.25
135.01
134.86
134.76
133.48
133.40
132.85
131.58
131.45
130.88
130.11
129.22
127.48
126.79
124.72
124.49
120.44
119.58
117.29
117.08
49.00 CD3OD
47.90
46.76
46.24
43.34
42.84
42.19
38.18
33.91
31.62
31.46
30.25
30.16
29.75
26.31
23.51
23.47
22.89
22.38
Cl⁻
Ph
Ph
Ph
P⁺
N
N
O
O
F
O
NH
N
3g
f1 (ppm)

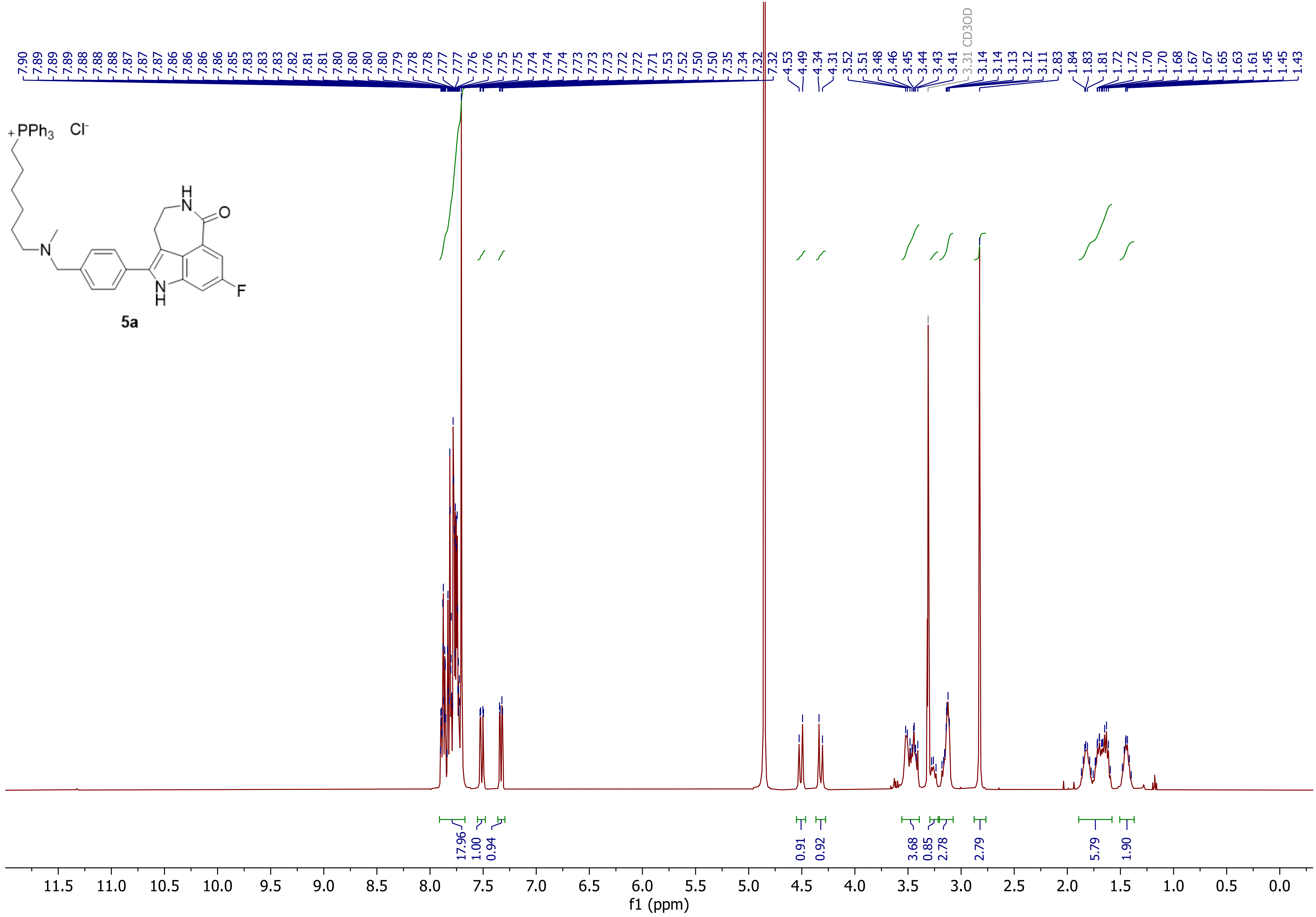

7.90 7.89 7.89 7.89 7.88 7.88 7.88 7.87 7.87 7.87 7.86 7.86 7.86 7.86 7.85 7.83 7.83 7.83 7.82 7.81 7.81 7.80 7.80 7.80 7.79 7.78 7.78 7.77 7.77 7.76 7.76 7.75 7.75 7.74 7.74 7.74 7.73 7.73 7.73 7.72 7.72 7.71 7.53 7.52 7.50 7.50 7.35 7.34 7.32 7.32 4.53 4.49 4.34 4.31 3.52 3.51 3.48 3.46 3.45 3.44 3.43 3.41 3.31 CD3OD 3.14 3.14 3.13 3.12 3.11 2.83 1.84 1.83 1.81 1.72 1.72 1.70 1.70 1.68 1.67 1.67 1.65 1.63 1.61 1.45 1.45 1.43
+PPh3 Cl-
H N O
N H
F
5a
17.96 1.00 0.94 0.91 0.92 3.68 0.85 2.78 2.79 5.79 1.90
11.5 11.0 10.5 10.0 9.5 9.0 8.5 8.0 7.5 7.0 6.5 6.0 5.5 5.0 4.5 4.0 3.5 3.0 2.5 2.0 1.5 1.0 0.5 0.0
f1 (ppm)

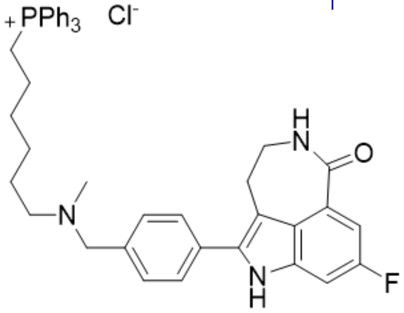

+PPh3 Cl-
H
N
O
N
F
N
H

5a

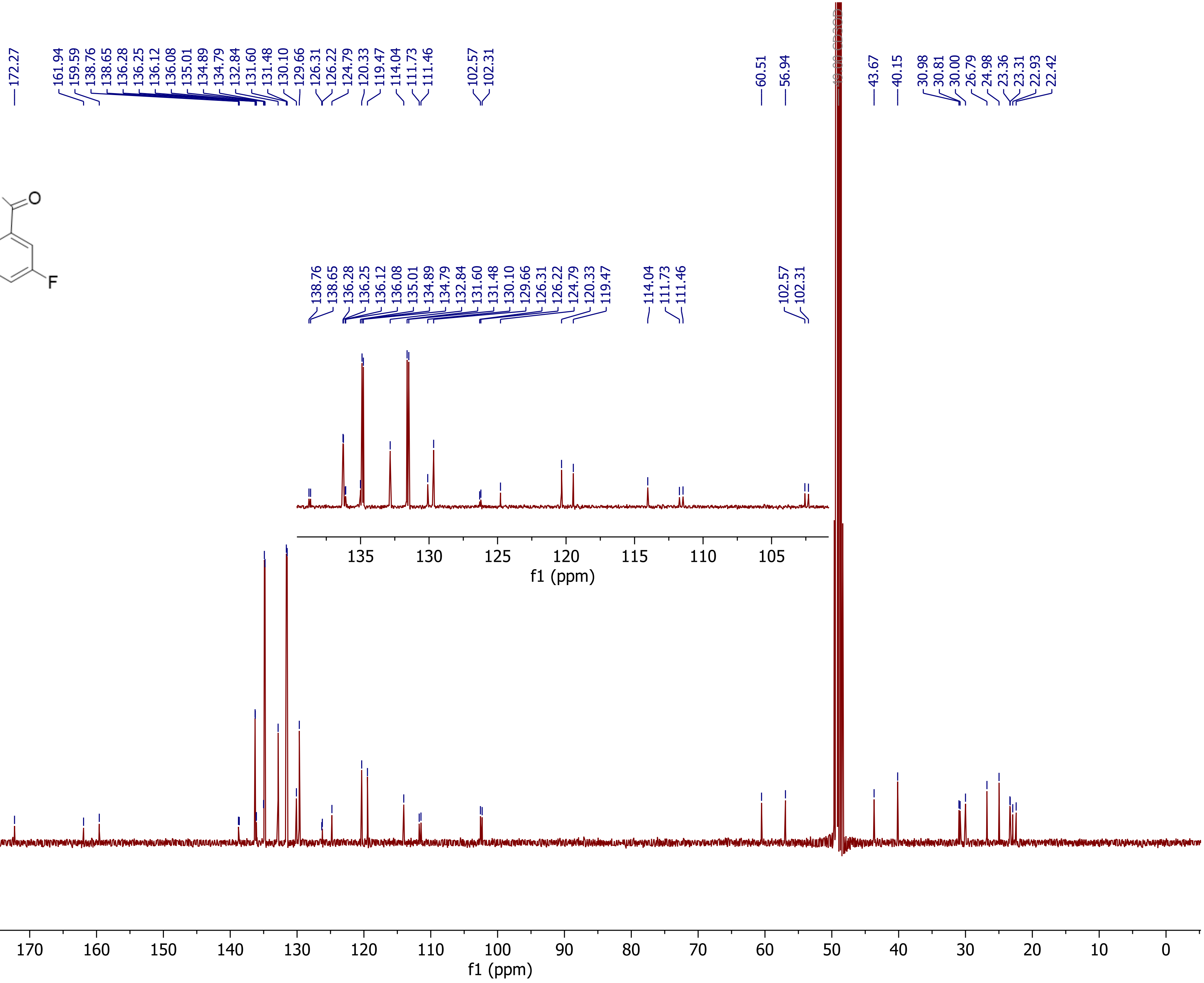

172.27
161.94
159.59
138.76
138.65
136.28
136.25
136.12
136.08
135.01
134.89
134.79
132.84
131.60
131.48
130.10
129.66
126.31
126.22
124.79
120.33
119.47
114.04
111.73
111.46
102.57
102.31
60.51
56.94
43.67
40.15
30.98
30.81
30.00
26.79
24.98
23.36
23.31
22.93
22.42
138.76
138.65
136.28
136.25
136.12
136.08
135.01
134.89
134.79
132.84
131.60
131.48
130.10
129.66
126.31
126.22
124.79
120.33
119.47
114.04
111.73
111.46
102.57
102.31
135 130 125 120 115 110 105
f1 (ppm)
200 190 180 170 160 150 140 130 120 110 100 90 80 70 60 50 40 30 20 10 0
f1 (ppm)

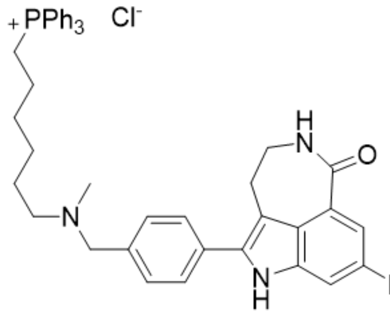

5a

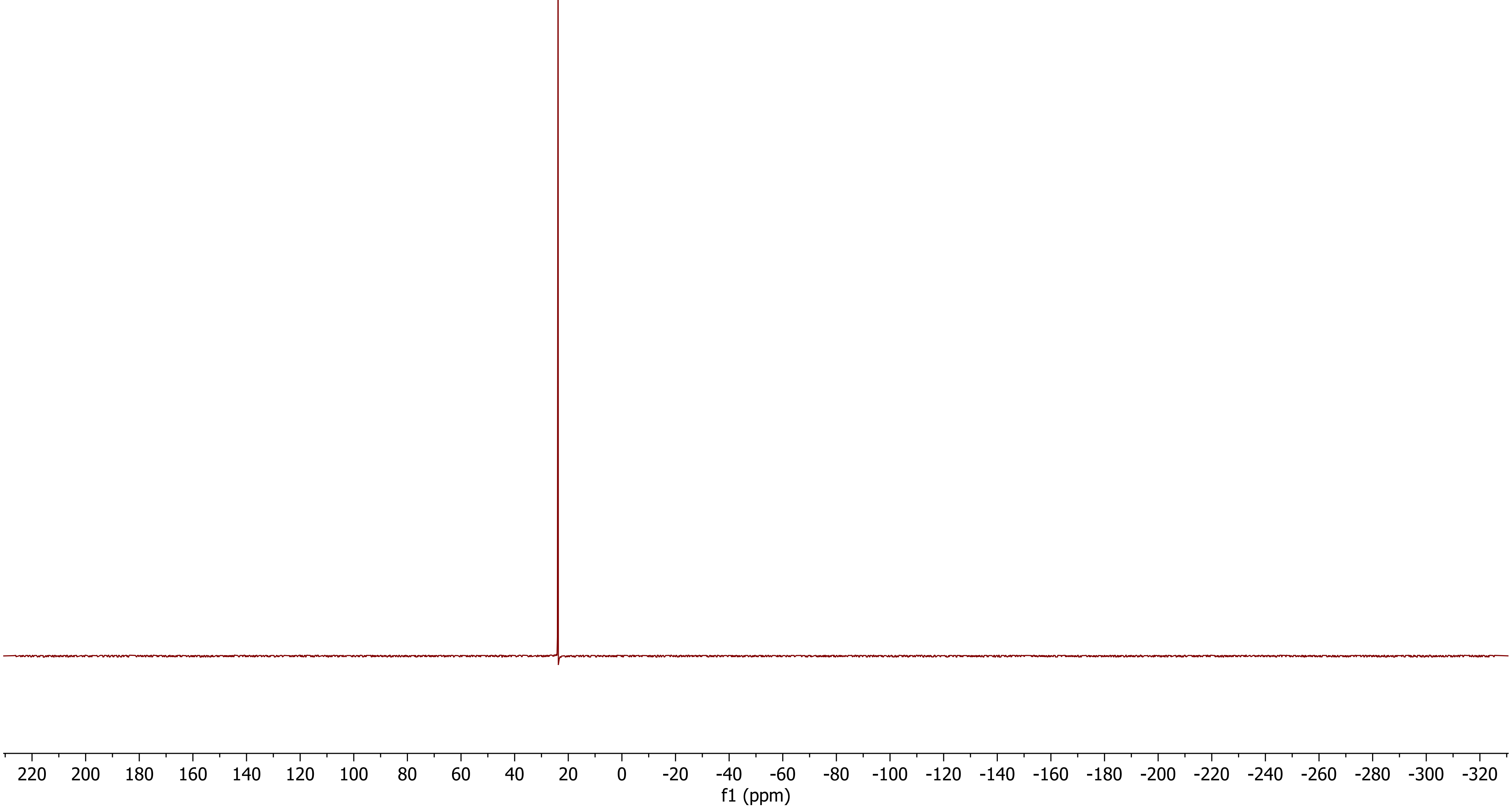

23.79
220
200
180
160
140
120
100
80
60
40
20
0
-20
-40
-60
-80
-100
-120
-140
-160
-180
-200
-220
-240
-260
-280
-300
-320
f1 (ppm)

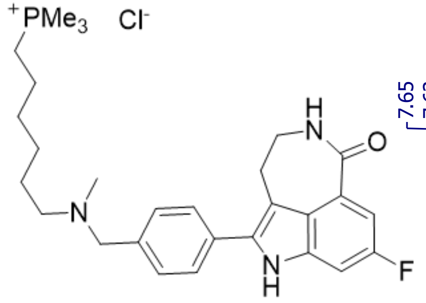

5a

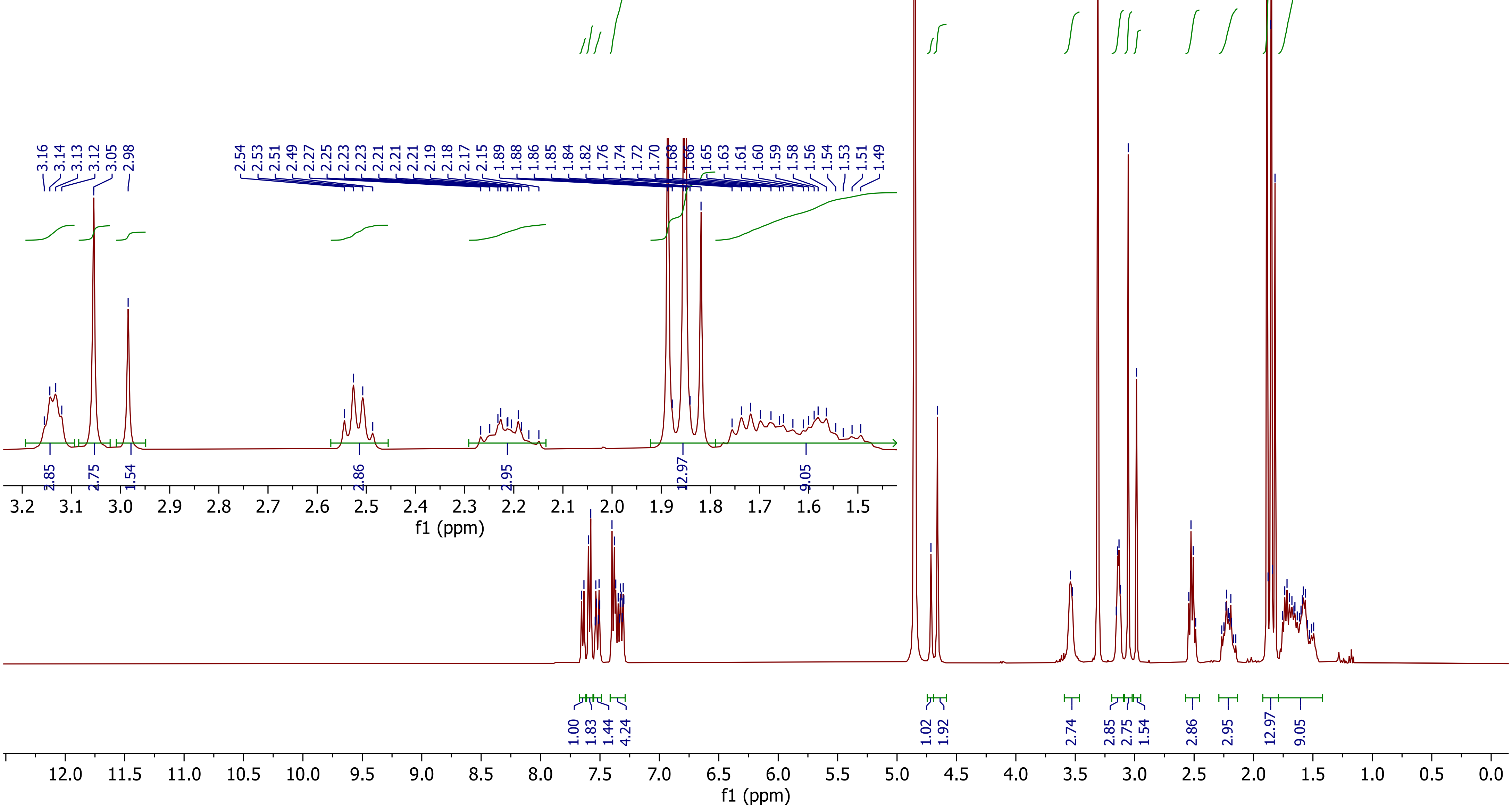

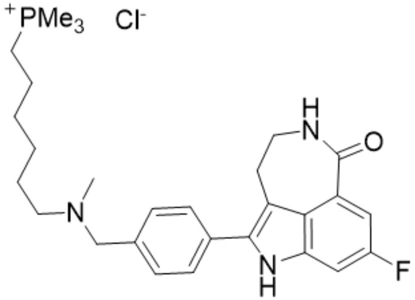

+PMe3 Cl-
H
N
O
F
N
H

5a

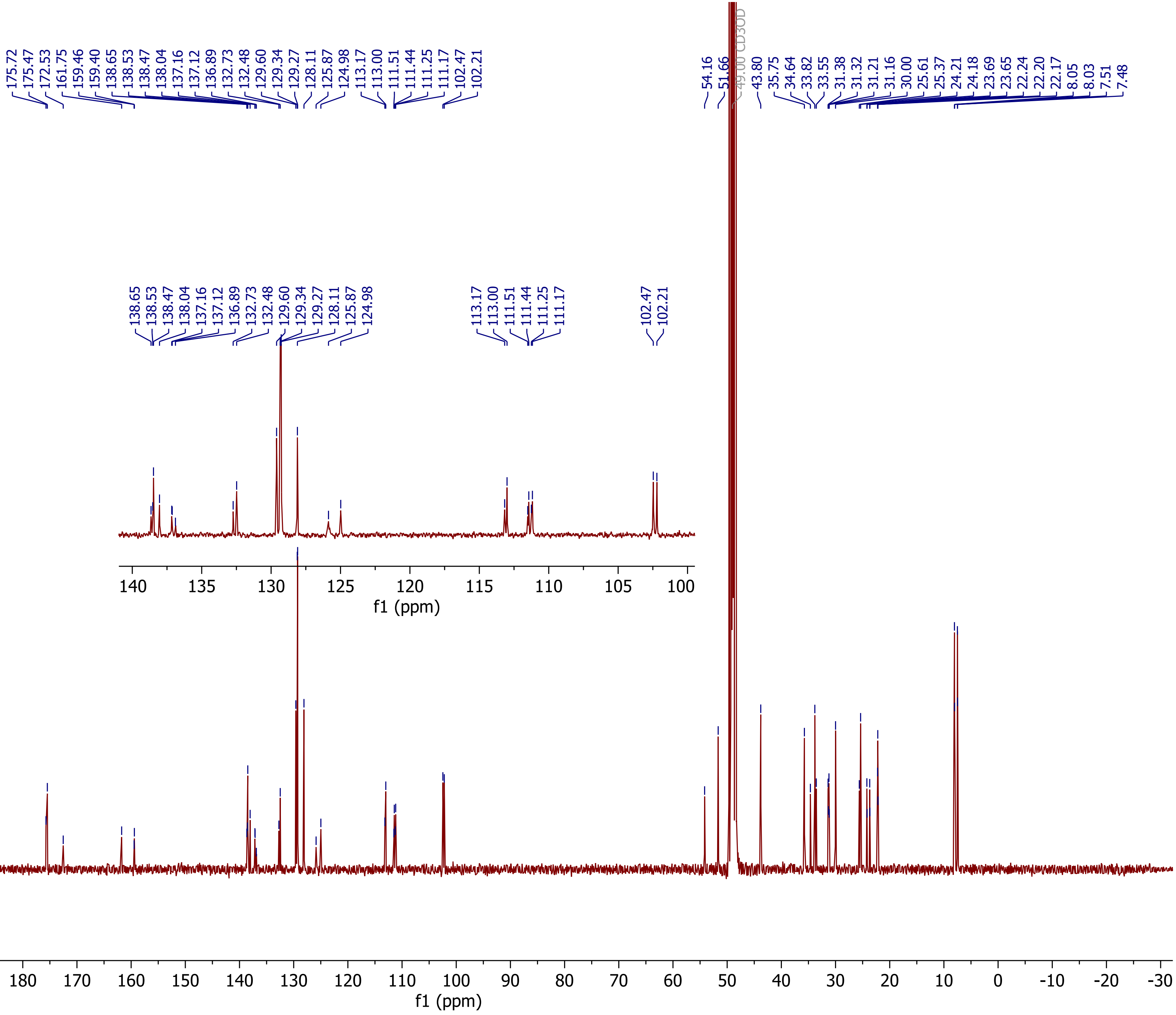

175.72
175.47
172.53
161.75
159.46
159.40
138.65
138.53
138.47
138.04
137.16
137.12
136.89
132.73
132.48
129.60
129.34
129.27
128.11
125.87
124.98
113.17
113.00
111.51
111.44
111.25
111.17
102.47
102.21
54.16
51.66
49.00 CD3OD
43.80
35.75
34.64
33.82
33.55
31.38
31.32
31.21
31.16
30.00
25.61
25.37
24.21
24.18
23.69
23.65
22.24
22.20
22.17
8.05
8.03
7.51
7.48
140 135 130 125 120 115 110 105 100
f1 (ppm)
230 220 210 200 190 180 170 160 150 140 130 120 110 100 90 80 70 60 50 40 30 20 10 0 -10 -20 -30
f1 (ppm)

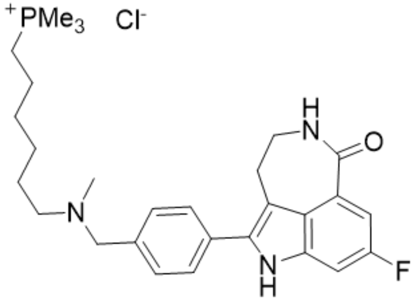
+PMe3 Cl-
H
N
O
N
F
N
H

5a

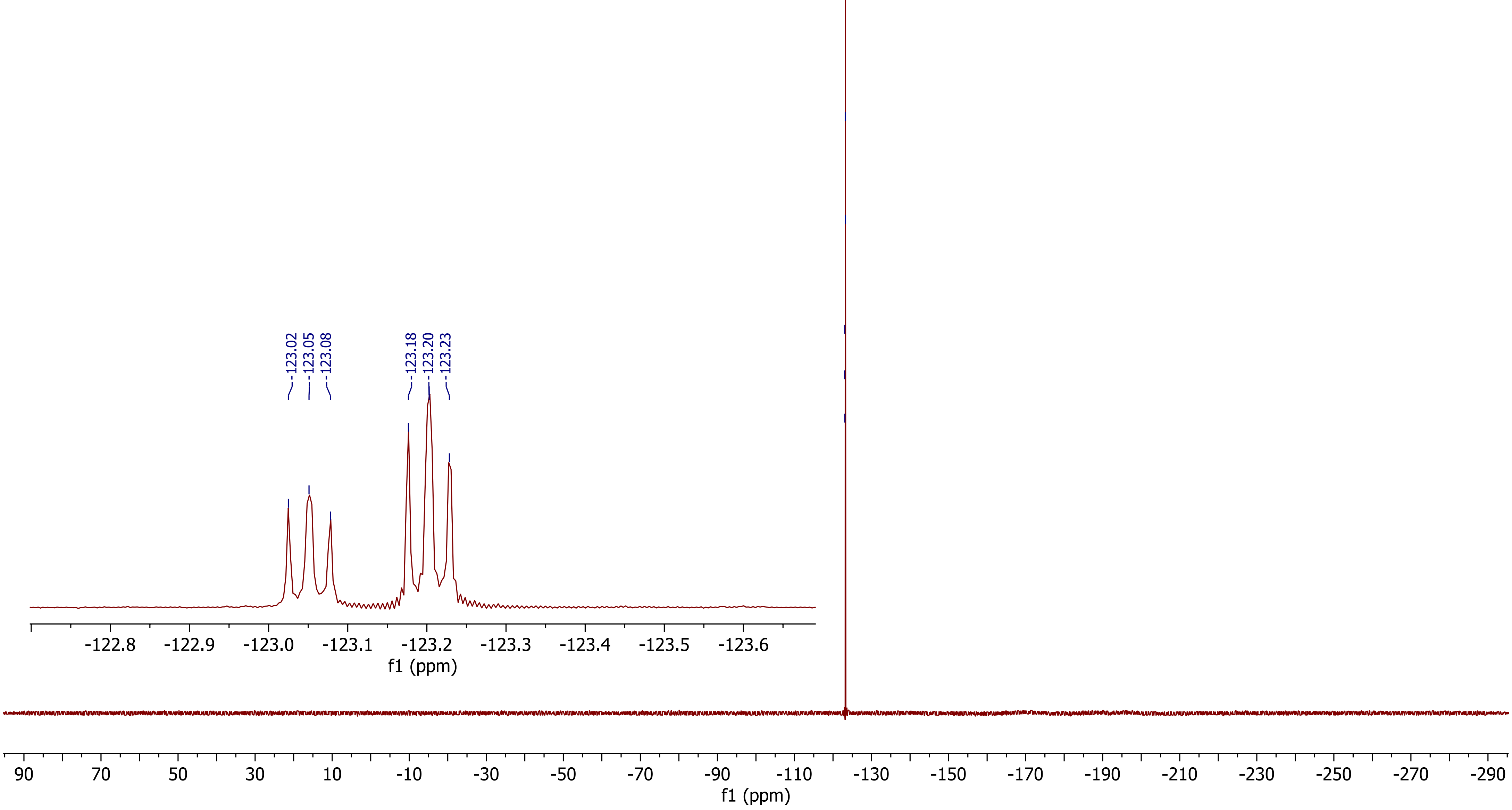
-123.02
-123.05
-123.08
-123.18
-123.20
-123.23
-122.8
-122.9
-123.0
-123.1
-123.2
-123.3
-123.4
-123.5
-123.6
f1 (ppm)
90
70
50
30
10
-10
-30
-50
-70
-90
-110
-130
-150
-170
-190
-210
-230
-250
-270
-290
f1 (ppm)

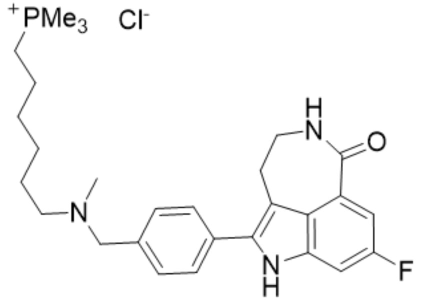
+PMe3 Cl-
H
N
O
N
N
H
F

5a

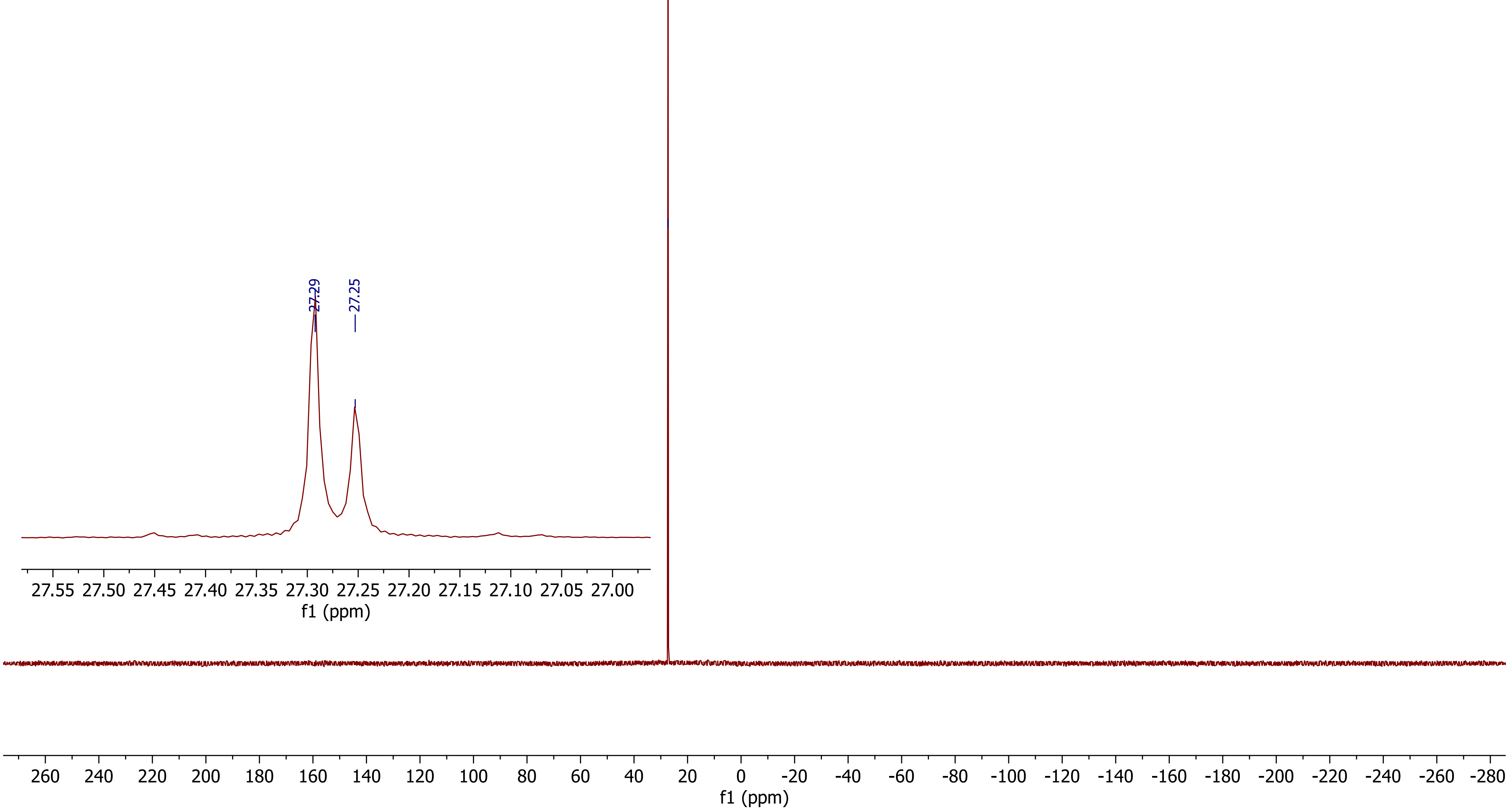
27.29
27.25
27.29
27.25
27.55 27.50 27.45 27.40 27.35 27.30 27.25 27.20 27.15 27.10 27.05 27.00
f1 (ppm)
260 240 220 200 180 160 140 120 100 80 60 40 20 0 -20 -40 -60 -80 -100 -120 -140 -160 -180 -200 -220 -240 -260 -280
f1 (ppm)

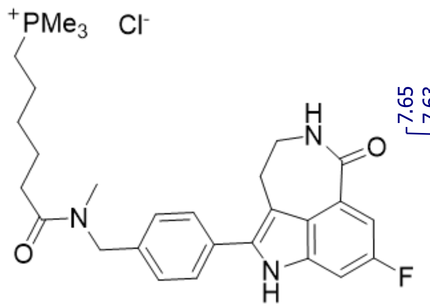

+PMe3 Cl-
H
N
O
N
O
N
H
F

6a

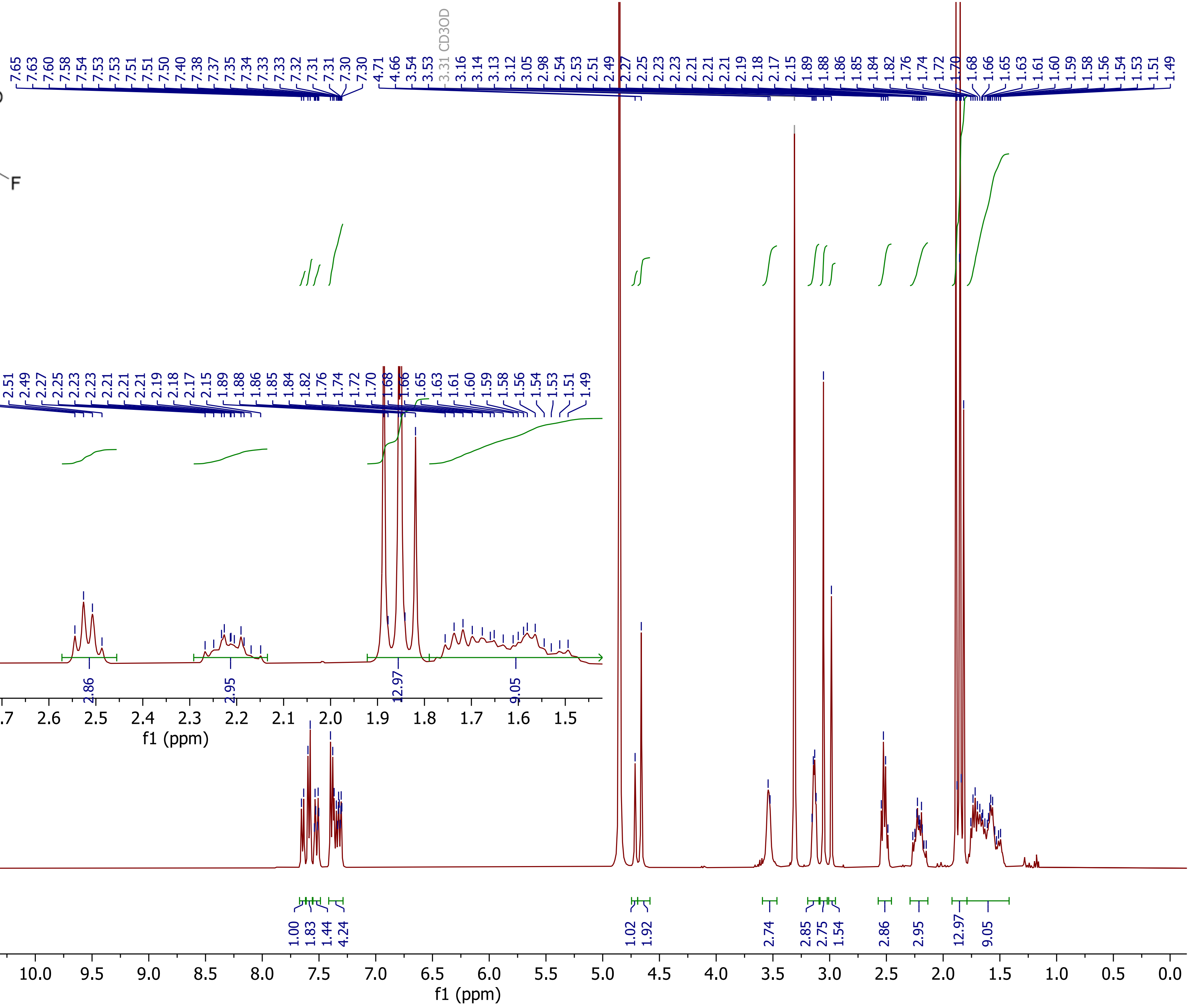

7.65 7.63 7.60 7.58 7.54 7.53 7.53 7.51 7.51 7.50 7.40 7.38 7.37 7.35 7.34 7.33 7.33 7.32 7.31 7.31 7.30 7.30 4.71 4.66 3.54 3.53 3.31 CD3OD 3.16 3.14 3.13 3.12 3.05 2.98 2.54 2.53 2.51 2.49 2.27 2.25 2.23 2.23 2.21 2.21 2.21 2.19 2.18 2.17 2.15 1.89 1.88 1.86 1.85 1.84 1.82 1.76 1.74 1.72 1.70 1.68 1.66 1.65 1.63 1.61 1.60 1.59 1.58 1.56 1.54 1.53 1.51 1.49
3.16 3.14 3.13 3.12 3.05 2.98
2.54 2.53 2.51 2.49 2.27 2.25 2.23 2.23 2.21 2.21 2.21 2.19 2.18 2.17 2.15 1.89 1.88 1.86 1.85 1.84 1.82 1.76 1.74 1.72 1.70 1.68 1.66 1.65 1.63 1.61 1.60 1.59 1.58 1.56 1.54 1.53 1.51 1.49
2.85 2.75 1.54 2.86 2.95 12.97 9.05
3.2 3.1 3.0 2.9 2.8 2.7 2.6 2.5 2.4 2.3 2.2 2.1 2.0 1.9 1.8 1.7 1.6 1.5
f1 (ppm)
1.00 1.83 1.44 4.24 1.02 1.92 2.74 2.85 2.75 1.54 2.86 2.95 12.97 9.05
12.0 11.5 11.0 10.5 10.0 9.5 9.0 8.5 8.0 7.5 7.0 6.5 6.0 5.5 5.0 4.5 4.0 3.5 3.0 2.5 2.0 1.5 1.0 0.5 0.0
f1 (ppm)

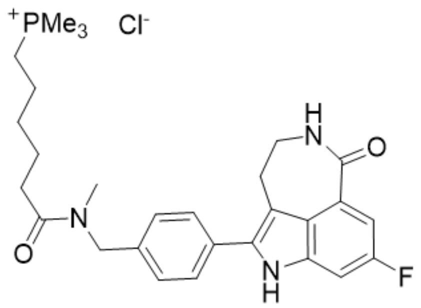
+PMe3 Cl-
H
N
O
O
N
N
H
F

6a

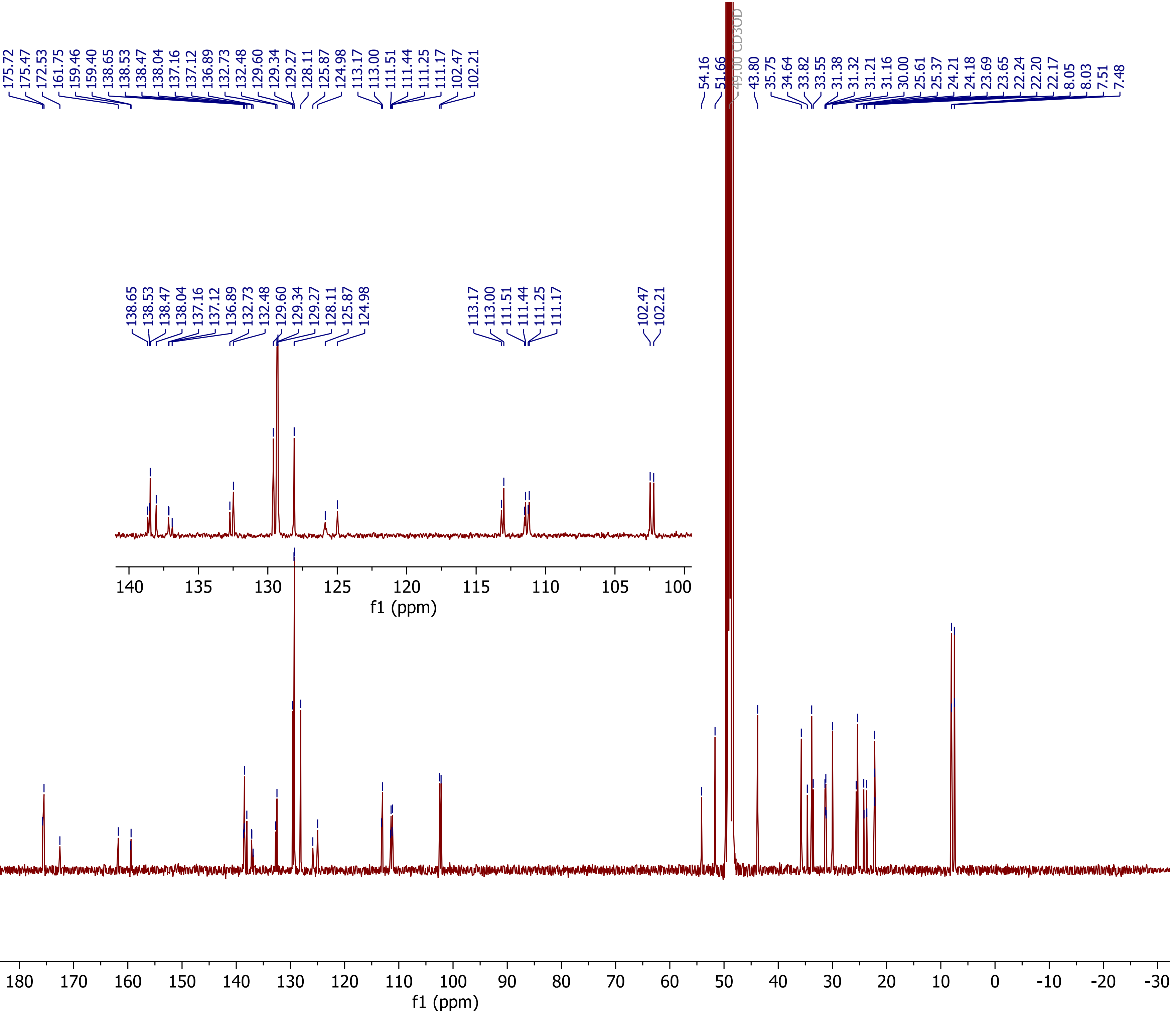
175.72
175.47
172.53
161.75
159.46
159.40
138.65
138.53
138.47
138.04
137.16
137.12
136.89
132.73
132.48
129.60
129.34
129.27
128.11
125.87
124.98
113.17
113.00
111.51
111.44
111.25
111.17
102.47
102.21
54.16
51.66
49.00 CD3OD
43.80
35.75
34.64
33.82
33.55
31.38
31.32
31.21
31.16
30.00
25.61
25.37
24.21
24.18
23.69
23.65
22.24
22.20
22.17
8.05
8.03
7.51
7.48
f1 (ppm)
f1 (ppm)

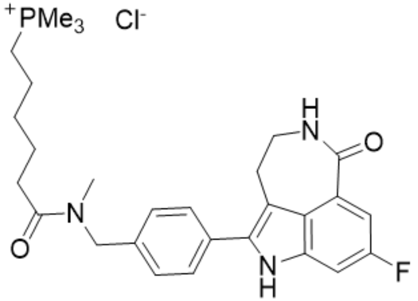

+PMe3 Cl-
H
N
O
O
N
N
H
F

6a

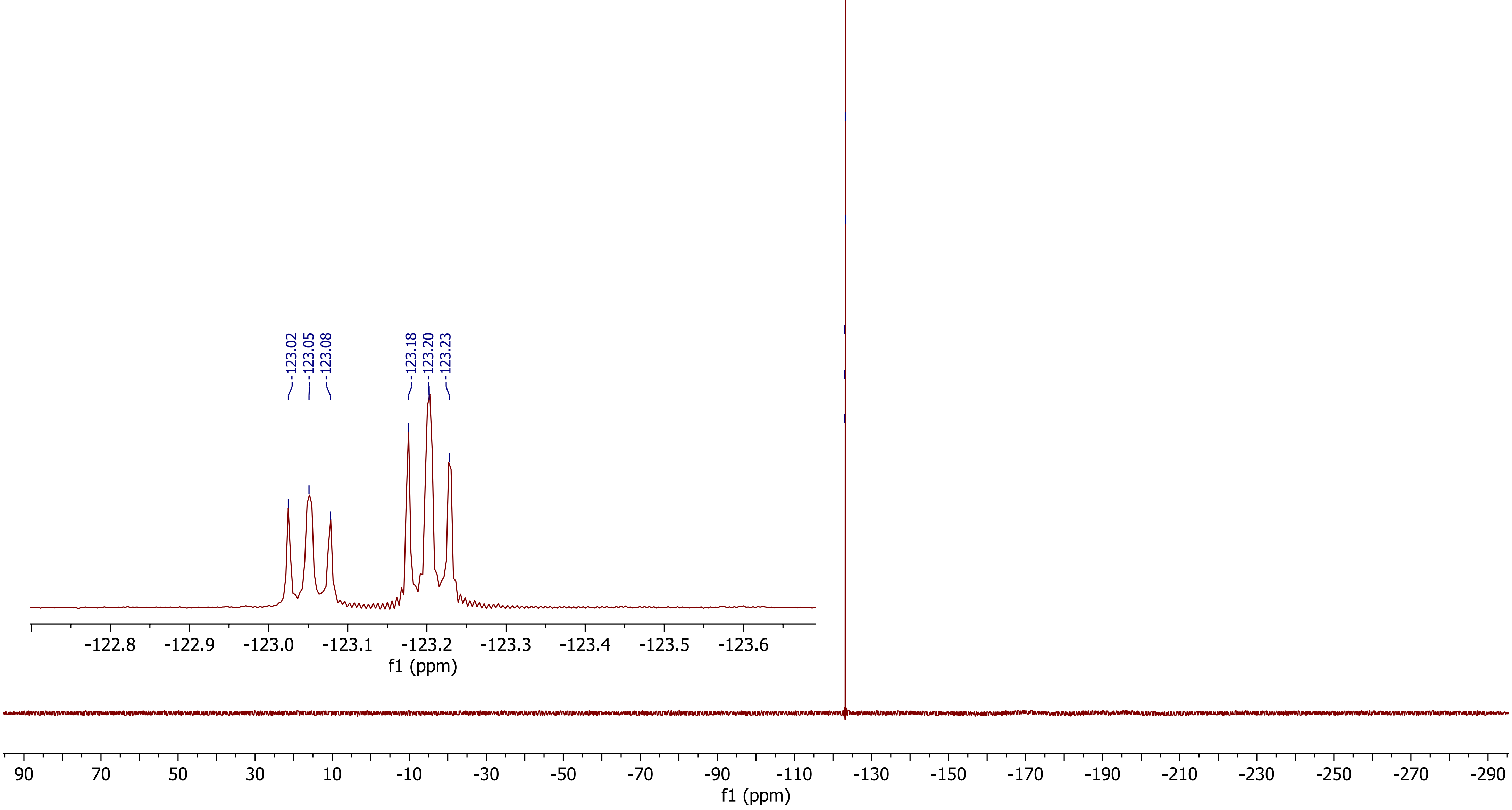

-123.02
-123.05
-123.08
-123.18
-123.20
-123.23
-122.8 -122.9 -123.0 -123.1 -123.2 -123.3 -123.4 -123.5 -123.6
f1 (ppm)
90 70 50 30 10 -10 -30 -50 -70 -90 -110 -130 -150 -170 -190 -210 -230 -250 -270 -290
f1 (ppm)

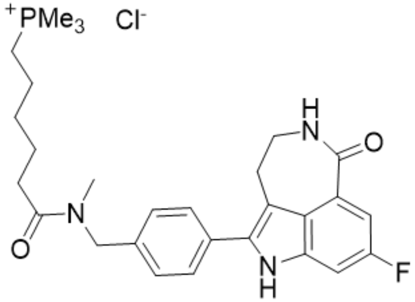
+PMe3 Cl-
H
N
O
N
O
N
H
F

6a

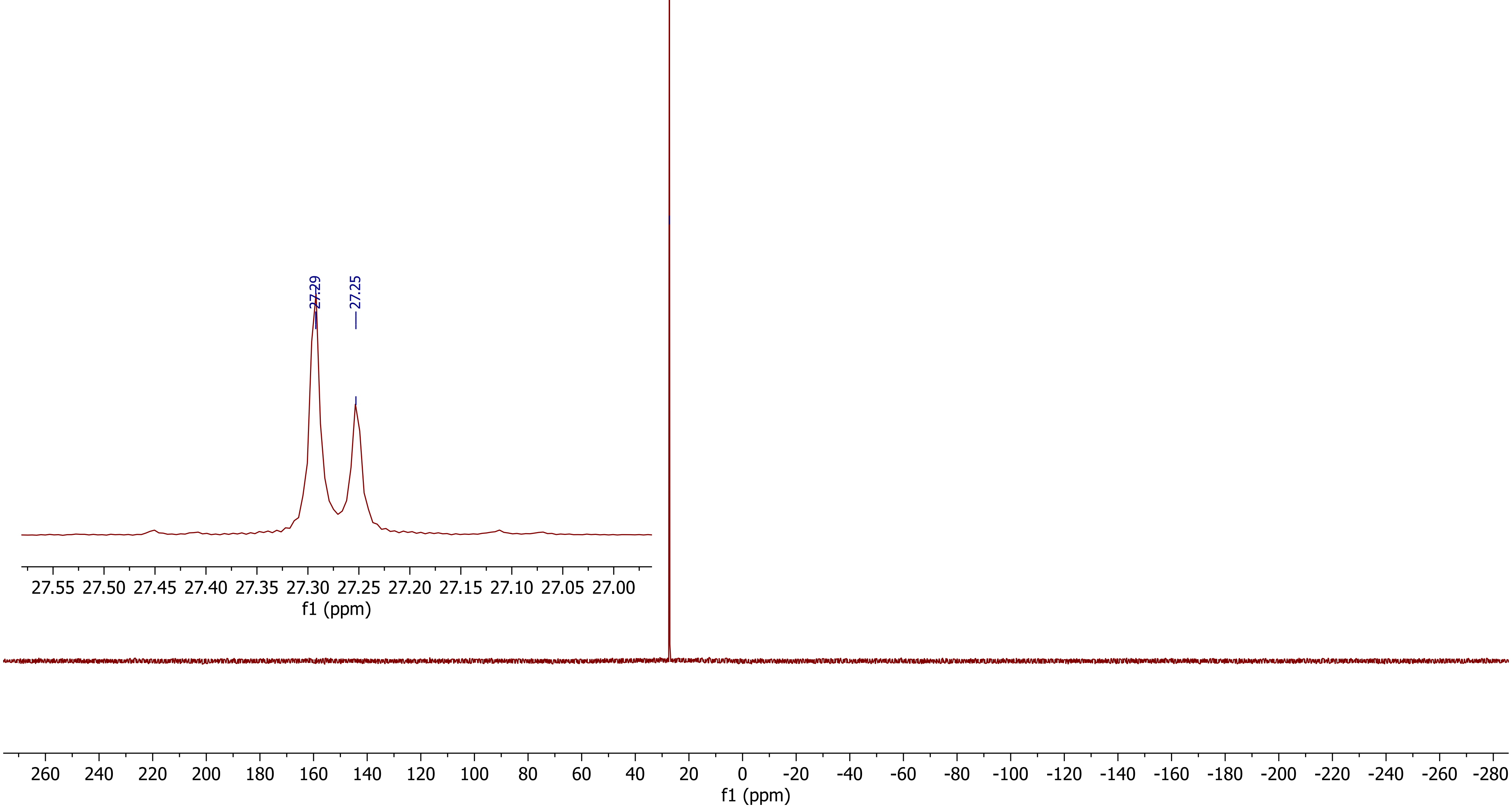
27.29
27.25
27.55 27.50 27.45 27.40 27.35 27.30 27.25 27.20 27.15 27.10 27.05 27.00
f1 (ppm)
260 240 220 200 180 160 140 120 100 80 60 40 20 0 -20 -40 -60 -80 -100 -120 -140 -160 -180 -200 -220 -240 -260 -280
f1 (ppm)

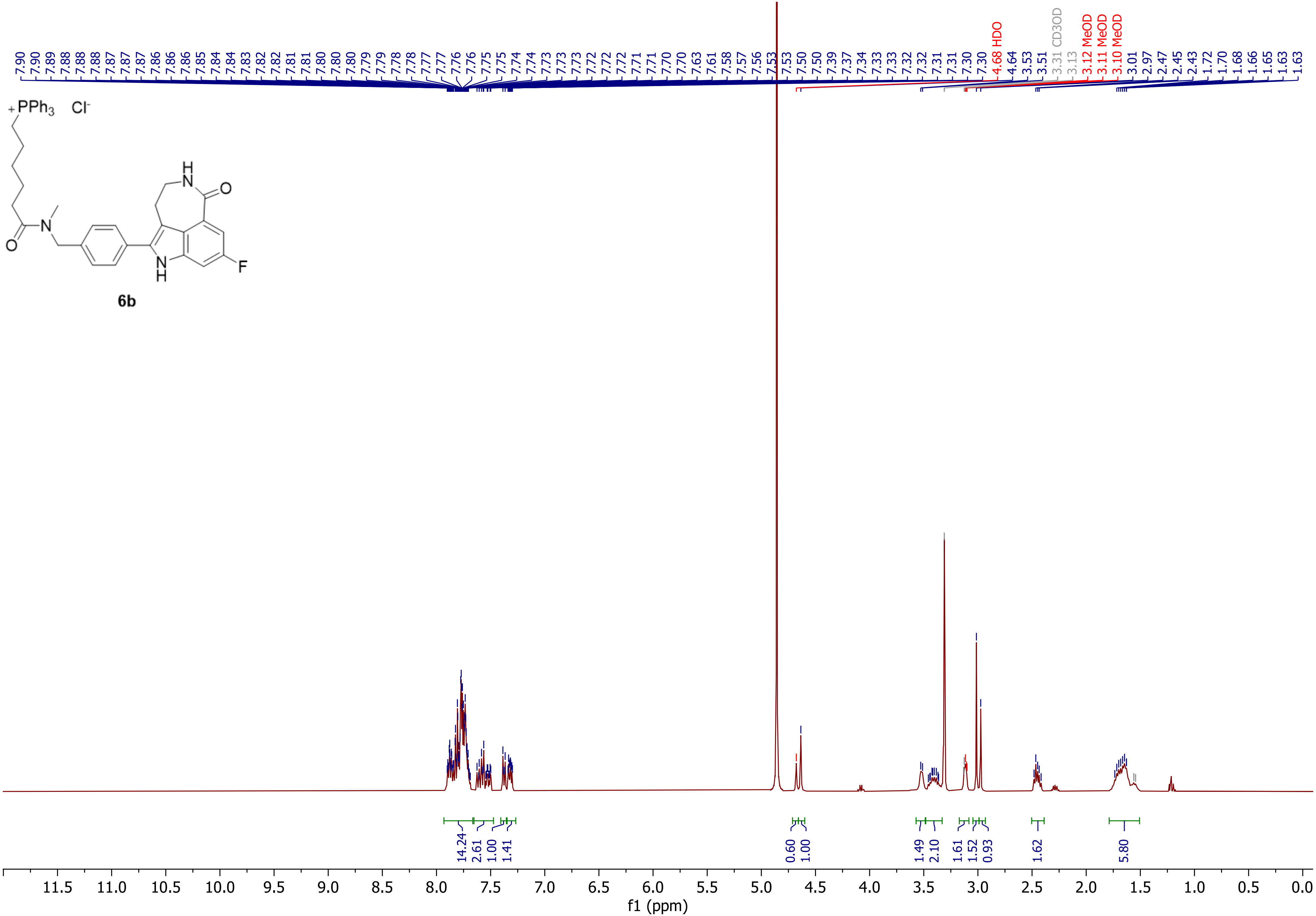
+PPh3 Cl-
N
O
H
N
O
N
H
F

6b

7.90
7.90
7.89
7.88
7.88
7.88
7.87
7.87
7.86
7.86
7.86
7.85
7.84
7.84
7.83
7.82
7.82
7.81
7.81
7.80
7.80
7.80
7.79
7.78
7.78
7.77
7.77
7.76
7.76
7.75
7.75
7.74
7.74
7.73
7.73
7.73
7.72
7.72
7.72
7.71
7.71
7.70
7.70
7.63
7.61
7.58
7.57
7.56
7.53
7.53
7.50
7.50
7.39
7.37
7.34
7.33
7.33
7.32
7.32
7.31
7.31
7.30
7.30
4.68 HDO
4.64
3.53
3.51
3.31 CD3OD
3.13
3.12 MeOD
3.11 MeOD
3.10 MeOD
3.01
2.97
2.47
2.45
2.43
1.72
1.70
1.68
1.66
1.65
1.63
1.63
14.24
2.61
1.00
1.41
0.60
1.00
1.49
2.10
1.61
1.52
0.93
1.62
5.80
11.5 11.0 10.5 10.0 9.5 9.0 8.5 8.0 7.5 7.0 6.5 6.0 5.5 5.0 4.5 4.0 3.5 3.0 2.5 2.0 1.5 1.0 0.5 0.0
f1 (ppm)

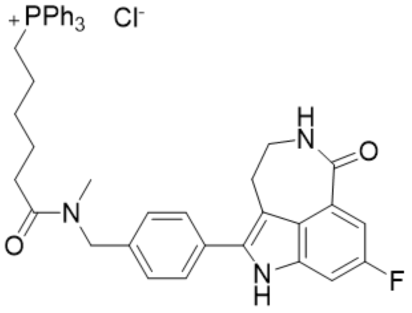

+PPh3 Cl-
H
N
O
O
N
N
H
F

6b

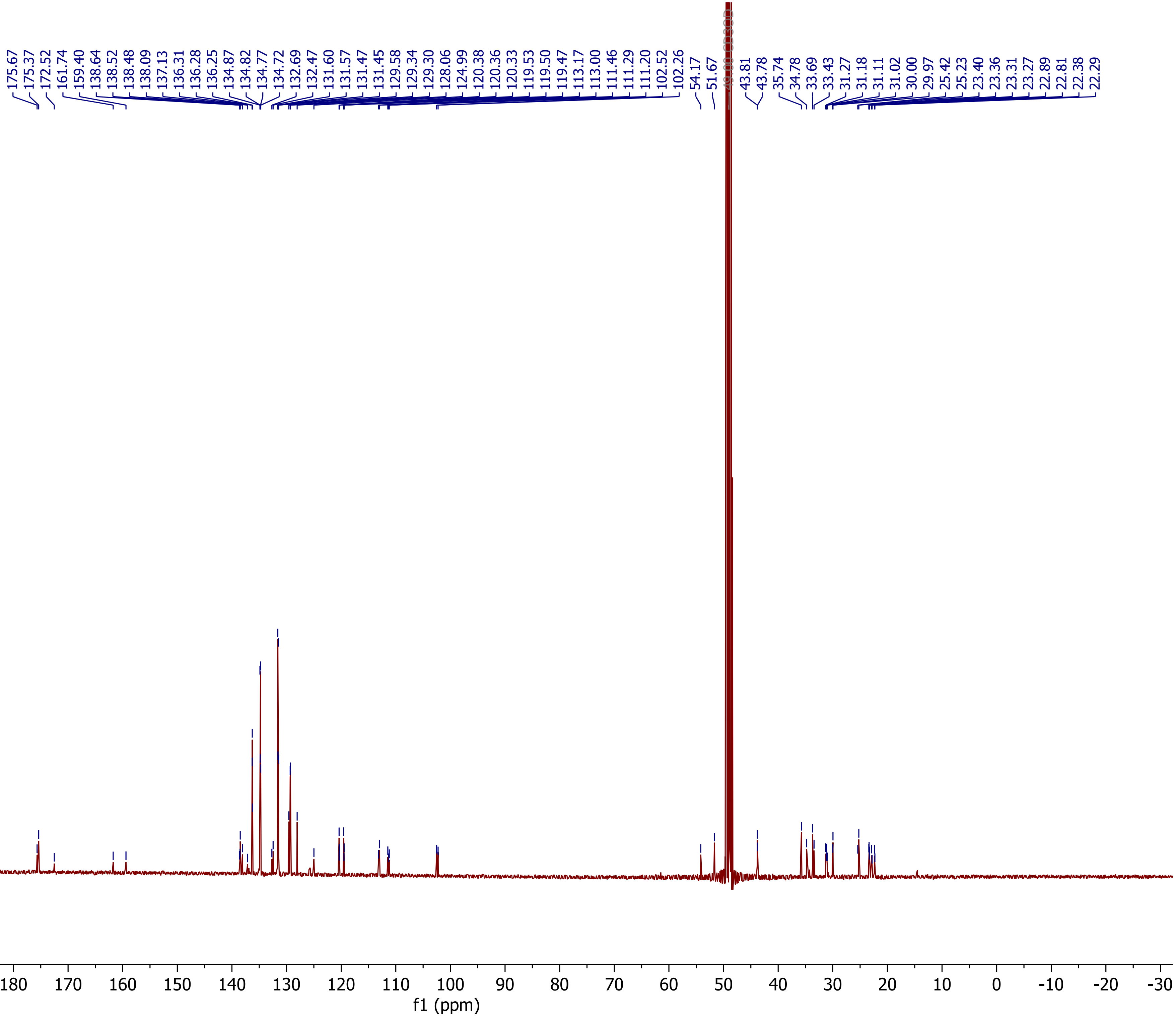

175.67
175.37
172.52
161.74
159.40
138.64
138.52
138.48
138.09
137.13
136.31
136.28
136.25
134.87
134.82
134.77
134.72
132.69
132.47
131.60
131.57
131.47
131.45
129.58
129.34
129.30
128.06
124.99
120.38
120.36
120.33
119.53
119.50
119.47
113.17
113.00
111.46
111.29
111.20
102.52
102.26
54.17
51.67
43.81
43.78
35.74
34.78
33.69
33.43
31.27
31.18
31.11
31.02
30.00
29.97
25.42
25.23
23.40
23.36
23.31
23.27
22.89
22.81
22.38
22.29
230 220 210 200 190 180 170 160 150 140 130 120 110 100 90 80 70 60 50 40 30 20 10 0 -10 -20 -30
f1 (ppm)

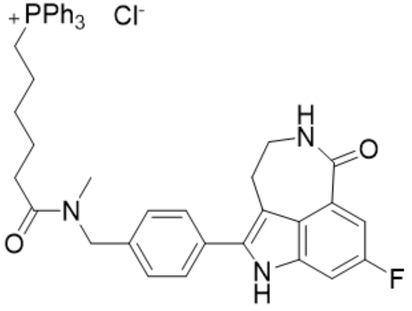
+PPh3 Cl-
H
N
O
N
O
N
H
F

6b

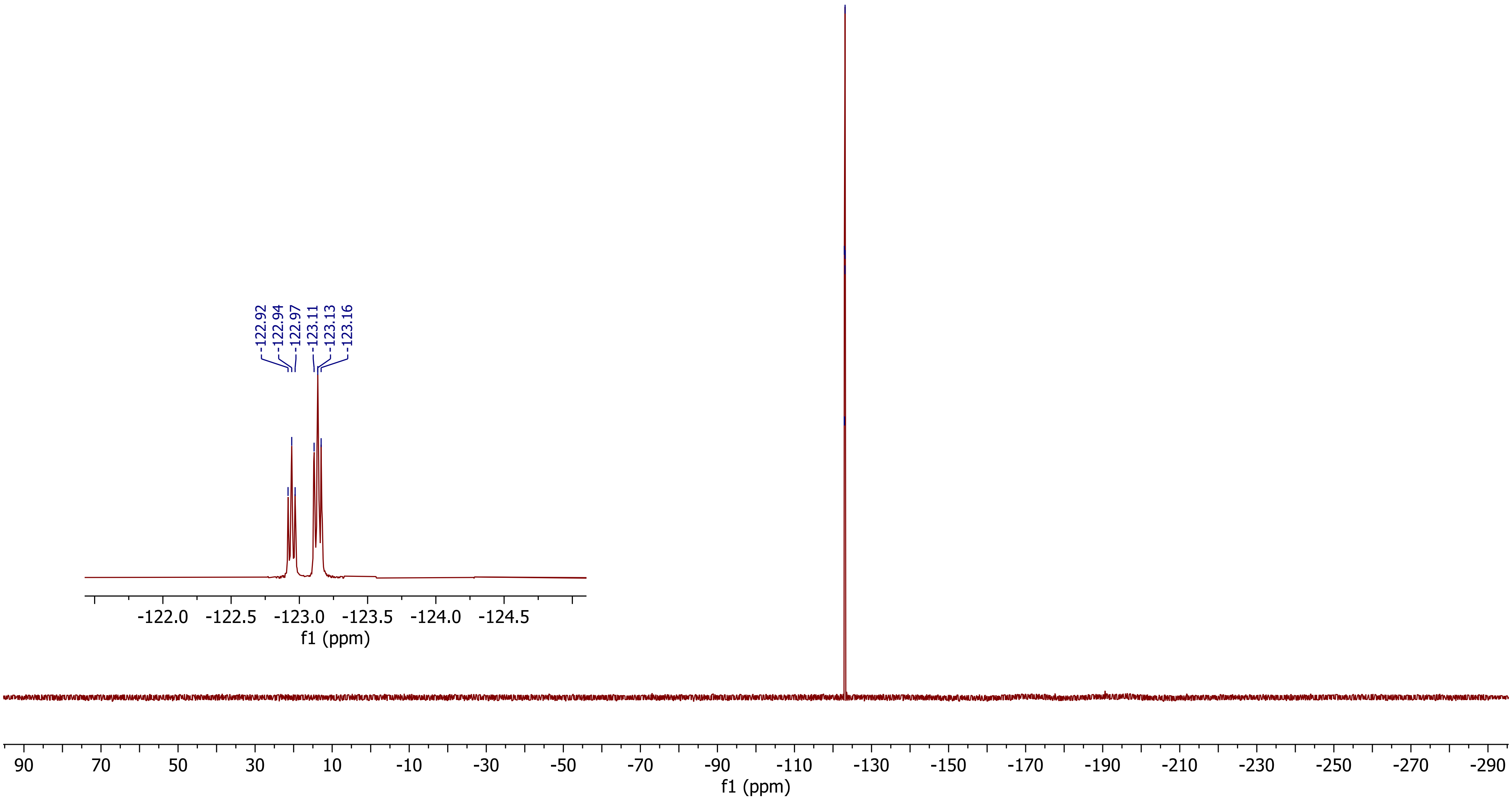
-122.92
-122.94
-122.97
-123.11
-123.13
-123.16
-122.0 -122.5 -123.0 -123.5 -124.0 -124.5
f1 (ppm)
90 70 50 30 10 -10 -30 -50 -70 -90 -110 -130 -150 -170 -190 -210 -230 -250 -270 -290
f1 (ppm)

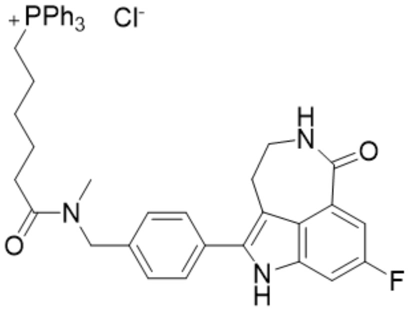
+PPh3 Cl-
H
N
O
N
O
N
H
F

6b

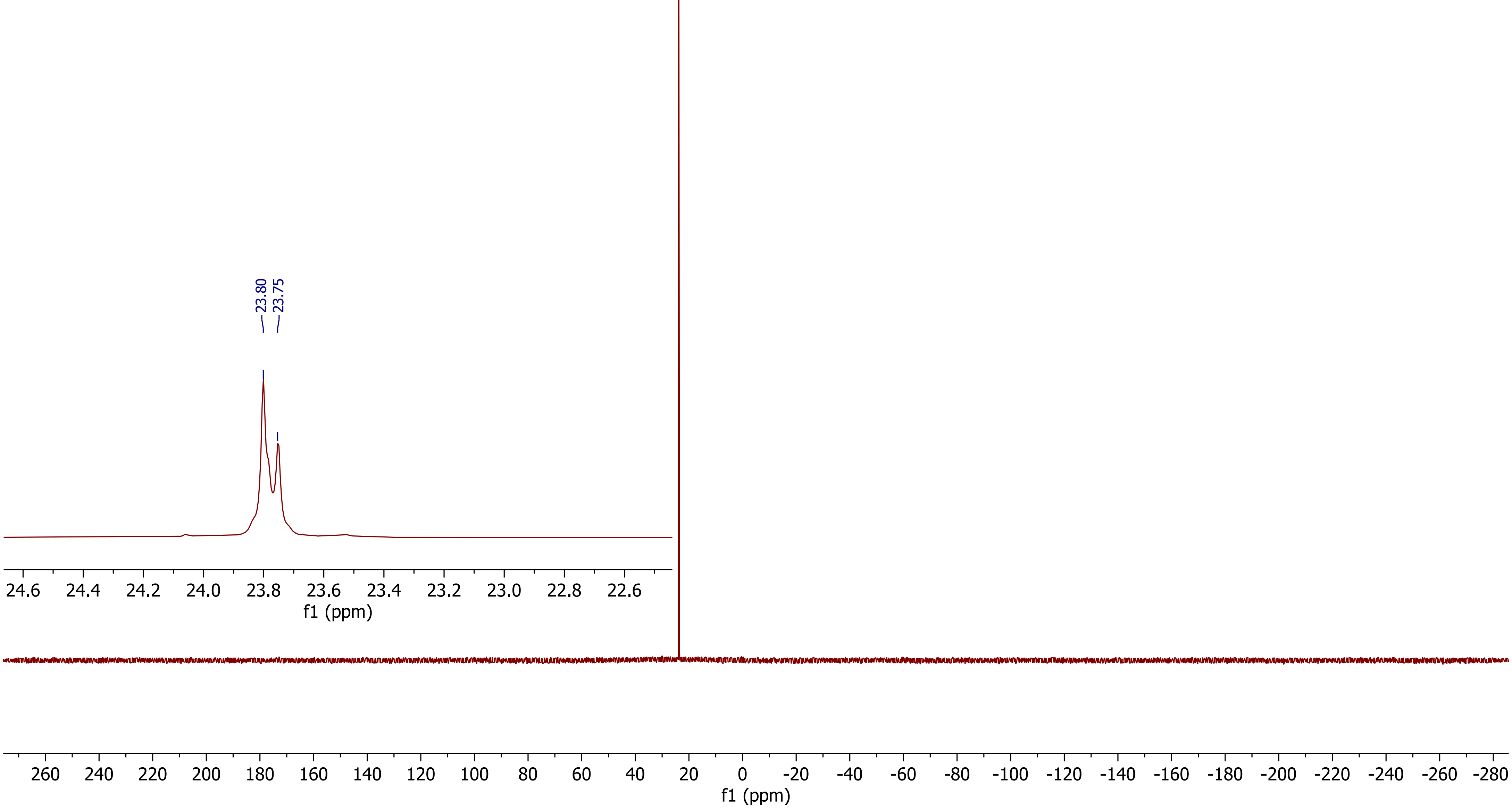
23.80
23.75
24.6 24.4 24.2 24.0 23.8 23.6 23.4 23.2 23.0 22.8 22.6
f1 (ppm)
260 240 220 200 180 160 140 120 100 80 60 40 20 0 -20 -40 -60 -80 -100 -120 -140 -160 -180 -200 -220 -240 -260 -280
f1 (ppm)

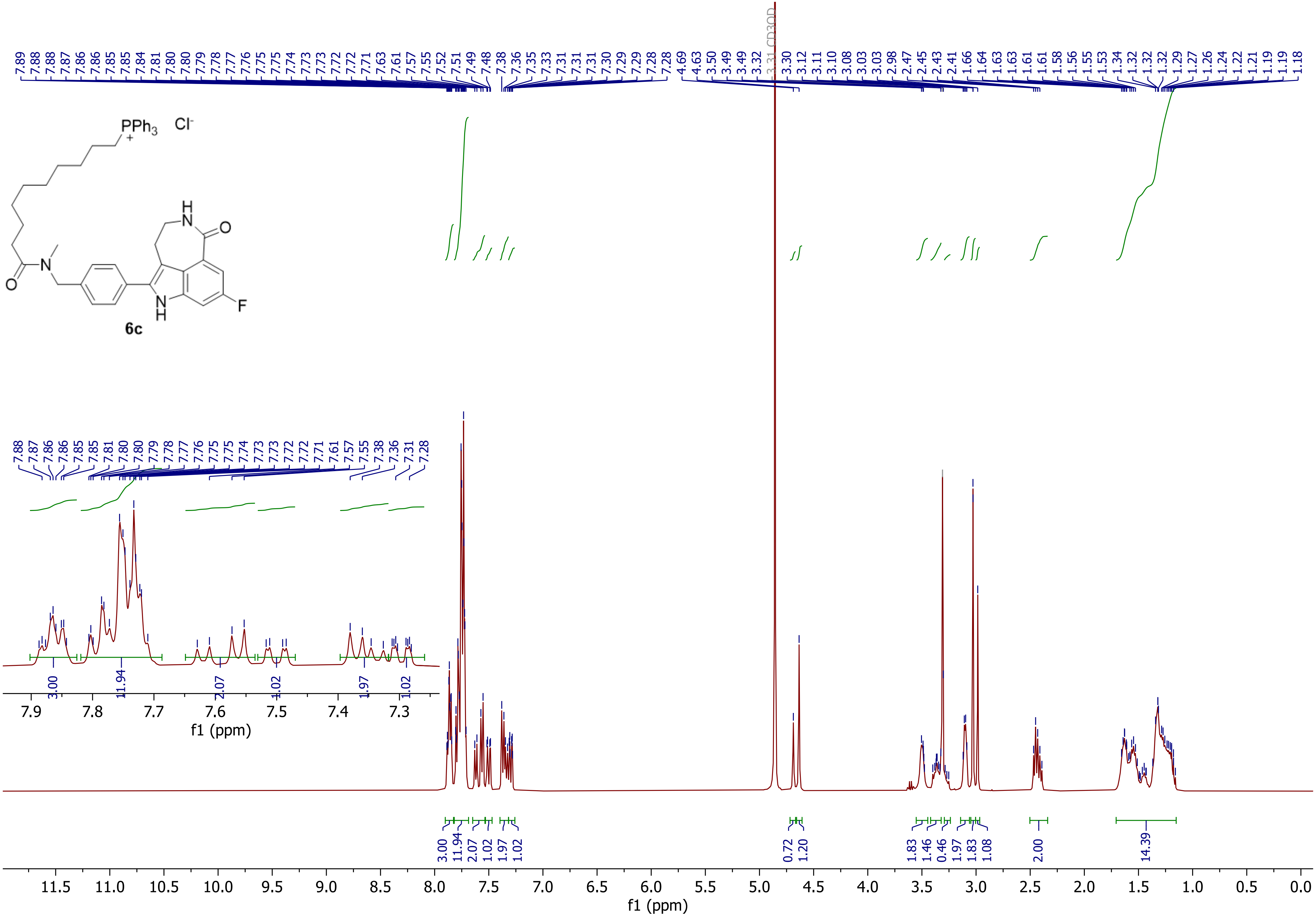
7.89 7.88 7.88 7.87 7.86 7.86 7.85 7.85 7.84 7.81 7.80 7.80 7.79 7.78 7.77 7.76 7.75 7.75 7.74 7.73 7.73 7.72 7.72 7.71 7.63 7.61 7.57 7.55 7.52 7.51 7.49 7.48 7.38 7.36 7.35 7.33 7.31 7.31 7.31 7.30 7.29 7.29 7.28 7.28 4.69 4.63 3.50 3.49 3.49 3.32 3.31 CD3OD 3.30 3.12 3.11 3.10 3.08 3.03 3.03 2.98 2.47 2.45 2.43 2.41 1.66 1.64 1.63 1.63 1.61 1.61 1.58 1.56 1.55 1.53 1.34 1.32 1.32 1.32 1.29 1.27 1.26 1.24 1.22 1.21 1.19 1.19 1.18
PPh3 Cl-
6c
3.00 11.94 2.07 1.02 1.97 1.02 0.72 1.20 1.83 1.46 0.46 1.97 1.83 1.08 2.00 14.39
f1 (ppm)

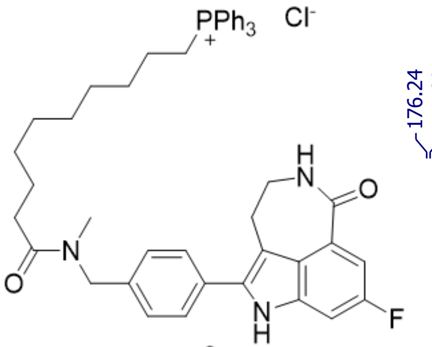
PPh3
Cl⁻
N
H
O
O
N
N
H
F

6c

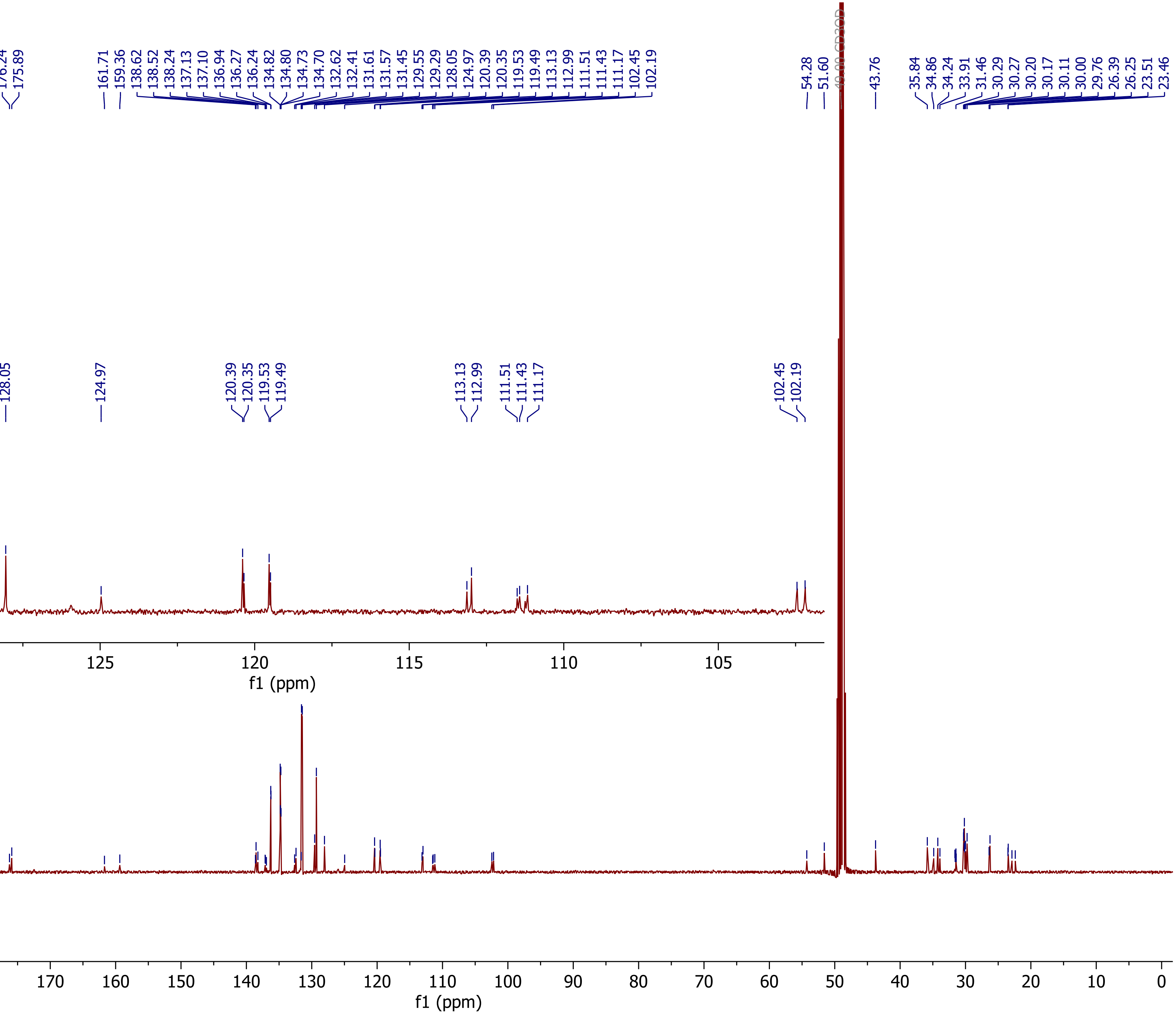
176.24
175.89
161.71
159.36
138.62
138.52
138.24
137.13
137.10
136.94
136.27
136.24
134.82
134.80
134.73
134.70
132.62
132.41
131.61
131.57
131.45
129.55
129.29
128.05
124.97
120.39
120.35
119.53
119.49
113.13
112.99
111.51
111.43
111.17
102.45
102.19
54.28
51.60
49.00 CD3OD
43.76
35.84
34.86
34.24
33.91
31.46
30.29
30.27
30.20
30.17
30.11
30.00
29.76
26.39
26.25
23.51
23.46
135
130
125
120
115
110
105
f1 (ppm)
210
200
190
180
170
160
150
140
130
120
110
100
90
80
70
60
50
40
30
20
10
0
f1 (ppm)

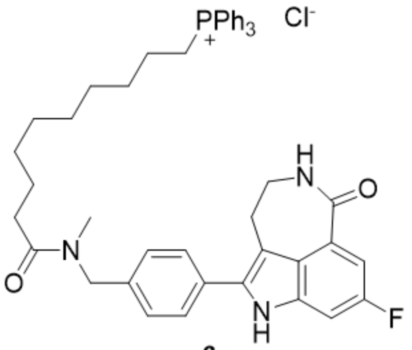
PPh3 Cl-
+
H
N
O
N
O
F
N
H

6c

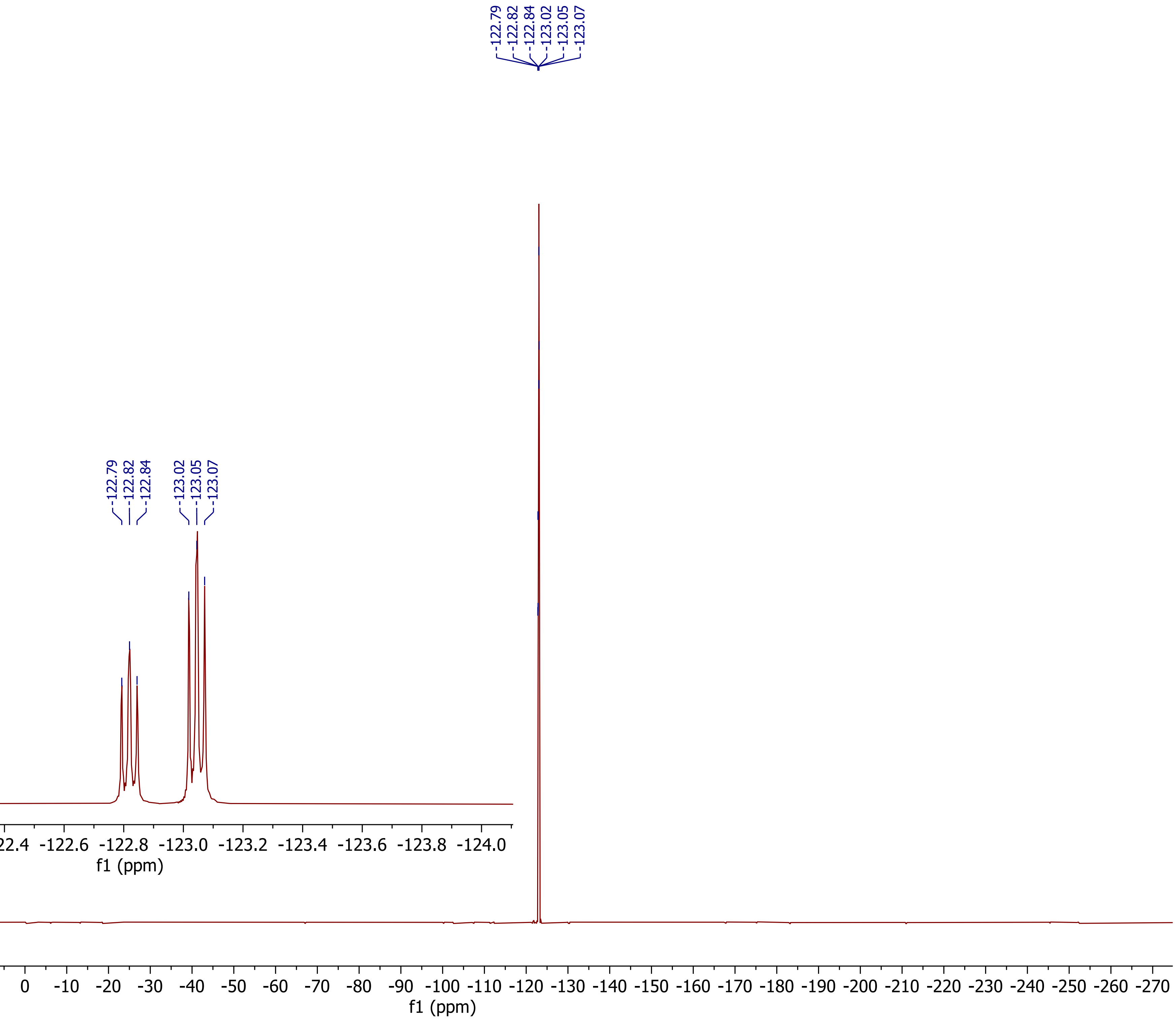
-122.79
-122.82
-122.84
-123.02
-123.05
-123.07
-121.8 -122.0 -122.2 -122.4 -122.6 -122.8 -123.0 -123.2 -123.4 -123.6 -123.8 -124.0
f1 (ppm)
70 60 50 40 30 20 10 0 -10 -20 -30 -40 -50 -60 -70 -80 -90 -100 -110 -120 -130 -140 -150 -160 -170 -180 -190 -200 -210 -220 -230 -240 -250 -260 -270
f1 (ppm)

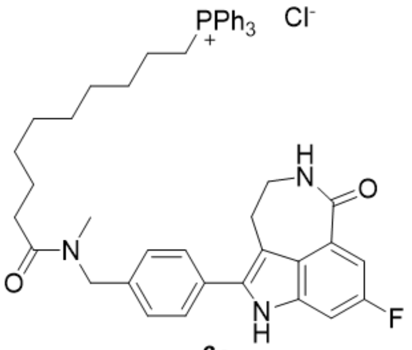
PPh3 Cl-
H
N
O
N
O
N
H
F

6c

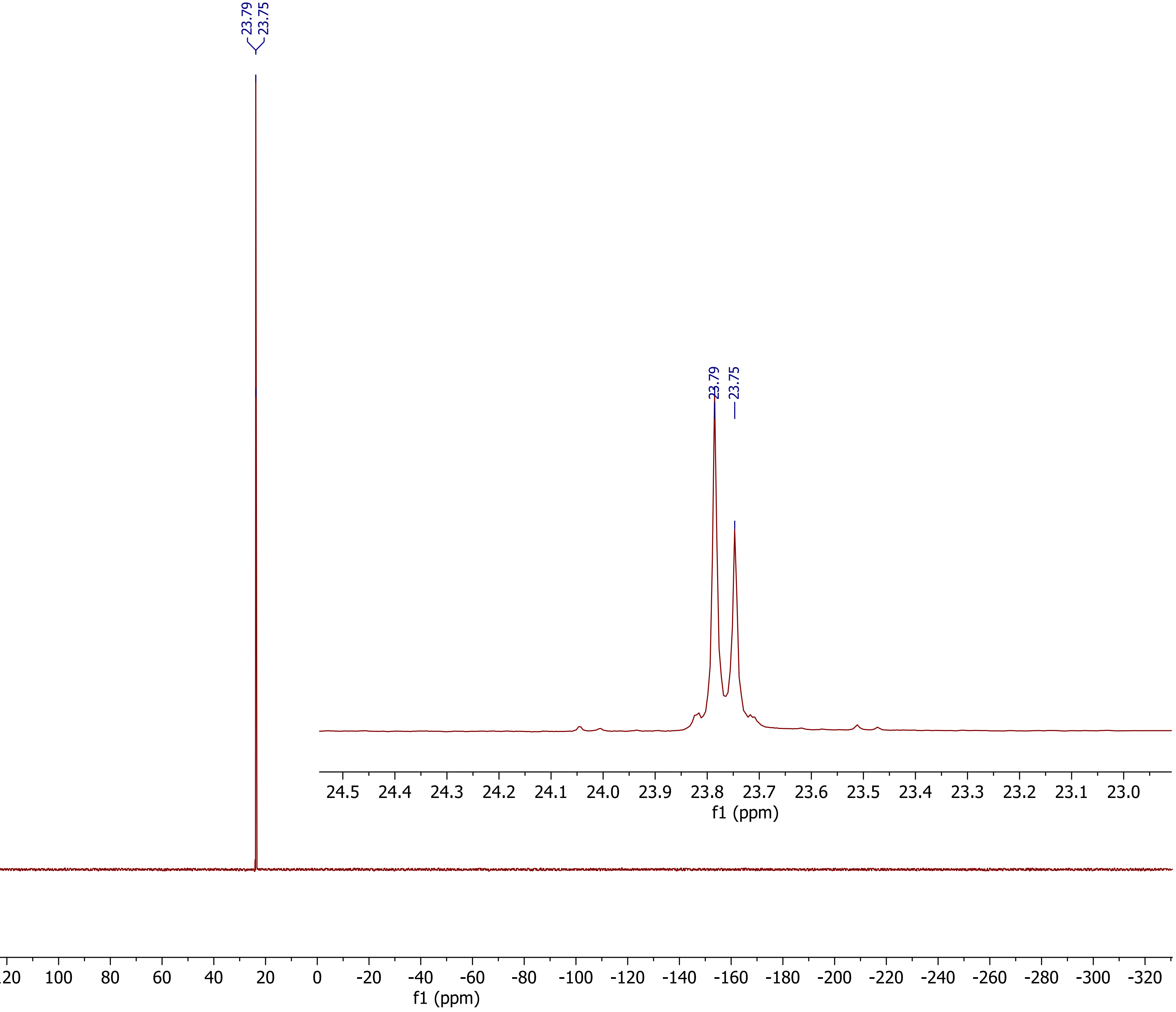
23.79
23.75
24.5 24.4 24.3 24.2 24.1 24.0 23.9 23.8 23.7 23.6 23.5 23.4 23.3 23.2 23.1 23.0
f1 (ppm)
220 200 180 160 140 120 100 80 60 40 20 0 -20 -40 -60 -80 -100 -120 -140 -160 -180 -200 -220 -240 -260 -280 -300 -320
f1 (ppm)
